# Conservation and plasticity of IncHI2 plasmid-borne metal resistance loci in *Salmonella enterica* Serovar Heidelberg

**DOI:** 10.3389/fmicb.2026.1968489

**Published:** 2026-10-28

**Authors:** Ahmed F. Hikal, Danielle Sopovski, Javier Revollo, Hanka Brangsch, Steven L. Foley, Ashraf A. Khan

**Affiliations:** 1 Division of Microbiology, National Center for Toxicological Research, U. S. Food and Drug Administration, Jefferson, AR, United States; 2 Department of Bacteriology, Immunology and Mycology, College of Veterinary Medicine, Benha University, Toukh, Egypt; 3 Division of Genetic and Molecular Toxicology, National Center for Toxicological Research, U. S. Food and Drug Administration, Jefferson, AR, United States; 4 Institute of Bacterial Infections and Zoonoses, Friedrich-Loeffler-Institute, Jena, Germany

**Keywords:** antimicrobial resistance, copper resistance, heavy metal resistance, IncHI2 plasmid, *S*. Heidelberg

## Abstract

The incompatibility group (Inc) HI2 plasmid is frequently detected in *Salmonella enterica* serovar Heidelberg and is commonly associated with heavy metal and multidrug resistance genes. In this study, we assessed the characteristics of IncHI2A plasmids of eight copper-resistant *Sal.* Heidelberg isolates from animal and human sources. A comparative analysis of the genetic architecture of these plasmids with that of the parent plasmid R478 from *Serratia marcescens* and the reference plasmid pSH111_227 from *Sal.* Heidelberg revealed high conservation of the plasmid backbone, as well as copper/silver and tellurium resistance cassettes. Notably, the gene encoding the periplasmic silver-binding protein SilF was present in all *Sal.* Heidelberg isolates but was absent in R478, whereas the tellurite resistance gene *terF* was absent in all isolates. Additionally, except for two isolates, the IncHI2A plasmids lacked mercury resistance genes found in the parental plasmid R478. Tetracycline and aminoglycoside resistance genes are conserved among the IncHI2A plasmids of all *Sal.* Heidelberg isolates and the reference plasmids. In addition, all *Sal.* Heidelberg isolates included neomycin-family aminoglycoside resistance genes, which conferred resistance to neomycin and kanamycin. Analysis of the non-IncHI2A plasmids in these isolates showed that the majority of the isolates carried multidrug resistance (MDR) genes, whereas three isolates did not contain any detectable MDR genes. In conclusion, these variations within the heavy metal resistance (HMR) loci underscore the dynamic nature of IncHI2A plasmids and their potential role in the acquisition and retention of these genes under selective pressure.

## Introduction

*Salmonella enterica* serovar Heidelberg is one of the most common causes of foodborne illness among *Sal. enterica* serovars and has been associated with several outbreaks in the United States, resulting in serious clinical symptoms, hospitalization, and mortality (Nichols et al., 2022; Vugia et al., 2004; Chittick et al., 2006; Centers for Disease Control and Prevention (CDC), 2013). *Sal. enterica* serovar Heidelberg (*Sal.* Heidelberg) is frequently isolated from diseased poultry and is disproportionately associated with invasive infections in humans compared with other chicken-derived *Salmonella* serovars (Foley et al., 2008; Jones et al., 2008). In addition to its public health importance, *Sal*. Heidelberg is known for the acquisition of mobile genetic elements, which contribute to pathogenicity, environmental persistence, and antimicrobial resistance (AMR) (Han et al., 2012; Kaldhone et al., 2008; Gokulan et al., 2013).

IncHI2 plasmids are large (>200 kb), conjugative plasmids found mainly in the Enterobacteriaceae family, and they have gained significant medical interest due to their ability to carry AMR, biocide/disinfectant resistance, and heavy metal resistance (HMR) genes, including those conferring resistance to copper, silver, and tellurite (Algarni et al., 2024; Gilmour et al., 2004; Fang et al., 2016). The extensive use of copper as an antimicrobial compound and growth promoter in animal feed has led to the acquisition of copper−/silver-resistant genes (*pco* and *sil* clusters) by *Sal. enterica* serovars to aid their persistence in toxic environmental conditions (Hikal et al., 2024). These clusters are commonly associated with multidrug resistance genes (Vats et al., 2022), raising concerns about the spread of AMR genes. Tellurite is another toxic heavy metal that is harmful to bacteria and is commonly used in batteries, wires, and thermoelectric equipment as a semiconductor (He et al., 2017). Tellurite has historically been used as a disinfectant due to its high bactericidal activity (Chasteen et al., 2009; Munn and Hopkins, 1925). To overcome its toxicity, bacteria, specifically members of the Enterobacteriaceae family, have acquired the core tellurite resistance locus (*terZABCDEF*) on IncHI2 plasmids (Han et al., 2012; Fang et al., 2016).

Although IncHI2 plasmids carry copper/silver resistance and tellurite resistance genes on the same plasmid backbone, the structural organization of these loci in *Sal.* Heidelberg remains unclear. Therefore, comparative genetic analysis of IncHI2 plasmids derived from *Sal.* Heidelberg with the reference plasmid R478 from *Serratia marcescens* (*Ser.* marcescens) is essential for understanding plasmid plasticity and distinguishing the conserved loci from the variable accessory regions. We selected R478 as the reference for our IncHI2 plasmid comparisons because of its complete sequence and metal-resistance regions, including the *ter*, *sil/pco*, and *mer* loci, have been well characterized (Gilmour et al., 2004; Fang et al., 2016; Whelan et al., 1995).

In this study, we performed whole-genome sequencing (WGS) of eight highly copper-resistant *Sal.* Heidelberg isolates recovered from turkey, ground meat, and humans between 1993 and 2008. We then compared their IncHI2 plasmid sequences with the reference plasmid R478 from *Ser.* marcescens and pSH111_227 from *Sal.* Heidelberg strain 111. We focused on copper/silver resistance, tellurite resistance, and AMR genes to better understand how these genes are organized and diversified between IncHI2 plasmids.

Identifying the diversity and organization of HMR and AMR genes in these plasmids will improve our understanding of plasmid evolution and support genomic surveillance, public health risk assessment strategies, and food-animal industry practices, particularly with respect to the environmental persistence of *Sal.* Heidelberg strains carrying these plasmids.

## Materials and methods

### Bacterial strains and growth conditions

Eight strains of highly copper-resistant *Sal.* Heidelberg were isolated from swine, cattle, turkey, ground beef, and human stool. We had more than 50 *Sal.* Heidelberg isolates recovered from several states, but we selected these 8 isolates based on their resistance to toxic levels of copper. The minimum inhibitory concentration of copper was determined using Luria-Bertani (LB) agar (Sigma-Aldrich, St. Louis, MO, United States) supplemented with CuSO_4_. Isolates were inoculated in LB broth, serially diluted, and spotted onto LB agar supplemented with CuSO_4_ (2, 3, 4, and 5 mM). These concentrations were selected based on our previous study (Hikal et al., 2024), which indicated that neither wild-type *Salmonella enterica* nor the *pco* mutant was able to grow in medium supplemented with these levels of copper. Plates were incubated at 37 °C for 48 h anaerobically to facilitate the conversion of the non-toxic form of copper (Cu II) to the toxic form (Cu I). An anaerobic environment was generated using Mitsubishi AnaeroPack-Anaero (Thermo Fisher Scientific, Waltham, MA, United States).

### Genome sequencing and assembly

For WGS, *S.* Heidelberg isolates were streaked onto sheep’s blood agar plates (Remel, Lenexa, KS, United States). Single colonies were picked and grown overnight in LB medium. Genomic DNA was extracted using the DNeasy Blood and Tissue Kit (Qiagen, Hilden, Germany). The DNA was quantified using the Qubit 4 Fluorometer (Invitrogen, Thermo Fisher Scientific, Waltham, MA, United States) with the Qubit DNA HS Assay Kit. The DNA quality was assessed using the NanoDrop One (Thermo Fisher Scientific, Waltham, MA, United States). The isolates were sequenced using Oxford Nanopore Technologies (ONT, Oxford, United Kingdom) and MiSeq (Illumina, San Diego, CA, United States) platforms.

For ONT long-read sequencing, the DNA library was constructed using the Rapid Barcoding Sequencing Kit from Oxford Nanopore Technologies (CAT: SQK-RBK004) according to the manufacturer’s instructions. Sequencing was carried out on a MinION Mk1c sequencer using a 9.4.1 Flow Cell (FLO-MIN106D). The sequencing protocol ran for 72 h under standard conditions (180 mV at 37 °C). Sequencing data were processed as per the suggested protocol using MinION Guppy software v6.5.7 with the following modifications. The FastCall protocol, FLO-MIN106, was used concurrently with sequencing. Reads were base-called and demultiplexed, the barcodes were trimmed, and the sequences were removed if they had a Q-score less than 8. The barcodes that were internally joined were removed.

For short-read sequencing, the DNA libraries were constructed using the Illumina DNA Prep Kit and multiplexed using the IDT for Illumina DNA/RNA UD Indexes set according to the manufacturer’s instructions.

ONT long reads were assembled using Autocycler version 0.4.0 with default settings (Wick et al., 2025). Autocycler incorporated assemblies generated with Canu, Flye, Miniasm, NextDenovo, Plassembler, and Raven and merged them to create a consensus assembly. Illumina short reads from the corresponding sample were subsequently used to polish each assembly using Polypolish v0.6.0 (Wick and Holt, 2022).

### Genome analysis

Multilocus sequence typing was performed using mlst v2.23.0 (Jolley et al., 2018) with the Achtman 7 Gene MLST scheme (“senterica_achtman_2”) (Kidgell et al., 2002) available from PubMLST (Jolley et al., 2018). The assemblies were screened for AMR and metal resistance genes using AMRFinderPlus v 3.11.26 (Feldgarden et al., 2021) with the option -O Salmonella and using ABRicate v1.0.1(GitHub Repository, 2020) in combination with the CARD database (Alcock et al., 2023). ABRicate was further used to screen for virulence factors using the Virulence Factor Database (VFDB) gene set A (Chen et al., 2005). For predicting plasmid-borne contigs, PlasmidFinder v2.1.6 was used (Carattoli et al., 2014).

### Plasmids conservation analysis and sequence comparison

The level of conservation of IncHI2A plasmid sequences was evaluated against the reference plasmids R478 (GenBank accession no. BX664015) and pSH111_227 (accession no. NC_019114) using BLASTN v2.14.1 + with the megablast algorithm (Camacho et al., 2009; Zhang et al., 2000). IncHI2A sequences were aligned pairwise to each reference plasmid. High-scoring segment pairs (HSPs) were retained if they had ≥80% nucleotide identity, an alignment length of ≥500 bp, and an E-value ≤1e-20. To avoid counting overlapping regions more than once, HSPs were ranked by bit score and alignment length, and only newly covered query regions were included in the summary statistics. Weighted nucleotide identity and query coverage were subsequently calculated from the retained BLAST high-scoring segment pairs using a custom Python 3 script (Supplementary File 1). The conservation score was calculated as the number of identical bases in the aligned query regions divided by the total query length. The results were visualized as an SVG heatmap using a custom Python 3 script (Supplementary File 1), and the final figure was formatted using Inkscape v1.4.4. Plasmid sequence comparison was performed using Proksee software^1^ (Grant et al., 2023) by uploading the GenBank-formatted.gbk file for each plasmid to the web portal. For comparative analysis of the *pco* and *sil* clusters, these regions were extracted and compared to the *pco/sil* cassette of the reference plasmids using Clinker v0.032 (Gilchrist and Chooi, 2021). Clinker performs pairwise protein-sequence alignments of the translated coding sequence.

### Plasmid typing

Prediction of plasmid type was assessed using MOB-suite software based on Mash distances and complete linkage clustering (Robertson et al., 2020). Primary and secondary clusters were assigned using Mash distance thresholds of 0.06 and 0.025, respectively.

## Results and discussion

In this study, eight *Sal*. Heidelberg isolates recovered from food and humans between 1993 and 2008 showed resistance to toxic levels of CuSO_4_ (4 mM), whereas control *Sal.* Heidelberg isolates (NCTR70, NCTR85, and NCTR708) from the same collection lacked copper resistance genes and did not exhibit copper resistance (Supplementary Figure S1). Although copper is essential for bacterial growth, excess copper is toxic because free intracellular copper can react with hydrogen peroxide to generate hydroxyl radicals, which can damage DNA, lipids, and proteins (Osman and Cavet, 2008). In our previous study, we showed that *Sal enterica* strains lacking the copper resistance (*pco*) cluster failed to grow in Cu-supplemented media only when incubated under anaerobic conditions, under which the less toxic Cu (II) form is reduced to the more toxic Cu (I) form (Hikal et al., 2024). Therefore, we used the same growth conditions in this study to assess copper resistance in these isolates. To examine the genetic background of copper resistance and elucidate the presence of other heavy metal resistance genes in these isolates, we performed WGS and deposited the sequence data in GenBank under BioProject accession number PRJNA1294273. Detailed assembly statistics, including contig lengths, sequence coverage, and assembly completeness parameters, are shown in Supplementary Files 2, 3. Our *in silico* plasmid prediction analysis revealed that all *Sal*. Heidelberg isolates harbored plasmids approximately 231–261 kb in size. The predicted plasmids in *Sal.* Heidelberg isolates were classified as conjugative IncHI2A by MOB-suite (Robertson and Nash, 2018), whereas pSH111_227 was classified as IncHI2 with a RepFIB replication protein (Supplementary Table S1), which supports the possible evolutionary relatedness of these plasmids in *Sal*. Heidelberg. The Proksee alignment of the predicted IncHI2A plasmids with the reference R478 plasmid from *Ser. marcescens* and pSH111_227 from *Sal*. Heidelberg showed a higher percentage of conservation (>89% identity; Figures 1A,B) and a greater coverage of pSH111_227 compared to R478 in all isolates, except NCTR718 and NCTR719 (Figure 2). Genetic analysis of the copper/silver resistance (*pco/sil*) operons showed that the IncHI2 plasmids of all *Sal*. Heidelberg isolates and pSH111_227 carried the complete *pco* (*pcoABCDERSE*) and *sil* (*silESRCFBAP*) cassettes (Figure 3). In contrast, the parent plasmid R478 lacks *silF*. SilF is a periplasmic silver-binding metallochaperone involved in silver resistance in Gram-negative bacteria (Randall et al., 2015). Arrault et al. have shown that SilF interacts with SilB even in the absence of silver and, upon silver binding, forms a transient SilF–Ag^+^–SilB complex that leads to silver transfer to the SilB/SilA efflux system (Arrault et al., 2023). Deletion of *silF* in the *cusCFBA*-negative background, where the redundant silver efflux system was absent, completely abolished silver resistance in *E. coli* (Randall et al., 2015). *In silico* screening detected *cusF* in plasmid R478 (data not shown), indicating that this gene complements the loss of *silF*.

**Figure 1:**
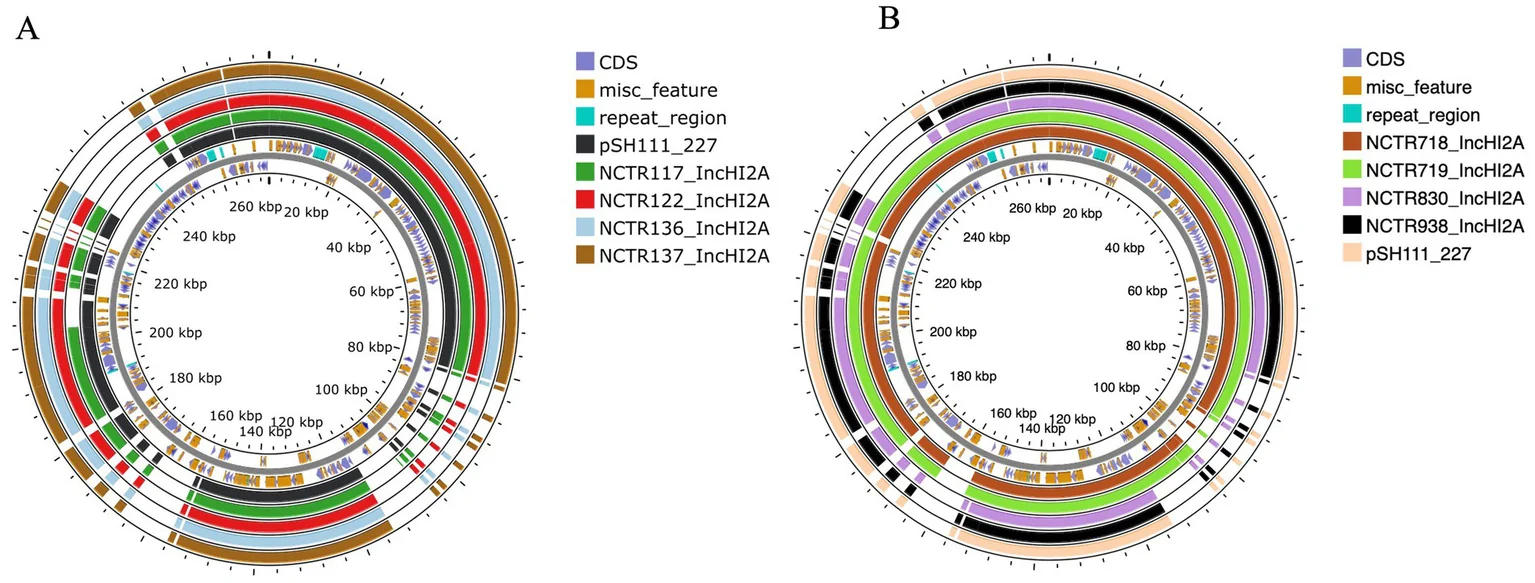
Proksee comparisons of predicted IncHI2A plasmids from NCTR isolates with the reference plasmids R478 and pSH111_227 (**A**–**B**). R478 is shown as the central reference plasmid, with pSH111_227 and the NCTR plasmids displayed as colored rings around it. Colored arrows in the center indicate the coding sequences (CDSs), miscellaneous features, and repeat regions of R478, as identified in the map legend.

**Figure 2:**
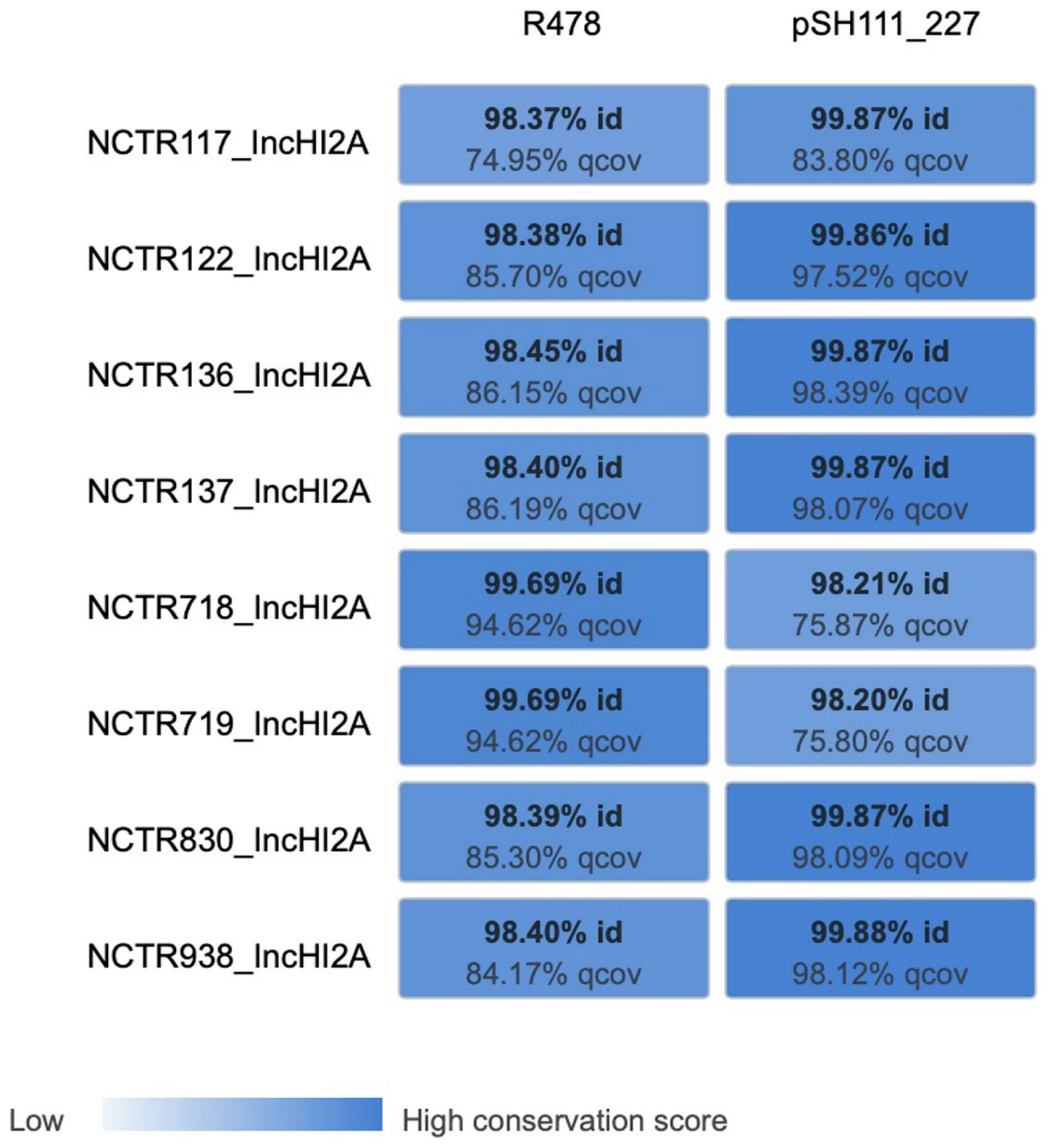
Conservation of NCTR isolates’ IncHI2A plasmids against plasmids R478 and pSH111_227. Cell color = conservation score (% identical query bases). Text = weighted identity/query coverage.

**Figure 3:**
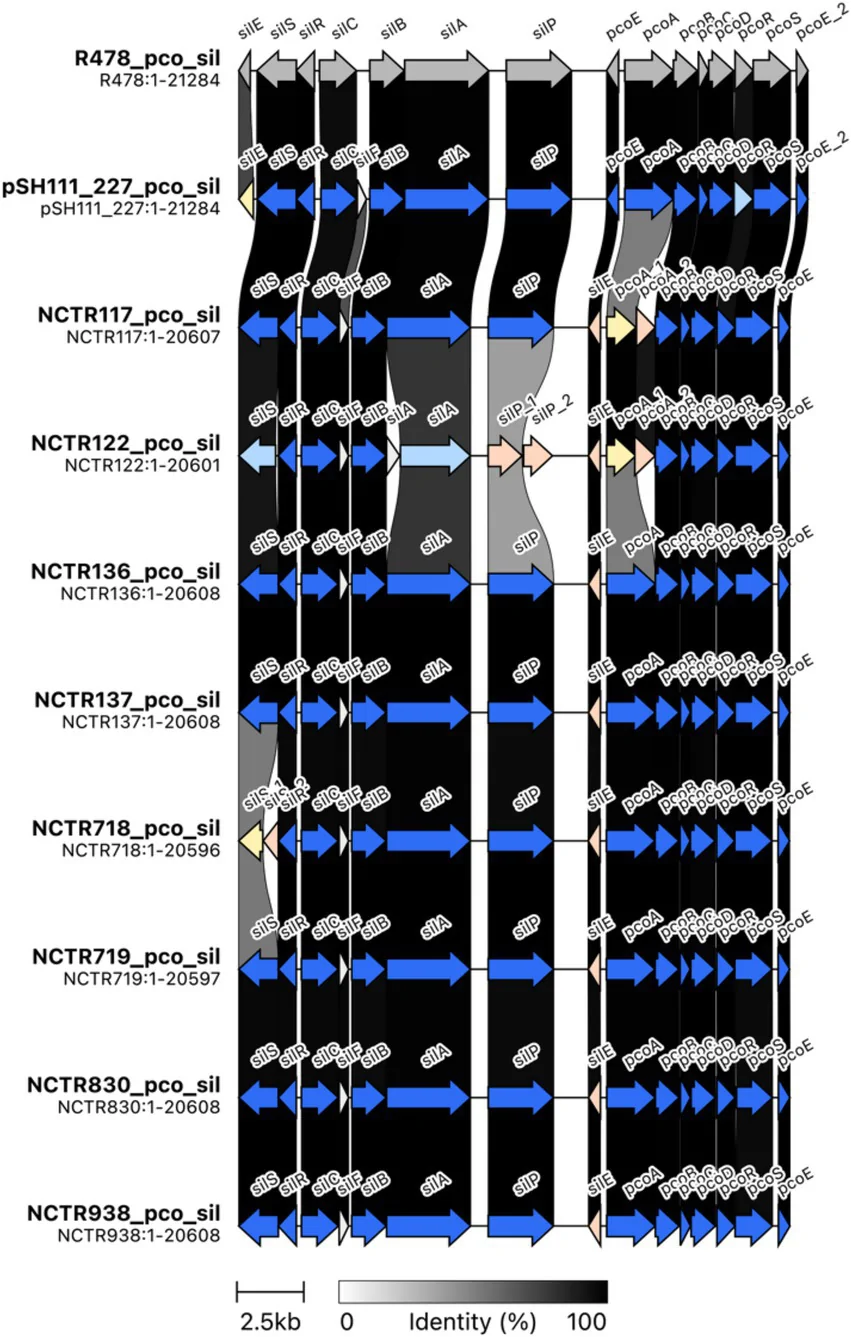
Genetic context of the *pco* and *sil* clusters of the NCTR isolates compared to plasmid R478 and pSH111_227. Arrows represent gene orientation and annotated coding sequences. Homologous genes are linked by shaded blocks, with darker shading indicating higher protein sequence identity.

Additionally, *Sal*. Heidelberg isolates carried tellurium resistance genes (*terABCDEWXYZ*) (Figure 4). Interestingly, however, comparative genetic analysis of these isolates with the reference plasmids R478 and pSH111_227 showed that all eight *Sal*. Heidelberg isolates lacked the *terF* gene, which is conserved in both reference plasmids. Although *terF* is frequently associated with tellurite resistance loci on IncHI2 plasmids (Han et al., 2012; Fang et al., 2016; García Fernández et al., 2007), it is missing in our *Sal*. Heidelberg isolates. Thus, these IncHI2A plasmids, which share most of the backbone of the parent plasmid R478, are genetically distinct. The TerF protein is predicted to contribute to a membrane-associated metal−/stress-response complex within the *ter* cluster (Anantharaman et al., 2012); however, there is no experimental evidence indicating that it plays a role in tellurite resistance, whereas *terZABCDE* have been more strongly associated with resistance (Toptchieva et al., 2003; Nguyen et al., 2021). We also searched for the presence of mercury resistance genes in the predicted IncHI2A plasmids in the *Sal*. Heidelberg isolates. Unlike plasmid R478, all isolates, except NCTR718 and NCTR719, lacked *mer* genes (Supplementary Table S2). These findings are consistent with a previous study that showed a low prevalence of these genes in IncHI2 plasmids detected in *Salmonella* compared to *Enterobacter* (Algarni et al., 2024). We next examined the AMR genes on the IncHI2A plasmids in the *Sal*. Heidelberg isolates. Our data showed that all plasmids shared aminoglycoside and tetracycline resistance genes (Figure 5). We identified the aminoglycoside resistance genes *neo_1* and *neo_2* in these isolates. These genes are homologous to the *aphA/aph(3″)* genes of the aminoglycoside phosphotransferase family and are known to confer resistance to kanamycin and neomycin. These data suggest that aminoglycoside resistance genes are conserved within these plasmids. Interestingly, the IncHI2A plasmid of NCTR117 carried different AMR genes than those in the rest of the isolates, such as *tetM*, *dhfrI*, *aadA_1*, and *aadA_2*. This suggests that NCTR117 may have acquired an additional mobile AMR cassette and supports the accessory-region plasticity among IncHI2A plasmids. In addition to IncHI2A plasmids, *Sal*. Heidelberg isolates also contained other plasmids belonging to different incompatibility groups, such as IncC, IncX1, IncI, and IncI1, with sizes ranging from approximately 37 to 170 kb in contig 3 (Table 1). MOB-suite whole-sequence typing resolved these plasmids into five primary/secondary clusters based on comparison with the MOB-suite reference plasmid database, where primary clusters represent broader plasmid groups and secondary clusters represent more closely related subgroups (Table 1). NCTR117 and NCTR136 had the same AA860/AJ275 cluster, whereas NCTR718 and NCTR719 shared the AB233/AJ927 cluster, indicating whole-sequence relatedness within each pair. On the other hand, NCTR137 belonged to AB230/AJ911. NCTR830 and NCTR938 shared only the primary cluster but differed in the secondary cluster. MOB-suite failed to assign any cluster for NCTR122. All plasmids were predicted as conjugative based on the presence of relaxase and mating-pair formation (MPF) systems (Robertson and Nash, 2018). AMRFinder and CARD analysis identified several AMR genes in the majority of them (Figure 6). Intriguingly, NCTR718 (IncX1 plasmid), NCTR719 (IncX1 plasmid), and NCTR938 (IncI1 plasmid) lacked AMR genes, which were detected in the other isolates. Previous studies reported that IncX1 in *Sal*. Heidelberg can occur without AMR genes (Oladeinde et al., 2021), whereas IncI1 plasmids are frequently associated with AMR (Han et al., 2012; Robertson et al., 2023). This phenomenon was observed among these isolates and may reflect either loss of AMR genes or acquisition of plasmid backbones lacking AMR-associated genes.

**Figure 4:**
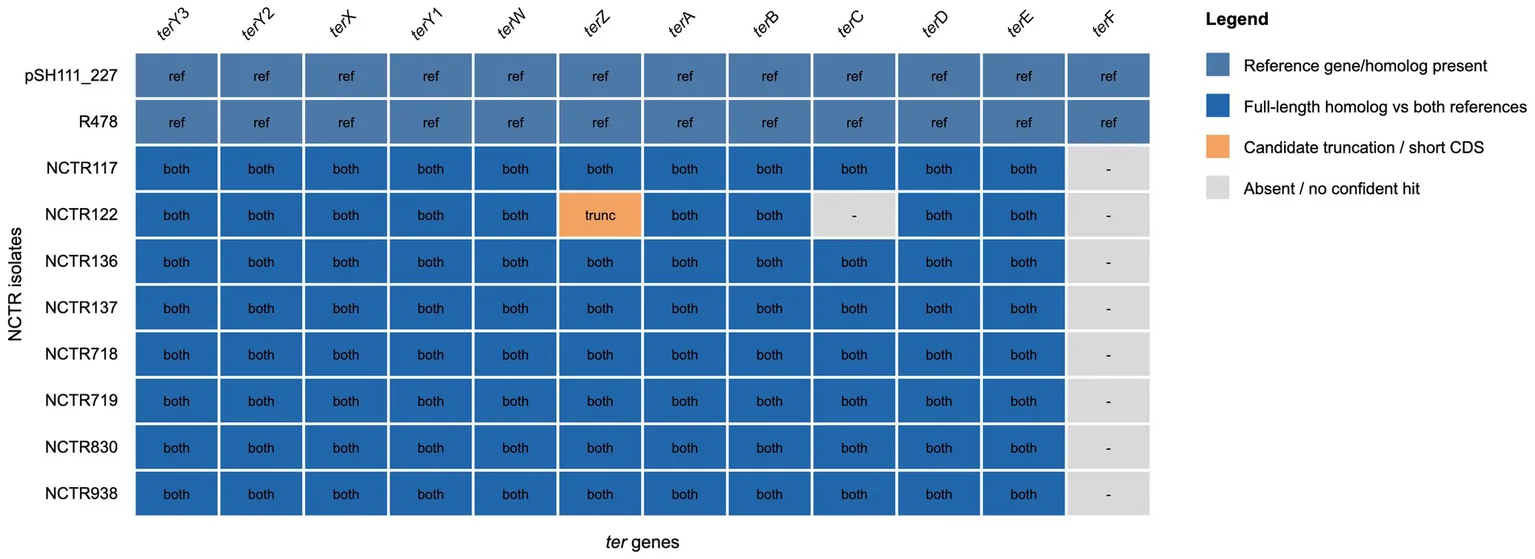
*ter* gene homologs in NCTR isolates compared with pSH111_227 and R478. Color indicates the best homolog call from Clinker alignments; orange indicates candidate truncation by CDS length.

**Figure 5:**
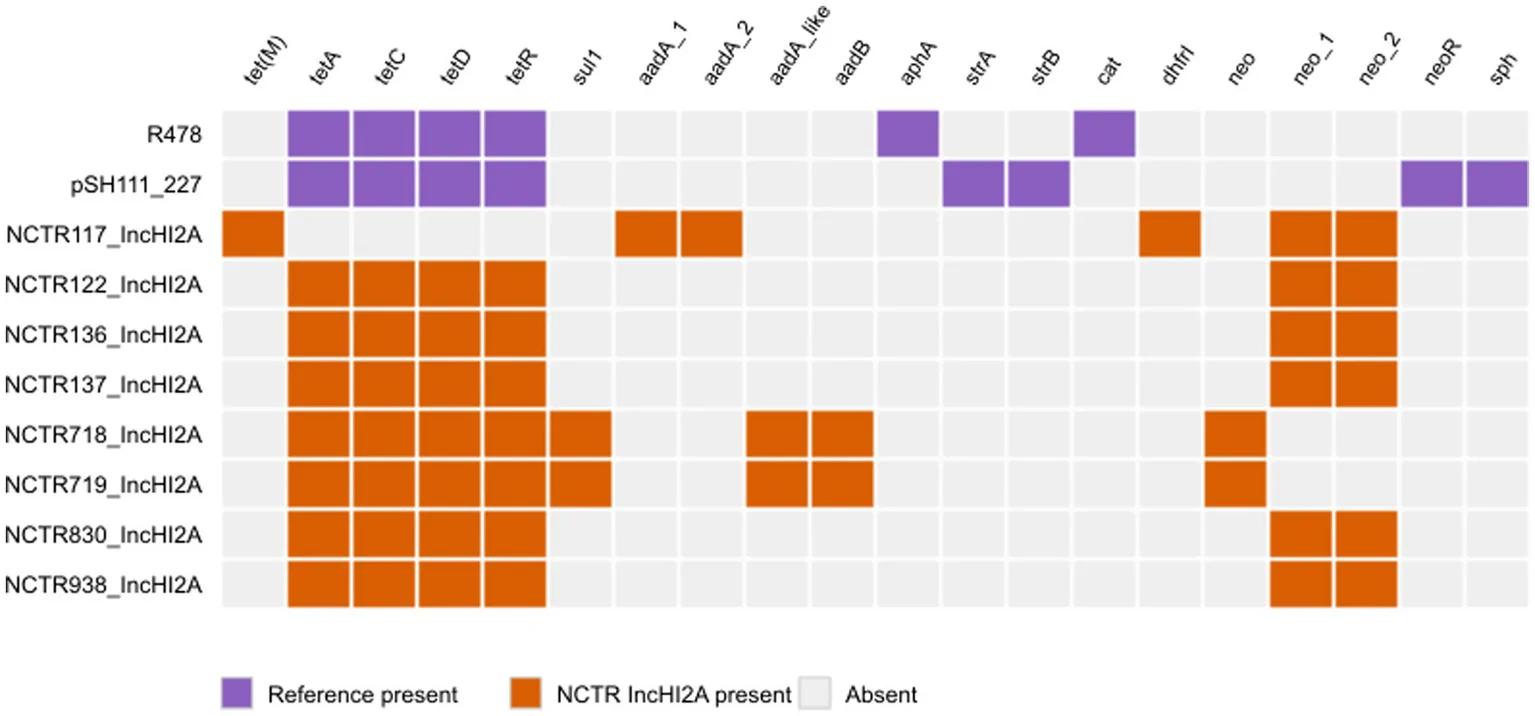
AMR genes detected in IncHI2A plasmids in *S*. Heidelberg isolates compared to R478 and pSH111_227 plasmids.

**Table 1:** ^*^Non-IncHI2A plasmid types across NCTR isolates.

Isolate	Plasmid type	Size	Mobility	Cluster
NCTR117	IncC	169,950 bp	Conjugative	AA860/AJ275
NCTR122	IncC	149,085 bp	Conjugative	None were detected
NCTR136	IncC	183,217 bp	Conjugative	AA860/AJ275
NCTR137	IncX1	62,705 bp	Conjugative	AB230/AJ911
NCTR718	IncX1	37,696 bp	Conjugative	AB233/AJ927
NCTR719	IncX1	37,697 bp	Conjugative	AB233/AJ927
NCTR830	IncI-gamma/K1	106,825 bp	Conjugative	AA474/AI596
NCTR938	IncI1/B/O	82,878 bp	Conjugative	AA474/AI602

*Plasmid types are based on MOB-suite replicon/mobility classification. We used colores to highlight the fact that NCTR isolates carry the same plasmid types, such as IncC and IncX1.

**Figure 6:**
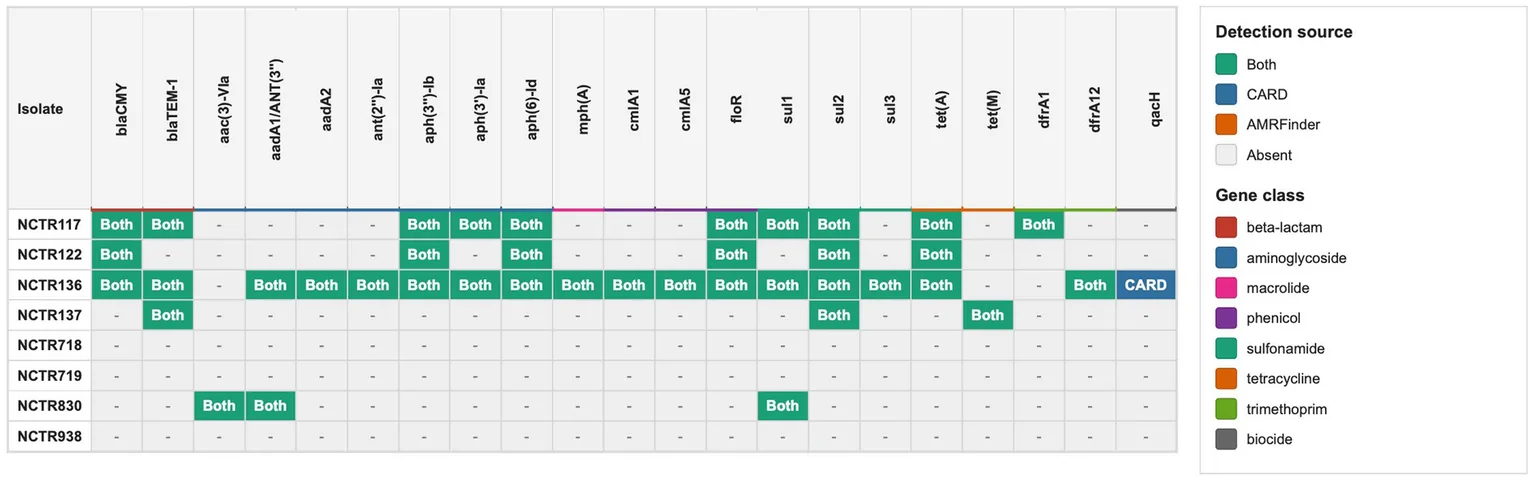
CARD and AMRFinderPlus AMR genes detected in non-IncHI2A plasmids across NCTR isolates.

This study has several limitations. First, we included only eight copper-resistant *Sal*. Heidelberg isolates recovered from limited sources in the United States over a relatively short period of time (1993–2008); therefore, these findings may not represent the full distribution and diversity of IncHI2A plasmids in *Sal.* Heidelberg. Second, plasmid comparisons and analyses of AMR and HMR genes were solely based on *in silico* analysis, except for copper resistance, so the predicted functions of the identified genes require experimental confirmation. Third, although all the IncHI2A and non-IncHI2A plasmids in *Sal.* Heidelberg isolates were predicted to be conjugative, plasmid conjugation was not experimentally tested. These limitations should be taken into consideration when interpreting the association between HMR and AMR genes.

In conclusion, we performed comparative genetic analysis of eight copper-resistant *Sal*. Heidelberg isolates recovered from human, animal, and food sources in the United States between 1993 and 2008. Our study highlights the plasticity and diversity of resistance determinants among the plasmids detected in these isolates. Comparative analysis with the reference plasmids R478 from *Ser.* marcescens and pSH111_227 from *Sal*. Heidelberg showed that all isolates carried IncHI2A plasmids with variable HMR and AMR genes. The differences observed in the HMR loci support the dynamic nature and plasticity of IncHI2A plasmids and their potential contribution to the acquisition and maintenance of HMR genes under selective pressure. The presence of HMR and AMR genes within the conjugative IncHI2A plasmids may contribute to the persistence and spread of these resistance plasmids in *Sal*. Heidelberg. Together, this study enhances our understanding of the nature and plasticity of the IncHI2A and non-IncHI2A plasmids in *Sal*. Heidelberg and underscores the need for continued genomic surveillance to better understand their evolution and diversity.

## Data Availability

The datasets presented in this study can be found in online repositories. The names of the repository/repositories and accession number(s) can be found in the article/Supplementary material.

## Footnotes

1
https://proksee.ca

## Supplementary material

Supplementary Table S1 IncHI2A Plasmid types detected in NCTR isolates based on MOB-suite replicon/mobility classification.

Supplementary Table S2 Mercury resistance genes in NCTR IncHI2A plasmids and reference plasmids.
