# Supplementary material for: Conservation and plasticity of IncHI2 plasmid-borne metal resistance loci in Salmonella enterica Serovar Heidelberg
Source: Front Microbiol. 2026 Oct 28;17:1968489. doi: 10.3389/fmicb.2026.1968489 (PMC13752096; doi:10.3389/fmicb.2026.1968489)
Supplement: Supplementary file 1 [file Supplementary_file_1.pdf]

Supplementary File: Custom Python code used for plasmid and resistance-gene analyses
Generated for inclusion in supplementary information
Generated date: 2026-09-13

Overview
This file contains the custom Python 3 scripts generated for this project. The main IncHI2A conservation script performs the BLASTN/megablast-based pairwise comparison against reference plasmids R478 and pSH111_227, filters high-scoring segment pairs (HSPs), removes redundant query overlap, calculates weighted nucleotide identity, query/reference coverage, and conservation score, and writes an SVG heatmap. Additional scripts support reference-gene heatmaps, pco/sil region handling, Clinker output post-processing, MOB-suite table generation, and AMR/metal-resistance summary visualizations.

Key filters and calculations implemented in contig2_reference_conservation.py
- BLASTN task: megablast
- Retained HSPs: percent identity >= 80, alignment length >= 500 bp, E-value <= 1e-20
- HSP prioritization: descending bit score, then descending alignment length
- Overlap handling: only newly covered query bases contribute to summary statistics
- Weighted nucleotide identity: identical aligned bases divided by retained aligned bases
- Query coverage: retained non-overlapping query bases divided by total query length
- Reference coverage: retained non-overlapping reference bases divided by total reference length
- Conservation score: identical aligned query bases divided by total query length

External software invoked or used alongside these scripts
- BLASTN v2.14.1+ with megablast
- MOB-suite for plasmid typing/classification outputs
- Clinker v0.032 for pairwise protein-sequence alignments of translated coding sequences
- Inkscape v1.4.4 for final figure formatting
- Proksee for plasmid sequence comparison visualization

Included scripts
A. IncHI2A plasmid conservation against R478 and pSH111_227
1. contig2_reference_conservation.py (705 lines)
B. Reference-gene heatmaps and gene-level conservation visualizations
2. make_reference_gene_heatmap.py (529 lines)
3. make_ter_reference_heatmap.py (494 lines)
4. make_pco_gene_heatmap.py (215 lines)

C. pco/sil region extraction, orientation, and Clinker post-processing
5. extract_pco_sil_cluster.py (302 lines)
6. extract_reference_gene_similarity.py (236 lines)
7. annotate_clinker_similarity_on_figure.py (444 lines)
8. make_clinker_gene_labels.py (259 lines)
9. orient_pco_sil_clusters.py (527 lines)
10. clean_clinker_html_labels.py (121 lines)
11. search_cusabfcrs_genes.py (486 lines)
12. search_cus_genes_pco_sil_wide.py (148 lines)
D. MOB-suite plasmid prediction table generation
13. combine_mob_results.py (113 lines)
14. make_contig2_plasmid_type_table.py (195 lines)
15. make_contig2_plasmid_type_table_standalone.py (264 lines)
16. make_contig3_plasmid_types_colored_table.py (224 lines)
17. build_contig3_plasmid_types_word_table.py (194 lines)
E. AMR and metal-resistance summary tables/heatmaps
18. make_contig3_amr_heatmap_and_table.py (500 lines)
19. make_pndm_contig3_amr_heatmap.py (643 lines)
20. extract_card_contig3_amr_table.py (503 lines)
21. extract_card_amrfinder_contig3_combined_table.py (623 lines)
22. search_resistance_genes_in_contig3_amr_negative_isolates.py (619 lines)
23. make_mer_contig2_table.py (62 lines)
24. make_mer_contig2_table_with_references.py (261 lines)

==
==================

FULL PYTHON SOURCE CODE

==
==================

##
##################

A. IncHI2A plasmid conservation against R478 and pSH111_227

##
##################

Script 1: contig2_reference_conservation.py
Source file: contig2_reference_conservation.py
Line count: 705


```python
#!/usr/bin/env python3
"""
Calculate nucleotide conservation between NCTR contig2 plasmids and
references.
```

```
Default comparison:
  queries:    NCTR117/122/136/137/718/719/830/938 contig2 GenBank
files
  references: R478 and pSH111_227

The script extracts sequences from GenBank files, aligns each contig2
sequence
to each reference with BLASTN, then reports:
  - weighted_identity_percent: alignment-length weighted BLAST percent
identity
  - query_coverage_percent: percent of the contig2 sequence covered by
accepted HSPs
  - reference_coverage_percent: percent of the reference covered by
accepted HSPs
  - conservation_score_percent: identical aligned query bases / query
length * 100

The conservation score is useful when identity is high but only part
of the
contig aligns to a reference.
"""

from __future__ import annotations

import argparse
import csv
import html
import re
import shutil
import subprocess
import sys
from dataclasses import dataclass
from pathlib import Path


PROJECT_DIR = Path("<project_directory>")
OUT_DIR = Path("<output_directory>/contig2_conservation")
ISOLATE_ORDER = ["117", "122", "136", "137", "718", "719", "830",
"938"]

BLAST_OUTFMT = (
    "6 qseqid sseqid pident length nident mismatch gapopen "
    "qstart qend sstart send evalue bitscore qlen slen"
)


@dataclass(frozen=True)
class SequenceRecord:
    name: str
    path: Path
```

```
    fasta_path: Path
    length: int


@dataclass
class Hsp:
    query: str
    subject: str
    pident: float
    length: int
    nident: int
    mismatch: int
    gapopen: int
    qstart: int
    qend: int
    sstart: int
    send: int
    evalue: float
    bitscore: float
    qlen: int
    slen: int

    @property
    def q_interval(self) -> tuple[int, int]:
        return min(self.qstart, self.qend), max(self.qstart,
self.qend)

    @property
    def s_interval(self) -> tuple[int, int]:
        return min(self.sstart, self.send), max(self.sstart,
self.send)


@dataclass
class PairSummary:
    query: str
    reference: str
    query_length: int
    reference_length: int
    accepted_hsps: int
    aligned_bp: float
    identical_bp: float
    query_covered_bp: int
    reference_covered_bp: int
    weighted_identity_percent: float
    query_coverage_percent: float
    reference_coverage_percent: float
    conservation_score_percent: float
    raw_hsp_count: int
    query_file: Path
```

```
    reference_file: Path


def parse_args() -> argparse.Namespace:
    parser = argparse.ArgumentParser(
        description=(
            "Determine nucleotide conservation between NCTR contig2
plasmids "
            "and R478/pSH111_227 using BLASTN."
        )
    )
    parser.add_argument("--project-dir", type=Path,
default=PROJECT_DIR)
    parser.add_argument("--outdir", type=Path, default=OUT_DIR)
    parser.add_argument("--prefix",
default="contig2_vs_R478_pSH111_227_conservation")
    parser.add_argument("--blastn", default=None, help="Path to blastn
executable.")
    parser.add_argument("--min-pident", type=float, default=80.0)
    parser.add_argument("--min-length", type=int, default=500)
    parser.add_argument("--evalue", default="1e-20")
    parser.add_argument(
        "--keep-temp",
        action="store_true",
        help="Keep intermediate FASTA and raw BLAST files.",
    )
    parser.add_argument(
        "--references",
        nargs="*",
        type=Path,
        default=None,
        help=(
            "Optional explicit reference GenBank files. If omitted,
R478 and "
            "pSH111_227 are auto-detected."
        ),
    )
    parser.add_argument(
        "--queries",
        nargs="*",
        type=Path,
        default=None,
        help=(
            "Optional explicit contig2 GenBank files. If omitted,
NCTR*_contig2 "
            "files are auto-detected."
        ),
    )
    return parser.parse_args()
```

```
def first_existing(paths: list[Path], label: str) -> Path:
    for path in paths:
        if path.exists():
            return path
    raise FileNotFoundError(
        f"Could not find {label}. Tried:\n" + "\n".join(str(path) for
path in paths)
    )


def detect_references(project_dir: Path) -> list[tuple[str, Path]]:
    r478 = first_existing(
        [
            project_dir / "all_contigs" / "R478.gbk",
            project_dir / "R478.gbk",
            project_dir / "pco_sil_clusters_wide_display_names" /
"R478.gbk",
            project_dir /
"ter_clusters_all_isolates_wide_display_names" / "R478.gbk",
            project_dir / "ter_clusters_all_isolates_display_names" /
"R478.gbk",
        ],
        "R478 GenBank file",
    )
    psh111 = first_existing(
        [
            project_dir / "pSH111_227.gbk",
            project_dir / "pSH111_227.gb",
            project_dir / "all_contigs" / "pSH111_227.gb",
            project_dir / "pco_sil_clusters_wide_display_names" /
"pSH111_227.gbk",
        ],
        "pSH111_227 GenBank file",
    )
    return [("R478", r478), ("pSH111_227", psh111)]


def detect_queries(project_dir: Path) -> list[tuple[str, Path]]:
    queries: list[tuple[str, Path]] = []
    for isolate in ISOLATE_ORDER:
        sample = f"NCTR{isolate}_contig2"
        path = first_existing(
            [
                project_dir / "all_contigs" / f"{sample}.gbk",
                project_dir / f"{sample}.gbk",
                project_dir / "gbk" / "all_contigs" / f"{sample}.gbk",
                project_dir / "gbk" / f"{sample}_prokka" /
f"{sample}.gbk",
            ],
```

```
            sample,
        )
        queries.append((sample, path))
    return queries


def safe_name(name: str) -> str:
    return re.sub(r"[^A-Za-z0-9_.-]+", "_", name)


def parse_genbank_sequence(path: Path) -> tuple[str, str]:
    record_id = path.stem
    sequence_parts: list[str] = []
    in_origin = False

    for line in path.read_text(encoding="utf-8",
errors="replace").splitlines():
        if line.startswith("LOCUS"):
            parts = line.split()
            if len(parts) > 1:
                record_id = parts[1]
        elif line.startswith("ORIGIN"):
            in_origin = True
        elif in_origin:
            if line.startswith("//"):
                break
            sequence_parts.append(re.sub(r"[^A-Za-z]", "", line))

    sequence = "".join(sequence_parts).upper()
    if not sequence:
        raise ValueError(f"No ORIGIN sequence found in {path}")
    return record_id, sequence


def write_fasta(path: Path, name: str, sequence: str) -> None:
    path.parent.mkdir(parents=True, exist_ok=True)
    with path.open("w") as handle:
        handle.write(f">{name}\n")
        for index in range(0, len(sequence), 80):
            handle.write(sequence[index : index + 80] + "\n")


def prepare_records(
    named_paths: list[tuple[str, Path]], fasta_dir: Path
) -> list[SequenceRecord]:
    records: list[SequenceRecord] = []
    for name, gbk_path in named_paths:
        _, sequence = parse_genbank_sequence(gbk_path)
        fasta_path = fasta_dir / f"{safe_name(name)}.fasta"
        write_fasta(fasta_path, name, sequence)
```

```python
        records.append(
            SequenceRecord(
                name=name,
                path=gbk_path,
                fasta_path=fasta_path,
                length=len(sequence),
            )
        )
    return records


def find_blastn(user_blastn: str | None) -> str:
    candidates = []
    if user_blastn:
        candidates.append(user_blastn)
    candidates.extend(
        [
            shutil.which("blastn"),
            "/usr/local/ncbi/blast/bin/blastn",
            "/opt/homebrew/bin/blastn",
            "/usr/local/bin/blastn",
        ]
    )
    for candidate in candidates:
        if candidate and Path(candidate).exists():
            return str(candidate)
    raise FileNotFoundError("Could not find blastn. Add it to PATH or
use --blastn.")


def run_blastn(
    blastn: str,
    query: SequenceRecord,
    reference: SequenceRecord,
    raw_path: Path,
    min_pident: float,
    min_length: int,
    evalue: str,
) -> list[Hsp]:
    command = [
        blastn,
        "-task",
        "megablast",
        "-query",
        str(query.fasta_path),
        "-subject",
        str(reference.fasta_path),
        "-outfmt",
        BLAST_OUTFMT,
        "-dust",
```

```
        "no",
        "-soft_masking",
        "false",
        "-evalue",
        evalue,
    ]
    completed = subprocess.run(
        command,
        check=False,
        text=True,
        capture_output=True,
    )
    if completed.returncode != 0:
        raise RuntimeError(
            "blastn failed for "
            f"{query.name} vs {reference.name}\n"
            f"Command: {' '.join(command)}\n"
            f"STDERR:\n{completed.stderr}"
        )

    raw_path.write_text(completed.stdout)
    hsps: list[Hsp] = []
    for line in completed.stdout.splitlines():
        if not line.strip():
            continue
        fields = line.split("\t")
        if len(fields) != 15:
            raise ValueError(f"Unexpected BLAST row with {len(fields)}
fields: {line}")
        hsp = Hsp(
            query=fields[0],
            subject=fields[1],
            pident=float(fields[2]),
            length=int(fields[3]),
            nident=int(fields[4]),
            mismatch=int(fields[5]),
            gapopen=int(fields[6]),
            qstart=int(fields[7]),
            qend=int(fields[8]),
            sstart=int(fields[9]),
            send=int(fields[10]),
            evalue=float(fields[11]),
            bitscore=float(fields[12]),
            qlen=int(fields[13]),
            slen=int(fields[14]),
        )
        if hsp.pident >= min_pident and hsp.length >= min_length:
            hsps.append(hsp)
    return hsps
```

```python
def interval_length(interval: tuple[int, int]) -> int:
    start, end = interval
    return max(0, end - start + 1)


def overlap_length(a: tuple[int, int], b: tuple[int, int]) -> int:
    start = max(a[0], b[0])
    end = min(a[1], b[1])
    return max(0, end - start + 1)


def interval_union_length(intervals: list[tuple[int, int]]) -> int:
    if not intervals:
        return 0
    merged: list[list[int]] = []
    for start, end in sorted(intervals):
        if not merged or start > merged[-1][1] + 1:
            merged.append([start, end])
        else:
            merged[-1][1] = max(merged[-1][1], end)
    return sum(end - start + 1 for start, end in merged)


def new_query_bases(interval: tuple[int, int], accepted:
list[tuple[int, int]]) -> int:
    total = interval_length(interval)
    if total == 0:
        return 0
    covered = interval_union_length(
        [
            (max(interval[0], existing[0]), min(interval[1],
existing[1]))
            for existing in accepted
            if overlap_length(interval, existing) > 0
        ]
    )
    return max(0, total - covered)


def summarize_pair(
    query: SequenceRecord, reference: SequenceRecord, hsps: list[Hsp]
) -> PairSummary:
    accepted_query_intervals: list[tuple[int, int]] = []
    accepted_reference_intervals: list[tuple[int, int]] = []
    aligned_bp = 0.0
    identical_bp = 0.0
    accepted_hsps = 0

    sorted_hsps = sorted(hsps, key=lambda item: (item.bitscore,
```

```
item.length), reverse=True)
    for hsp in sorted_hsps:
        q_interval = hsp.q_interval
        new_bp = new_query_bases(q_interval, accepted_query_intervals)
        if new_bp <= 0:
            continue

        fraction_new = new_bp / max(1, interval_length(q_interval))
        aligned_bp += hsp.length * fraction_new
        identical_bp += hsp.nident * fraction_new
        accepted_query_intervals.append(q_interval)
        accepted_reference_intervals.append(hsp.s_interval)
        accepted_hsps += 1

    query_covered_bp = interval_union_length(accepted_query_intervals)
    reference_covered_bp =
interval_union_length(accepted_reference_intervals)
    weighted_identity = (identical_bp / aligned_bp * 100.0) if
aligned_bp else 0.0
    query_coverage = query_covered_bp / query.length * 100.0
    reference_coverage = reference_covered_bp / reference.length *
100.0
    conservation_score = identical_bp / query.length * 100.0

    return PairSummary(
        query=query.name,
        reference=reference.name,
        query_length=query.length,
        reference_length=reference.length,
        accepted_hsps=accepted_hsps,
        aligned_bp=aligned_bp,
        identical_bp=identical_bp,
        query_covered_bp=query_covered_bp,
        reference_covered_bp=reference_covered_bp,
        weighted_identity_percent=weighted_identity,
        query_coverage_percent=query_coverage,
        reference_coverage_percent=reference_coverage,
        conservation_score_percent=conservation_score,
        raw_hsp_count=len(hsps),
        query_file=query.path,
        reference_file=reference.path,
    )


def write_summary(path: Path, summaries: list[PairSummary]) -> None:
    fields = [
        "query",
        "reference",
        "weighted_identity_percent",
        "query_coverage_percent",
```

```
        "reference_coverage_percent",
        "conservation_score_percent",
        "aligned_bp",
        "identical_bp",
        "query_covered_bp",
        "reference_covered_bp",
        "query_length",
        "reference_length",
        "accepted_hsps",
        "raw_hsp_count",
        "query_file",
        "reference_file",
    ]
    with path.open("w", newline="") as handle:
        writer = csv.DictWriter(handle, fieldnames=fields,
delimiter="\t")
        writer.writeheader()
        for item in summaries:
            writer.writerow(
                {
                    "query": item.query,
                    "reference": item.reference,
                    "weighted_identity_percent":
f"{item.weighted_identity_percent:.4f}",
                    "query_coverage_percent":
f"{item.query_coverage_percent:.4f}",
                    "reference_coverage_percent":
f"{item.reference_coverage_percent:.4f}",
                    "conservation_score_percent":
f"{item.conservation_score_percent:.4f}",
                    "aligned_bp": f"{item.aligned_bp:.1f}",
                    "identical_bp": f"{item.identical_bp:.1f}",
                    "query_covered_bp": item.query_covered_bp,
                    "reference_covered_bp": item.reference_covered_bp,
                    "query_length": item.query_length,
                    "reference_length": item.reference_length,
                    "accepted_hsps": item.accepted_hsps,
                    "raw_hsp_count": item.raw_hsp_count,
                    "query_file": str(item.query_file),
                    "reference_file": str(item.reference_file),
                }
            )


def write_wide_matrix(path: Path, summaries: list[PairSummary],
metric: str) -> None:
    queries = list(dict.fromkeys(item.query for item in summaries))
    references = list(dict.fromkeys(item.reference for item in
summaries))
    values = {(item.query, item.reference): getattr(item, metric) for
```

```
item in summaries}
    with path.open("w", newline="") as handle:
        writer = csv.writer(handle, delimiter="\t")
        writer.writerow(["query"] + references)
        for query in queries:
            writer.writerow(
                [query] + [f"{values.get((query, reference),
0.0):.4f}" for reference in references]
            )


def write_raw_hsps(path: Path, pair_hsps: list[tuple[str, str, Hsp]])
-> None:
    fields = [
        "query",
        "reference",
        "pident",
        "length",
        "nident",
        "mismatch",
        "gapopen",
        "qstart",
        "qend",
        "sstart",
        "send",
        "evalue",
        "bitscore",
        "qlen",
        "slen",
    ]
    with path.open("w", newline="") as handle:
        writer = csv.DictWriter(handle, fieldnames=fields,
delimiter="\t")
        writer.writeheader()
        for query, reference, hsp in pair_hsps:
            writer.writerow(
                {
                    "query": query,
                    "reference": reference,
                    "pident": f"{hsp.pident:.4f}",
                    "length": hsp.length,
                    "nident": hsp.nident,
                    "mismatch": hsp.mismatch,
                    "gapopen": hsp.gapopen,
                    "qstart": hsp.qstart,
                    "qend": hsp.qend,
                    "sstart": hsp.sstart,
                    "send": hsp.send,
                    "evalue": hsp.evalue,
                    "bitscore": hsp.bitscore,
```

```
                    "qlen": hsp.qlen,
                    "slen": hsp.slen,
                }
            )


def color_for_score(score: float) -> str:
    # White to blue scale, keyed to conservation score.
    score = max(0.0, min(100.0, score))
    t = score / 100.0
    r = round(238 - 170 * t)
    g = round(244 - 116 * t)
    b = round(250 - 42 * t)
    return f"#{r:02x}{g:02x}{b:02x}"


def display_query_label(query: str) -> str:
    return query.replace("_contig2", "_IncHI2").replace(" contig 2", "
IncHI2").replace("contig2", "IncHI2")


def write_svg_heatmap(path: Path, summaries: list[PairSummary]) ->
None:
    queries = list(dict.fromkeys(item.query for item in summaries))
    references = list(dict.fromkeys(item.reference for item in
summaries))
    values = {(item.query, item.reference): item for item in
summaries}

    left = 165
    top = 105
    cell_w = 118
    cell_h = 42
    right_margin = 90
    minimum_width = 820
    width = max(left + len(references) * cell_w + right_margin,
minimum_width)
    height = top + len(queries) * cell_h + 105

    svg: list[str] = []
    svg.append(
        f'<svg xmlns="http://www.w3.org/2000/svg" width="{width}"
height="{height}" '
        f'viewBox="0 0 {width} {height}">'
    )
    svg.append('<rect width="100%" height="100%" fill="#ffffff"/>')
    svg.append(
        "<style>"
        "text{font-family:Arial,Helvetica,sans-serif}"
        ".title{font-size:18px;font-weight:700}"
```

```
        ".small{font-size:11px}"
        ".label{font-size:12px}"
        ".sample{font-size:12px}"
        ".cell{font-size:11px;font-weight:700}"
        "</style>"
    )
    svg.append(
        '<text x="24" y="32" class="title">'
        "IncHI2 Conservation Against R478 and pSH111_227"
        "</text>"
    )
    svg.append(
        '<text x="24" y="54" class="small" fill="#444">'
        "Cell color = conservation score (% identical query bases).
Text = weighted identity / query coverage."
        "</text>"
    )

    for col, reference in enumerate(references):
        x = left + col * cell_w + cell_w / 2
        svg.append(
            f'<text x="{x:.1f}" y="{top - 18}" class="label" text-
anchor="middle">'
            f"{html.escape(reference)}</text>"
        )

    for row, query in enumerate(queries):
        y = top + row * cell_h
        svg.append(
            f'<text x="{left - 10}" y="{y + 25}" class="sample" text-
anchor="end">'
            f"{html.escape(display_query_label(query))}</text>"
        )
        for col, reference in enumerate(references):
            x = left + col * cell_w
            item = values[(query, reference)]
            fill = color_for_score(item.conservation_score_percent)
            svg.append(
                f'<rect x="{x}" y="{y}" width="{cell_w - 6}"
height="{cell_h - 6}" '
                f'rx="2" fill="{fill}" stroke="#b8c1cc" stroke-
width="0.8"/>'
            )
            svg.append(
                f'<text x="{x + (cell_w - 6) / 2:.1f}" y="{y + 16}" '
                f'class="cell" text-anchor="middle" fill="#1f2933">'
                f"{item.weighted_identity_percent:.2f}% id</text>"
            )
            svg.append(
                f'<text x="{x + (cell_w - 6) / 2:.1f}" y="{y + 31}" '
```

```
                f'class="small" text-anchor="middle" fill="#374151">'
                f"{item.query_coverage_percent:.2f}% qcov</text>"
            )

    legend_y = top + len(queries) * cell_h + 42
    svg.append(f'<text x="24" y="{legend_y}" class="small"
fill="#444">Low</text>')
    for index in range(101):
        x = 58 + index
        svg.append(
            f'<rect x="{x}" y="{legend_y - 12}" width="1" height="12"
'
            f'fill="{color_for_score(index)}"/>'
        )
    svg.append(f'<text x="166" y="{legend_y}" class="small"
fill="#444">High conservation score</text>')
    svg.append("</svg>")
    path.write_text("\n".join(svg))


def remove_temp(temp_dir: Path) -> None:
    if temp_dir.exists():
        shutil.rmtree(temp_dir)


def main() -> int:
    args = parse_args()
    try:
        blastn = find_blastn(args.blastn)
        reference_paths = (
            [(path.stem, path) for path in args.references]
            if args.references
            else detect_references(args.project_dir)
        )
        query_paths = (
            [(path.stem, path) for path in args.queries]
            if args.queries
            else detect_queries(args.project_dir)
        )
    except Exception as error:
        print(f"ERROR: {error}", file=sys.stderr)
        return 2

    args.outdir.mkdir(parents=True, exist_ok=True)
    temp_dir = args.outdir / f"{args.prefix}_tmp"
    fasta_dir = temp_dir / "fasta"
    raw_dir = temp_dir / "raw_blast"
    raw_dir.mkdir(parents=True, exist_ok=True)

    try:
```

```
        query_records = prepare_records(query_paths, fasta_dir)
        reference_records = prepare_records(reference_paths,
fasta_dir)

        summaries: list[PairSummary] = []
        raw_hsps: list[tuple[str, str, Hsp]] = []
        for query in query_records:
            for reference in reference_records:
                raw_path = raw_dir / f"{safe_name(query.name)}
_vs_{safe_name(reference.name)}.blast.tsv"
                hsps = run_blastn(
                    blastn=blastn,
                    query=query,
                    reference=reference,
                    raw_path=raw_path,
                    min_pident=args.min_pident,
                    min_length=args.min_length,
                    evalue=args.evalue,
                )
                summaries.append(summarize_pair(query, reference,
hsps))
                raw_hsps.extend((query.name, reference.name, hsp) for
hsp in hsps)

        summary_path = args.outdir / f"{args.prefix}.tsv"
        identity_matrix_path = args.outdir / f"{args.prefix}
_weighted_identity_matrix.tsv"
        conservation_matrix_path = args.outdir / f"{args.prefix}
_score_matrix.tsv"
        raw_hsp_path = args.outdir / f"{args.prefix}_raw_hsps.tsv"
        svg_path = args.outdir / f"{args.prefix}_heatmap.svg"

        write_summary(summary_path, summaries)
        write_wide_matrix(identity_matrix_path, summaries,
"weighted_identity_percent")
        write_wide_matrix(conservation_matrix_path, summaries,
"conservation_score_percent")
        write_raw_hsps(raw_hsp_path, raw_hsps)
        write_svg_heatmap(svg_path, summaries)
    except Exception as error:
        print(f"ERROR: {error}", file=sys.stderr)
        return 1
    finally:
        if not args.keep_temp:
            remove_temp(temp_dir)

    print(f"blastn used: {blastn}")
    print("References:")
    for name, path in reference_paths:
        print(f"  {name}: {path}")
```

```
    print("Queries:")
    for name, path in query_paths:
        print(f"  {name}: {path}")
    print()
    print("Saved:")
    print(f"  {summary_path}")
    print(f"  {identity_matrix_path}")
    print(f"  {conservation_matrix_path}")
    print(f"  {raw_hsp_path}")
    print(f"  {svg_path}")
    return 0


if __name__ == "__main__":
    raise SystemExit(main())
```

##
##################
B. Reference-gene heatmaps and gene-level conservation visualizations
##
##################

Script 2: make_reference_gene_heatmap.py
Source file: make_reference_gene_heatmap.py
Line count: 529


```python
#!/usr/bin/env python3
"""
Create a ter-gene heatmap comparing NCTR isolates to pSH111_227 and
R478.

Inputs:
  - Clinker alignments TSV, e.g. ter_clinker_alignments_wide.tsv
  - The GenBank files used by Clinker, e.g. R478.gbk, pSH111_227.gbk,
NCTR*.gbk

Outputs:
  - <prefix>.svg: dependency-free SVG heatmap
  - <prefix>.tsv: per-isolate/per-gene status table
  - <prefix>_hits.tsv: best reference hit details
"""

from __future__ import annotations

import argparse
```

```
import csv
import re
from pathlib import Path
from xml.sax.saxutils import escape


DEFAULT_GENES = [
    "terY3",
    "terY2",
    "terX",
    "terY1",
    "terW",
    "terZ",
    "terA",
    "terB",
    "terC",
    "terD",
    "terE",
    "terF",
]

DEFAULT_REFERENCE_COLOR = "#4c78a8"

COLORS = {
    "reference_present": DEFAULT_REFERENCE_COLOR,
    "full_both": "#2166ac",
    "full_ref1": "#2a9d8f",
    "full_ref2": "#7b3294",
    "truncated": "#f4a261",
    "absent": "#d9d9d9",
}

LABELS = {
    "reference_present": "ref",
    "full_both": "both",
    "full_ref1": "ref1",
    "full_ref2": "ref2",
    "truncated": "trunc",
    "absent": "-",
}


def parse_args() -> argparse.Namespace:
    parser = argparse.ArgumentParser(
        description="Create an SVG heatmap of ter genes in NCTR
isolates compared to pSH111_227 and R478."
    )
    parser.add_argument(
        "--gbk-dir",
        type=Path,
```

```
        default=Path("."),
        help="Directory containing R478.gbk, pSH111_227.gbk, and
NCTR*.gbk. Default: current directory.",
    )
    parser.add_argument(
        "--alignments",
        type=Path,
        default=Path("ter_clinker_alignments_wide.tsv"),
        help="Clinker alignments TSV. Default:
ter_clinker_alignments_wide.tsv",
    )
    parser.add_argument(
        "--out-prefix",
        default="ter_reference_heatmap",
        help="Output prefix. Default: ter_reference_heatmap",
    )
    parser.add_argument(
        "--genes",
        default=",".join(DEFAULT_GENES),
        help="Comma-separated gene order for columns.",
    )
    parser.add_argument(
        "--references",
        default="pSH111_227,R478",
        help="Comma-separated reference names. Default:
pSH111_227,R478",
    )
    parser.add_argument(
        "--title",
        default=None,
        help="SVG title. Default is generated from the gene label and
references.",
    )
    parser.add_argument(
        "--gene-label",
        default="ter",
        help="Gene family label used in the title and x-axis. Default:
ter",
    )
    parser.add_argument(
        "--note",
        default=None,
        help="Subtitle/note to print below the SVG title.",
    )
    parser.add_argument(
        "--min-identity",
        type=float,
        default=0.70,
        help="Minimum identity to call a reference homolog present.
Default: 0.70",
```

```
    )
    parser.add_argument(
        "--min-length-ratio",
        type=float,
        default=0.90,
        help="Minimum isolate/reference CDS length ratio to call full-
length. Default: 0.90",
    )
    parser.add_argument(
        "--no-reference-rows",
        action="store_true",
        help="Do not include R478/pSH111_227 rows in the heatmap.",
    )
    parser.add_argument(
        "--reference-color",
        default=DEFAULT_REFERENCE_COLOR,
        help=f"Color for present genes in reference rows. Default:
{DEFAULT_REFERENCE_COLOR}",
    )
    parser.add_argument(
        "--no-legend",
        action="store_true",
        help="Do not draw the legend on the SVG heatmap.",
    )
    return parser.parse_args()


def parse_gbk(path: Path) -> dict[str, dict]:
    lines = path.read_text(errors="replace").splitlines()
    features = {}
    in_features = False
    current = None

    def flush() -> None:
        nonlocal current
        if not current:
            return
        nums = [int(x) for x in re.findall(r"\d+",
current["location"])]
        if not nums:
            current = None
            return
        current["start"] = min(nums)
        current["end"] = max(nums)
        current["length"] = current["end"] - current["start"] + 1
        qtext = "\n".join(current["qualifiers"])

        def qual(name: str) -> str:
            match = re.search(rf'/{name}="([^"]+)"', qtext)
            return match.group(1) if match else ""
```

```
        gene = qual("gene")
        locus_tag = qual("locus_tag")
        label = gene or locus_tag
        if label:
            features[label] = {
                "label": label,
                "gene": gene,
                "locus_tag": locus_tag,
                "product": qual("product"),
                "start": current["start"],
                "end": current["end"],
                "length": current["length"],
            }
        current = None

    for line in lines:
        if line.startswith("FEATURES"):
            in_features = True
            continue
        if line.startswith("ORIGIN"):
            flush()
            break
        if not in_features or not line.startswith("     "):
            continue
        key = line[5:21].strip()
        value = line[21:].strip()
        if key:
            flush()
            current = {"location": value, "qualifiers": []} if key ==
"CDS" else None
        elif current is not None:
            current["qualifiers"].append(value)

    return features


def read_alignments(path: Path) -> list[dict]:
    rows = []
    block = None
    for raw in path.read_text(errors="replace").splitlines():
        line = raw.strip()
        block_match = re.match(r"^(.+?)\s+vs\s+(.+)$", line)
        if block_match:
            block = (block_match.group(1).strip(),
block_match.group(2).strip())
            continue
        if not block or not line or line.startswith("-") or
line.startswith("Query"):
            continue
```

```
        parts = line.split()
        if len(parts) < 4:
            continue
        query, target, identity, similarity = parts[:4]
        try:
            identity_f = float(identity)
            similarity_f = float(similarity)
        except ValueError:
            continue
        rows.append(
            {
                "cluster_a": block[0],
                "cluster_b": block[1],
                "query": query,
                "target": target,
                "identity": identity_f,
                "similarity": similarity_f,
            }
        )
    return rows


def reference_aliases(rows: list[dict], features: dict, refs:
list[str], genes: list[str], min_identity: float) -> dict:
    aliases = {ref: {} for ref in refs}
    for ref in refs:
        for gene in genes:
            if gene in features.get(ref, {}):
                aliases[ref][gene] = gene

    if len(refs) < 2:
        return aliases

    for gene in genes:
        for ref in refs:
            if gene in aliases[ref]:
                continue
            best = None
            for other in refs:
                if other == ref:
                    continue
                other_label = aliases.get(other, {}).get(gene, gene)
                for row in rows:
                    candidate = None
                    if row["cluster_a"] == other and row["cluster_b"]
== ref and row["query"] == other_label:
                        candidate = row["target"]
                    elif row["cluster_b"] == other and
row["cluster_a"] == ref and row["target"] == other_label:
                        candidate = row["query"]
```

```
                    if candidate and row["identity"] >= min_identity:
                        if best is None or row["identity"] >
best["identity"]:
                            best = {"label": candidate, "identity":
row["identity"]}
            if best:
                aliases[ref][gene] = best["label"]

    return aliases


def best_hits(rows: list[dict], isolates: list[str], refs: list[str],
genes: list[str], aliases: dict) -> dict:
    best = {isolate: {gene: {} for gene in genes} for isolate in
isolates}
    gene_set = set(genes)

    for row in rows:
        for ref in refs:
            for isolate in isolates:
                ref_to_gene = {aliases.get(ref, {}).get(gene): gene
for gene in genes if aliases.get(ref, {}).get(gene)}
                if row["cluster_a"] == ref and row["cluster_b"] ==
isolate and row["query"] in ref_to_gene:
                    gene = ref_to_gene[row["query"]]
                    current = best[isolate][gene].get(ref)
                    if current is None or row["identity"] >
current["identity"]:
                        best[isolate][gene][ref] = {
                            "reference": ref,
                            "reference_gene": gene,
                            "reference_label": row["query"],
                            "isolate_gene": row["target"],
                            "identity": row["identity"],
                            "similarity": row["similarity"],
                        }
                elif row["cluster_b"] == ref and row["cluster_a"] ==
isolate and row["target"] in ref_to_gene:
                    gene = ref_to_gene[row["target"]]
                    current = best[isolate][gene].get(ref)
                    if current is None or row["identity"] >
current["identity"]:
                        best[isolate][gene][ref] = {
                            "reference": ref,
                            "reference_gene": gene,
                            "reference_label": row["target"],
                            "isolate_gene": row["query"],
                            "identity": row["identity"],
                            "similarity": row["similarity"],
                        }
```

```
    return best


def classify_hits(best: dict, features: dict, refs: list[str], genes: 
list[str], aliases: dict, min_identity: float, min_ratio: float) -> 
tuple[dict, list[dict]]:
    statuses = {}
    hit_rows = []

    for isolate, gene_hits in best.items():
        statuses[isolate] = {}
        for gene in genes:
            accepted = []
            truncated = []
            for ref in refs:
                hit = gene_hits[gene].get(ref)
                if hit is None or hit["identity"] < min_identity:
                    continue
                reference_label = aliases.get(ref, {}).get(gene, gene)
                ref_feature = features.get(ref, 
{}).get(reference_label)
                iso_feature = features.get(isolate, 
{}).get(hit["isolate_gene"])
                ratio = None
                if ref_feature and iso_feature:
                    ratio = iso_feature["length"] / 
ref_feature["length"]
                    if ratio >= min_ratio:
                        accepted.append(ref)
                    else:
                        truncated.append(ref)
                else:
                    accepted.append(ref)

                hit_rows.append(
                    {
                        "isolate": isolate,
                        "gene": gene,
                        "reference": ref,
                        "reference_label": reference_label,
                        "isolate_gene": hit["isolate_gene"],
                        "identity": f'{hit["identity"]:.3f}',
                        "similarity": f'{hit["similarity"]:.3f}',
                        "reference_length": ref_feature["length"] if 
ref_feature else "",
                        "isolate_length": iso_feature["length"] if 
iso_feature else "",
                        "length_ratio": f"{ratio:.3f}" if ratio is not 
None else "",
```

```
                }
            )

        if len(accepted) == len(refs):
            status = "full_both"
        elif accepted == [refs[0]]:
            status = "full_ref1"
        elif len(refs) > 1 and accepted == [refs[1]]:
            status = "full_ref2"
        elif accepted:
            status = "full_both"
        elif truncated:
            status = "truncated"
        else:
            status = "absent"
        statuses[isolate][gene] = status

    return statuses, hit_rows


def write_status_tsv(path: Path, statuses: dict, genes: list[str]) ->
None:
    with path.open("w", newline="") as handle:
        writer = csv.writer(handle, delimiter="\t")
        writer.writerow(["Isolate", *genes])
        for isolate, gene_statuses in statuses.items():
            writer.writerow([isolate, *[gene_statuses[gene] for gene
in genes]])


def add_reference_rows(statuses: dict, features: dict, refs:
list[str], genes: list[str], aliases: dict) -> dict:
    combined = {}
    for ref in refs:
        combined[ref] = {}
        for gene in genes:
            label = aliases.get(ref, {}).get(gene, gene)
            combined[ref][gene] = "reference_present" if label in
features.get(ref, {}) else "absent"
    combined.update(statuses)
    return combined


def write_hits_tsv(path: Path, rows: list[dict]) -> None:
    fields = [
        "isolate",
        "gene",
        "reference",
        "reference_label",
        "isolate_gene",
```

```
        "identity",
        "similarity",
        "reference_length",
        "isolate_length",
        "length_ratio",
    ]
    with path.open("w", newline="") as handle:
        writer = csv.DictWriter(handle, delimiter="\t",
fieldnames=fields)
        writer.writeheader()
        writer.writerows(rows)


def svg_text(x, y, value, **attrs) -> str:
    attr = " ".join(f'{k.replace("_", "-")}="{escape(str(v))}"' for k,
v in attrs.items())
    return f'<text x="{x}" y="{y}" {attr}>{escape(str(value))}</text>'


def svg_rect(x, y, width, height, **attrs) -> str:
    attr = " ".join(f'{k.replace("_", "-")}="{escape(str(v))}"' for k,
v in attrs.items())
    return f'<rect x="{x}" y="{y}" width="{width}" height="{height}"
{attr}/>'


def write_svg(
    path: Path,
    statuses: dict,
    genes: list[str],
    refs: list[str],
    title: str,
    gene_label: str,
    note: str,
    show_legend: bool = True,
) -> None:
    cell_w = 62
    cell_h = 34
    left = 105
    top = 105
    right = 330
    bottom = 76
    width = left + len(genes) * cell_w + right
    height = top + len(statuses) * cell_h + bottom

    svg = [
        '<?xml version="1.0" encoding="UTF-8"?>',
        f'<svg xmlns="http://www.w3.org/2000/svg" width="{width}"
height="{height}" viewBox="0 0 {width} {height}">',
        svg_rect(0, 0, width, height, fill="white"),
```

```
        svg_text(20, 30, title, font_family="Arial", font_size="18",
font_weight="bold"),
        svg_text(20, 54, note, font_family="Arial", font_size="12",
fill="#333"),
    ]

    for j, gene in enumerate(genes):
        x = left + j * cell_w + cell_w / 2
        y = top - 18
        svg.append(svg_text(x, y, gene, font_family="Arial",
font_size="12", text_anchor="middle", transform=f"rotate(-45 {x}
{y})"))

    for i, (isolate, gene_statuses) in enumerate(statuses.items()):
        y = top + i * cell_h
        svg.append(svg_text(left - 12, y + cell_h / 2 + 4, isolate,
font_family="Arial", font_size="12", text_anchor="end"))
        for j, gene in enumerate(genes):
            x = left + j * cell_w
            status = gene_statuses[gene]
            svg.append(svg_rect(x, y, cell_w, cell_h,
fill=COLORS[status], stroke="white", stroke_width="2"))
            svg.append(svg_text(x + cell_w / 2, y + cell_h / 2 + 4,
LABELS[status], font_family="Arial", font_size="10",
text_anchor="middle", fill="black"))

    svg.append(svg_text(left + len(genes) * cell_w / 2, height - 26,
f"{gene_label} genes", font_family="Arial", font_size="13",
text_anchor="middle"))
    axis_y = top + len(statuses) * cell_h / 2
    svg.append(svg_text(22, axis_y, "NCTR isolates",
font_family="Arial", font_size="13", text_anchor="middle",
transform=f"rotate(-90 22 {axis_y})"))

    if show_legend:
        legend_x = left + len(genes) * cell_w + 40
        legend_y = top
        present_statuses = {
            gene_statuses[gene]
            for gene_statuses in statuses.values()
            for gene in genes
        }
        legend = [
            ("reference_present", "Reference gene/homolog present"),
            ("full_both", "Full-length homolog vs both references"),
            ("full_ref1", f"Full-length homolog vs {refs[0]} only"),
            ("full_ref2", f"Full-length homolog vs {refs[1]} only" if
len(refs) > 1 else "Full-length homolog vs alternate reference"),
            ("truncated", "Candidate truncation / short CDS"),
            ("absent", "Absent / no confident hit"),
```

```
        ]
        legend = [(status, label) for status, label in legend if
status in present_statuses]
        svg.append(svg_text(legend_x, legend_y - 20, "Legend",
font_family="Arial", font_size="13", font_weight="bold"))
        for k, (status, label) in enumerate(legend):
            y = legend_y + k * 30
            svg.append(svg_rect(legend_x, y, 18, 18,
fill=COLORS[status], stroke="white"))
            svg.append(svg_text(legend_x + 28, y + 14, label,
font_family="Arial", font_size="12"))

    svg.append("</svg>")
    path.write_text("\n".join(svg) + "\n", encoding="utf-8")


def main() -> None:
    args = parse_args()
    COLORS["reference_present"] = args.reference_color
    gbk_dir = args.gbk_dir
    alignments = args.alignments
    if not alignments.is_absolute():
        alignments = gbk_dir / alignments

    genes = [x.strip() for x in args.genes.split(",") if x.strip()]
    refs = [x.strip() for x in args.references.split(",") if
x.strip()]

    gbk_files = sorted(gbk_dir.glob("*.gbk"))
    clusters = [path.stem for path in gbk_files]
    isolates = [name for name in clusters if name.startswith("NCTR")]

    if not isolates:
        raise SystemExit(f"No NCTR*.gbk files found in {gbk_dir}")
    for ref in refs:
        if ref not in clusters:
            raise SystemExit(f"Reference {ref}.gbk not found in
{gbk_dir}")

    features = {path.stem: parse_gbk(path) for path in gbk_files}
    alignment_rows = read_alignments(alignments)
    aliases = reference_aliases(alignment_rows, features, refs, genes,
args.min_identity)
    best = best_hits(alignment_rows, isolates, refs, genes, aliases)
    statuses, hit_rows = classify_hits(best, features, refs, genes,
aliases, args.min_identity, args.min_length_ratio)
    plot_statuses = statuses if args.no_reference_rows else
add_reference_rows(statuses, features, refs, genes, aliases)

    out_prefix = Path(args.out_prefix)
```

```
    if not out_prefix.is_absolute():
        out_prefix = gbk_dir / out_prefix

    title = args.title or f"{args.gene_label} gene homologs in NCTR isolates compared with {' and '.join(refs)}"
    note = args.note or "Color indicates the best homolog call from Clinker alignments; orange indicates candidate truncation by CDS length."

    write_status_tsv(out_prefix.with_suffix(".tsv"), plot_statuses, genes)
    write_hits_tsv(out_prefix.with_name(out_prefix.name + "_hits.tsv"), hit_rows)
    write_svg(
        out_prefix.with_suffix(".svg"),
        plot_statuses,
        genes,
        refs,
        title=title,
        gene_label=args.gene_label,
        note=note,
        show_legend=not args.no_legend,
    )

    print(f"Wrote {out_prefix.with_suffix('.svg')}")
    print(f"Wrote {out_prefix.with_suffix('.tsv')}")
    print(f"Wrote {out_prefix.with_name(out_prefix.name + '_hits.tsv')}")


if __name__ == "__main__":
    main()
```

Script 3: make_ter_reference_heatmap.py
Source file: make_ter_reference_heatmap.py
Line count: 494

```python
#!/usr/bin/env python3
"""
Create a ter-gene heatmap comparing NCTR isolates to pSH111_227 and R478.

Inputs:
  - Clinker alignments TSV, e.g. ter_clinker_alignments_wide.tsv
  - The GenBank files used by Clinker, e.g. R478.gbk, pSH111_227.gbk,
```

```
NCTR*.gbk

Outputs:
  - <prefix>.svg: dependency-free SVG heatmap
  - <prefix>.tsv: per-isolate/per-gene status table
  - <prefix>_hits.tsv: best reference hit details
"""

from __future__ import annotations

import argparse
import csv
import re
from pathlib import Path
from xml.sax.saxutils import escape


DEFAULT_GENES = [
    "terY3",
    "terY2",
    "terX",
    "terY1",
    "terW",
    "terZ",
    "terA",
    "terB",
    "terC",
    "terD",
    "terE",
    "terF",
]

DEFAULT_REFERENCE_COLOR = "#4c78a8"

COLORS = {
    "reference_present": DEFAULT_REFERENCE_COLOR,
    "full_both": "#2166ac",
    "full_psh111": "#2a9d8f",
    "full_r478": "#7b3294",
    "truncated": "#f4a261",
    "absent": "#d9d9d9",
}

LABELS = {
    "reference_present": "ref",
    "full_both": "both",
    "full_psh111": "pSH",
    "full_r478": "R478",
    "truncated": "trunc",
    "absent": "-",
```

```
}


def parse_args() -> argparse.Namespace:
    parser = argparse.ArgumentParser(
        description="Create an SVG heatmap of ter genes in NCTR 
isolates compared to pSH111_227 and R478."
    )
    parser.add_argument(
        "--gbk-dir",
        type=Path,
        default=Path("."),
        help="Directory containing R478.gbk, pSH111_227.gbk, and 
NCTR*.gbk. Default: current directory.",
    )
    parser.add_argument(
        "--alignments",
        type=Path,
        default=Path("ter_clinker_alignments_wide.tsv"),
        help="Clinker alignments TSV. Default: 
ter_clinker_alignments_wide.tsv",
    )
    parser.add_argument(
        "--out-prefix",
        default="ter_reference_heatmap",
        help="Output prefix. Default: ter_reference_heatmap",
    )
    parser.add_argument(
        "--genes",
        default=",".join(DEFAULT_GENES),
        help="Comma-separated gene order for columns.",
    )
    parser.add_argument(
        "--references",
        default="pSH111_227,R478",
        help="Comma-separated reference names. Default: 
pSH111_227,R478",
    )
    parser.add_argument(
        "--min-identity",
        type=float,
        default=0.70,
        help="Minimum identity to call a reference homolog present. 
Default: 0.70",
    )
    parser.add_argument(
        "--min-length-ratio",
        type=float,
        default=0.90,
        help="Minimum isolate/reference CDS length ratio to call full-
```

```
length. Default: 0.90",
    )
    parser.add_argument(
        "--no-reference-rows",
        action="store_true",
        help="Do not include R478/pSH111_227 rows in the heatmap.",
    )
    parser.add_argument(
        "--reference-color",
        default=DEFAULT_REFERENCE_COLOR,
        help=f"Color for present genes in reference rows. Default:
{DEFAULT_REFERENCE_COLOR}",
    )
    parser.add_argument(
        "--no-legend",
        action="store_true",
        help="Do not draw the legend on the SVG heatmap.",
    )
    return parser.parse_args()


def parse_gbk(path: Path) -> dict[str, dict]:
    lines = path.read_text(errors="replace").splitlines()
    features = {}
    in_features = False
    current = None

    def flush() -> None:
        nonlocal current
        if not current:
            return
        nums = [int(x) for x in re.findall(r"\d+",
current["location"])]
        if not nums:
            current = None
            return
        current["start"] = min(nums)
        current["end"] = max(nums)
        current["length"] = current["end"] - current["start"] + 1
        qtext = "\n".join(current["qualifiers"])

        def qual(name: str) -> str:
            match = re.search(rf'/{name}="([^"]+)"', qtext)
            return match.group(1) if match else ""

        gene = qual("gene")
        locus_tag = qual("locus_tag")
        label = gene or locus_tag
        if label:
            features[label] = {
```

```
                "label": label,
                "gene": gene,
                "locus_tag": locus_tag,
                "product": qual("product"),
                "start": current["start"],
                "end": current["end"],
                "length": current["length"],
            }
        current = None

    for line in lines:
        if line.startswith("FEATURES"):
            in_features = True
            continue
        if line.startswith("ORIGIN"):
            flush()
            break
        if not in_features or not line.startswith("     "):
            continue
        key = line[5:21].strip()
        value = line[21:].strip()
        if key:
            flush()
            current = {"location": value, "qualifiers": []} if key ==
"CDS" else None
        elif current is not None:
            current["qualifiers"].append(value)

    return features


def read_alignments(path: Path) -> list[dict]:
    rows = []
    block = None
    for raw in path.read_text(errors="replace").splitlines():
        line = raw.strip()
        block_match = re.match(r"^(.+?)\s+vs\s+(.+)$", line)
        if block_match:
            block = (block_match.group(1).strip(),
block_match.group(2).strip())
            continue
        if not block or not line or line.startswith("-") or
line.startswith("Query"):
            continue
        parts = line.split()
        if len(parts) < 4:
            continue
        query, target, identity, similarity = parts[:4]
        try:
            identity_f = float(identity)
```

```
            similarity_f = float(similarity)
        except ValueError:
            continue
        rows.append(
            {
                "cluster_a": block[0],
                "cluster_b": block[1],
                "query": query,
                "target": target,
                "identity": identity_f,
                "similarity": similarity_f,
            }
        )
    return rows


def reference_aliases(rows: list[dict], features: dict, refs:
list[str], genes: list[str], min_identity: float) -> dict:
    aliases = {ref: {} for ref in refs}
    for ref in refs:
        for gene in genes:
            if gene in features.get(ref, {}):
                aliases[ref][gene] = gene

    if len(refs) < 2:
        return aliases

    for gene in genes:
        for ref in refs:
            if gene in aliases[ref]:
                continue
            best = None
            for other in refs:
                if other == ref:
                    continue
                other_label = aliases.get(other, {}).get(gene, gene)
                for row in rows:
                    candidate = None
                    if row["cluster_a"] == other and row["cluster_b"]
== ref and row["query"] == other_label:
                        candidate = row["target"]
                    elif row["cluster_b"] == other and
row["cluster_a"] == ref and row["target"] == other_label:
                        candidate = row["query"]
                    if candidate and row["identity"] >= min_identity:
                        if best is None or row["identity"] >
best["identity"]:
                            best = {"label": candidate, "identity":
row["identity"]}
            if best:
```

```
                aliases[ref][gene] = best["label"]

    return aliases


def best_hits(rows: list[dict], isolates: list[str], refs: list[str],
genes: list[str], aliases: dict) -> dict:
    best = {isolate: {gene: {} for gene in genes} for isolate in
isolates}
    gene_set = set(genes)

    for row in rows:
        for ref in refs:
            for isolate in isolates:
                ref_to_gene = {aliases.get(ref, {}).get(gene): gene
for gene in genes if aliases.get(ref, {}).get(gene)}
                if row["cluster_a"] == ref and row["cluster_b"] ==
isolate and row["query"] in ref_to_gene:
                    gene = ref_to_gene[row["query"]]
                    current = best[isolate][gene].get(ref)
                    if current is None or row["identity"] >
current["identity"]:
                        best[isolate][gene][ref] = {
                            "reference": ref,
                            "reference_gene": gene,
                            "reference_label": row["query"],
                            "isolate_gene": row["target"],
                            "identity": row["identity"],
                            "similarity": row["similarity"],
                        }
                elif row["cluster_b"] == ref and row["cluster_a"] ==
isolate and row["target"] in ref_to_gene:
                    gene = ref_to_gene[row["target"]]
                    current = best[isolate][gene].get(ref)
                    if current is None or row["identity"] >
current["identity"]:
                        best[isolate][gene][ref] = {
                            "reference": ref,
                            "reference_gene": gene,
                            "reference_label": row["target"],
                            "isolate_gene": row["query"],
                            "identity": row["identity"],
                            "similarity": row["similarity"],
                        }

    return best


def classify_hits(best: dict, features: dict, refs: list[str], genes:
list[str], aliases: dict, min_identity: float, min_ratio: float) ->
```

```
tuple[dict, list[dict]]:
    statuses = {}
    hit_rows = []

    for isolate, gene_hits in best.items():
        statuses[isolate] = {}
        for gene in genes:
            accepted = []
            truncated = []
            for ref in refs:
                hit = gene_hits[gene].get(ref)
                if hit is None or hit["identity"] < min_identity:
                    continue
                reference_label = aliases.get(ref, {}).get(gene, gene)
                ref_feature = features.get(ref,
{}).get(reference_label)
                iso_feature = features.get(isolate,
{}).get(hit["isolate_gene"])
                ratio = None
                if ref_feature and iso_feature:
                    ratio = iso_feature["length"] /
ref_feature["length"]
                    if ratio >= min_ratio:
                        accepted.append(ref)
                    else:
                        truncated.append(ref)
                else:
                    accepted.append(ref)

                hit_rows.append(
                    {
                        "isolate": isolate,
                        "gene": gene,
                        "reference": ref,
                        "reference_label": reference_label,
                        "isolate_gene": hit["isolate_gene"],
                        "identity": f'{hit["identity"]:.3f}',
                        "similarity": f'{hit["similarity"]:.3f}',
                        "reference_length": ref_feature["length"] if
ref_feature else "",
                        "isolate_length": iso_feature["length"] if
iso_feature else "",
                        "length_ratio": f"{ratio:.3f}" if ratio is not
None else "",
                    }
                )

            if len(accepted) == len(refs):
                status = "full_both"
            elif accepted == [refs[0]]:
```

```
                status = "full_psh111"
            elif len(refs) > 1 and accepted == [refs[1]]:
                status = "full_r478"
            elif accepted:
                status = "full_both"
            elif truncated:
                status = "truncated"
            else:
                status = "absent"
            statuses[isolate][gene] = status

    return statuses, hit_rows


def write_status_tsv(path: Path, statuses: dict, genes: list[str]) ->
None:
    with path.open("w", newline="") as handle:
        writer = csv.writer(handle, delimiter="\t")
        writer.writerow(["Isolate", *genes])
        for isolate, gene_statuses in statuses.items():
            writer.writerow([isolate, *[gene_statuses[gene] for gene
in genes]])


def add_reference_rows(statuses: dict, features: dict, refs:
list[str], genes: list[str], aliases: dict) -> dict:
    combined = {}
    for ref in refs:
        combined[ref] = {}
        for gene in genes:
            label = aliases.get(ref, {}).get(gene, gene)
            combined[ref][gene] = "reference_present" if label in
features.get(ref, {}) else "absent"
    combined.update(statuses)
    return combined


def write_hits_tsv(path: Path, rows: list[dict]) -> None:
    fields = [
        "isolate",
        "gene",
        "reference",
        "reference_label",
        "isolate_gene",
        "identity",
        "similarity",
        "reference_length",
        "isolate_length",
        "length_ratio",
    ]
```

```
    with path.open("w", newline="") as handle:
        writer = csv.DictWriter(handle, delimiter="\t",
fieldnames=fields)
        writer.writeheader()
        writer.writerows(rows)


def svg_text(x, y, value, **attrs) -> str:
    attr = " ".join(f'{k.replace("_", "-")}="{escape(str(v))}"' for k,
v in attrs.items())
    return f'<text x="{x}" y="{y}" {attr}>{escape(str(value))}</text>'


def svg_rect(x, y, width, height, **attrs) -> str:
    attr = " ".join(f'{k.replace("_", "-")}="{escape(str(v))}"' for k,
v in attrs.items())
    return f'<rect x="{x}" y="{y}" width="{width}" height="{height}"
{attr}/>'


def write_svg(path: Path, statuses: dict, genes: list[str], refs:
list[str], show_legend: bool = True) -> None:
    cell_w = 62
    cell_h = 34
    left = 105
    top = 105
    right = 330
    bottom = 76
    width = left + len(genes) * cell_w + right
    height = top + len(statuses) * cell_h + bottom

    title = f"ter gene homologs in NCTR isolates compared with {' and
'.join(refs)}"
    svg = [
        '<?xml version="1.0" encoding="UTF-8"?>',
        f'<svg xmlns="http://www.w3.org/2000/svg" width="{width}"
height="{height}" viewBox="0 0 {width} {height}">',
        svg_rect(0, 0, width, height, fill="white"),
        svg_text(20, 30, title, font_family="Arial", font_size="18",
font_weight="bold"),
        svg_text(20, 54, "Color indicates the best homolog call from
Clinker alignments; orange indicates candidate truncation by CDS
length.", font_family="Arial", font_size="12", fill="#333"),
    ]

    for j, gene in enumerate(genes):
        x = left + j * cell_w + cell_w / 2
        y = top - 18
        svg.append(svg_text(x, y, gene, font_family="Arial",
font_size="12", text_anchor="middle", transform=f"rotate(-45 {x}
```

```
{y})"))

    for i, (isolate, gene_statuses) in enumerate(statuses.items()):
        y = top + i * cell_h
        svg.append(svg_text(left - 12, y + cell_h / 2 + 4, isolate,
font_family="Arial", font_size="12", text_anchor="end"))
        for j, gene in enumerate(genes):
            x = left + j * cell_w
            status = gene_statuses[gene]
            svg.append(svg_rect(x, y, cell_w, cell_h,
fill=COLORS[status], stroke="white", stroke_width="2"))
            svg.append(svg_text(x + cell_w / 2, y + cell_h / 2 + 4,
LABELS[status], font_family="Arial", font_size="10",
text_anchor="middle", fill="black"))

    svg.append(svg_text(left + len(genes) * cell_w / 2, height - 26,
"ter genes", font_family="Arial", font_size="13",
text_anchor="middle"))
    axis_y = top + len(statuses) * cell_h / 2
    svg.append(svg_text(22, axis_y, "NCTR isolates",
font_family="Arial", font_size="13", text_anchor="middle",
transform=f"rotate(-90 22 {axis_y})"))

    if show_legend:
        legend_x = left + len(genes) * cell_w + 40
        legend_y = top
        present_statuses = {
            gene_statuses[gene]
            for gene_statuses in statuses.values()
            for gene in genes
        }
        legend = [
            ("reference_present", "Reference gene/homolog present"),
            ("full_both", "Full-length homolog vs both references"),
            ("full_psh111", f"Full-length homolog vs {refs[0]} only"),
            ("full_r478", f"Full-length homolog vs {refs[1]} only" if
len(refs) > 1 else "Full-length homolog vs alternate reference"),
            ("truncated", "Candidate truncation / short CDS"),
            ("absent", "Absent / no confident hit"),
        ]
        legend = [(status, label) for status, label in legend if
status in present_statuses]
        svg.append(svg_text(legend_x, legend_y - 20, "Legend",
font_family="Arial", font_size="13", font_weight="bold"))
        for k, (status, label) in enumerate(legend):
            y = legend_y + k * 30
            svg.append(svg_rect(legend_x, y, 18, 18,
fill=COLORS[status], stroke="white"))
            svg.append(svg_text(legend_x + 28, y + 14, label,
font_family="Arial", font_size="12"))
```

```
    svg.append("</svg>")
    path.write_text("\n".join(svg) + "\n", encoding="utf-8")


def main() -> None:
    args = parse_args()
    COLORS["reference_present"] = args.reference_color
    gbk_dir = args.gbk_dir
    alignments = args.alignments
    if not alignments.is_absolute():
        alignments = gbk_dir / alignments

    genes = [x.strip() for x in args.genes.split(",") if x.strip()]
    refs = [x.strip() for x in args.references.split(",") if
x.strip()]

    gbk_files = sorted(gbk_dir.glob("*.gbk"))
    clusters = [path.stem for path in gbk_files]
    isolates = [name for name in clusters if name.startswith("NCTR")]

    if not isolates:
        raise SystemExit(f"No NCTR*.gbk files found in {gbk_dir}")
    for ref in refs:
        if ref not in clusters:
            raise SystemExit(f"Reference {ref}.gbk not found in
{gbk_dir}")

    features = {path.stem: parse_gbk(path) for path in gbk_files}
    alignment_rows = read_alignments(alignments)
    aliases = reference_aliases(alignment_rows, features, refs, genes,
args.min_identity)
    best = best_hits(alignment_rows, isolates, refs, genes, aliases)
    statuses, hit_rows = classify_hits(best, features, refs, genes,
aliases, args.min_identity, args.min_length_ratio)
    plot_statuses = statuses if args.no_reference_rows else
add_reference_rows(statuses, features, refs, genes, aliases)

    out_prefix = Path(args.out_prefix)
    if not out_prefix.is_absolute():
        out_prefix = gbk_dir / out_prefix

    write_status_tsv(out_prefix.with_suffix(".tsv"), plot_statuses,
genes)
    write_hits_tsv(out_prefix.with_name(out_prefix.name +
"_hits.tsv"), hit_rows)
    write_svg(out_prefix.with_suffix(".svg"), plot_statuses, genes,
refs, show_legend=not args.no_legend)

    print(f"Wrote {out_prefix.with_suffix('.svg')}")
```

```python
    print(f"Wrote {out_prefix.with_suffix('.tsv')}")
    print(f"Wrote {out_prefix.with_name(out_prefix.name +
'_hits.tsv')}")


if __name__ == "__main__":
    main()
```


Script 4: make_pco_gene_heatmap.py
Source file: make_pco_gene_heatmap.py
Line count: 215


```python
#!/usr/bin/env python3
"""
Create a pco/sil-gene presence heatmap from GenBank annotations.

This is annotation/alias based rather than strict reference-homology
based. It
counts common NCTR aliases such as copA/copD/copR as pcoA/pcoD/pcoR
and
cus/sib labels as sil-system homologs.
"""

from __future__ import annotations

import argparse
import csv
import re
from pathlib import Path
from xml.sax.saxutils import escape


GENE_ALIASES = {
    "silE": {"silE", "pcoE"},
    "silS": {"silS", "cusS", "cusS_1", "cusS_2"},
    "silR": {"silR", "cusR"},
    "silC": {"silC", "cusC"},
    "silF": {"silF", "cusF"},
    "silB": {"silB", "sibB"},
    "silA": {"silA", "cusA"},
    "silP": {"silP", "silP_1", "silP_2"},
    "pcoE": {"pcoE", "silE", "copE1", "copE2"},
    "pcoA": {"pcoA", "copA", "copA_1", "copA_2"},
    "pcoB": {"pcoB", "copB"},
    "pcoC": {"pcoC", "copC"},
```

```
    "pcoD": {"pcoD", "copD"},
    "pcoR": {"pcoR", "copR"},
    "pcoS": {"pcoS", "copS"},
    "pcoE_2": {"pcoE_2", "pcoE", "copE1", "copE2"},
}

DEFAULT_GENES = [
    "silE",
    "silS",
    "silR",
    "silC",
    "silF",
    "silB",
    "silA",
    "silP",
    "pcoE",
    "pcoA",
    "pcoB",
    "pcoC",
    "pcoD",
    "pcoR",
    "pcoS",
    "pcoE_2",
]

COLORS = {
    "present": "#e76f51",
    "absent": "#d9d9d9",
    "reference": "#8e6bbf",
}

LABELS = {
    "present": "+",
    "absent": "-",
    "reference": "ref",
}


def parse_args() -> argparse.Namespace:
    parser = argparse.ArgumentParser(description="Create a pco/sil-
gene presence SVG heatmap from GenBank files.")
    parser.add_argument(
        "--gbk-dir",
        type=Path,
        default=Path("<project_directory>/
pco_sil_clusters_display_names"),
        help="Directory containing NCTR*.gbk and reference .gbk
files.",
    )
    parser.add_argument(
```

```
        "--references",
        default="pSH111_227,R478",
        help="Comma-separated reference rows to include. Default:
pSH111_227,R478",
    )
    parser.add_argument(
        "--out-prefix",
        default="pco_sil_gene_presence_heatmap",
        help="Output prefix. Default: pco_sil_gene_presence_heatmap",
    )
    parser.add_argument(
        "--genes",
        default=",".join(DEFAULT_GENES),
        help="Comma-separated genes/columns to plot.",
    )
    parser.add_argument("--no-legend", action="store_true", help="Do
not draw a legend.")
    return parser.parse_args()


def parse_gene_names(path: Path) -> set[str]:
    names = set()
    text = path.read_text(errors="replace")
    for key in ("gene", "locus_tag"):
        for match in re.finditer(rf'/{key}="([^"]+)"', text):
            names.add(match.group(1))
    return names


def status_for_gene(names: set[str], gene: str, is_reference: bool) ->
str:
    aliases = GENE_ALIASES[gene]
    if names & aliases:
        return "reference" if is_reference else "present"
    return "absent"


def build_statuses(gbk_dir: Path, references: list[str], genes:
list[str]) -> dict[str, dict[str, str]]:
    gbk_files = {path.stem: path for path in
sorted(gbk_dir.glob("*.gbk"))}
    rows = []
    for ref in references:
        if ref in gbk_files:
            rows.append(ref)
    rows.extend(name for name in gbk_files if name.startswith("NCTR"))

    statuses = {}
    for row in rows:
        names = parse_gene_names(gbk_files[row])
```

```
        statuses[row] = {
            gene: status_for_gene(names, gene, row in references)
            for gene in genes
        }
    return statuses


def write_tsv(path: Path, statuses: dict[str, dict[str, str]], genes: list[str]) -> None:
    with path.open("w", newline="") as handle:
        writer = csv.writer(handle, delimiter="\t")
        writer.writerow(["Isolate", *genes])
        for isolate, row in statuses.items():
            writer.writerow([isolate, *[row[gene] for gene in genes]])


def svg_text(x, y, value, **attrs) -> str:
    attr = " ".join(f'{k.replace("_", "-")}="{escape(str(v))}"' for k, v in attrs.items())
    return f'<text x="{x}" y="{y}" {attr}>{escape(str(value))}</text>'


def svg_rect(x, y, width, height, **attrs) -> str:
    attr = " ".join(f'{k.replace("_", "-")}="{escape(str(v))}"' for k, v in attrs.items())
    return f'<rect x="{x}" y="{y}" width="{width}" height="{height}" {attr}/>'


def write_svg(path: Path, statuses: dict[str, dict[str, str]], genes: list[str], show_legend: bool) -> None:
    cell_w = 66
    cell_h = 34
    left = 105
    top = 92
    right = 250 if show_legend else 40
    bottom = 65
    width = left + len(genes) * cell_w + right
    height = top + len(statuses) * cell_h + bottom

    svg = [
        '<?xml version="1.0" encoding="UTF-8"?>',
        f'<svg xmlns="http://www.w3.org/2000/svg" width="{width}" height="{height}" viewBox="0 0 {width} {height}">',
        svg_rect(0, 0, width, height, fill="white"),
        svg_text(20, 30, "pco/sil genes in NCTR isolates", font_family="Arial", font_size="18", font_weight="bold"),
        svg_text(20, 54, "Aliases counted: pco/cop labels and cus/sib labels where appropriate.", font_family="Arial", font_size="12", fill="#333"),
```

```
    ]

    for j, gene in enumerate(genes):
        x = left + j * cell_w + cell_w / 2
        y = top - 15
        svg.append(svg_text(x, y, gene, font_family="Arial",
font_size="12", text_anchor="middle", transform=f"rotate(-45 {x}
{y})"))

    for i, (isolate, row) in enumerate(statuses.items()):
        y = top + i * cell_h
        svg.append(svg_text(left - 12, y + cell_h / 2 + 4, isolate,
font_family="Arial", font_size="12", text_anchor="end"))
        for j, gene in enumerate(genes):
            x = left + j * cell_w
            status = row[gene]
            svg.append(svg_rect(x, y, cell_w, cell_h,
fill=COLORS[status], stroke="white", stroke_width="2"))
            svg.append(svg_text(x + cell_w / 2, y + cell_h / 2 + 4,
LABELS[status], font_family="Arial", font_size="11",
text_anchor="middle", fill="black"))

    svg.append(svg_text(left + len(genes) * cell_w / 2, height - 24,
"pco/sil genes", font_family="Arial", font_size="13",
text_anchor="middle"))

    if show_legend:
        legend_x = left + len(genes) * cell_w + 35
        legend_y = top
        legend = [("reference", "Reference present"), ("present",
"NCTR present"), ("absent", "Absent")]
        svg.append(svg_text(legend_x, legend_y - 18, "Legend",
font_family="Arial", font_size="13", font_weight="bold"))
        for k, (status, label) in enumerate(legend):
            y = legend_y + k * 30
            svg.append(svg_rect(legend_x, y, 18, 18,
fill=COLORS[status], stroke="white"))
            svg.append(svg_text(legend_x + 28, y + 14, label,
font_family="Arial", font_size="12"))

    svg.append("</svg>")
    path.write_text("\n".join(svg) + "\n", encoding="utf-8")


def main() -> None:
    args = parse_args()
    references = [x.strip() for x in args.references.split(",") if
x.strip()]
    genes = [x.strip() for x in args.genes.split(",") if x.strip()]
    unknown = [gene for gene in genes if gene not in GENE_ALIASES]
```

```
    if unknown:
        raise SystemExit(f"Unknown gene(s): {', '.join(unknown)}")
    statuses = build_statuses(args.gbk_dir, references, genes)
    out_prefix = Path(args.out_prefix)
    if not out_prefix.is_absolute():
        out_prefix = args.gbk_dir / out_prefix
    write_tsv(out_prefix.with_suffix(".tsv"), statuses, genes)
    write_svg(out_prefix.with_suffix(".svg"), statuses, genes,
show_legend=not args.no_legend)
    print(f"Wrote {out_prefix.with_suffix('.svg')}")
    print(f"Wrote {out_prefix.with_suffix('.tsv')}")


if __name__ == "__main__":
    main()
```

##
##################
C. pco/sil region extraction, orientation, and Clinker post-processing
##
##################

Script 5: extract_pco_sil_cluster.py
Source file: extract_pco_sil_cluster.py
Line count: 302


```python
#!/usr/bin/env python3
"""
Extract pco/sil gene-cluster regions from Prokka-style GenBank files.

This script does not require Biopython. It finds annotated genes whose
names
start with pco or sil, extracts the smallest region spanning them,
adds optional
flanking sequence, and writes one cluster-only GenBank file per input
contig.
"""

from __future__ import annotations

import argparse
import datetime as dt
import re
from pathlib import Path
```

```
def parse_args() -> argparse.Namespace:
    parser = argparse.ArgumentParser(
        description="Extract pco/sil gene-cluster regions from GenBank
files."
    )
    parser.add_argument(
        "gbk",
        nargs="+",
        help="Input GenBank file(s). Shell globs are OK, e.g.
all_contigs/*.gbk",
    )
    parser.add_argument(
        "-o",
        "--outdir",
        default="pco_sil_clusters",
        help="Output directory. Default: pco_sil_clusters",
    )
    parser.add_argument(
        "--flank",
        type=int,
        default=2000,
        help="Bases to include upstream/downstream of the pco/sil
span. Default: 2000",
    )
    parser.add_argument(
        "--prefixes",
        default="pco,sil",
        help="Comma-separated gene prefixes to define the cluster.
Default: pco,sil",
    )
    return parser.parse_args()


def split_genbank_records(text: str) -> list[str]:
    records = []
    for block in text.split("\n//"):
        block = block.strip("\n")
        if block:
            records.append(block + "\n//\n")
    return records


def parse_sequence(lines: list[str]) -> str:
    in_origin = False
    seq_parts = []
    for line in lines:
        if line.startswith("ORIGIN"):
            in_origin = True
            continue
```

```
        if in_origin:
            if line.startswith("//"):
                break
            seq_parts.append(re.sub(r"[^A-Za-z]", "", line))
    return "".join(seq_parts).lower()


def parse_locus(lines: list[str], fallback: str) -> str:
    for line in lines:
        if line.startswith("LOCUS"):
            parts = line.split()
            if len(parts) > 1:
                return parts[1]
    return fallback


def parse_features(lines: list[str]) -> list[dict]:
    features = []
    in_features = False
    current = None

    def flush_current() -> None:
        if current is None:
            return
        start, end = location_span(current["location"])
        current["start"] = start
        current["end"] = end
        current["qualifier_text"] = "\n".join(current["qualifiers"])
        features.append(current.copy())

    for line in lines:
        if line.startswith("FEATURES"):
            in_features = True
            continue
        if line.startswith("ORIGIN"):
            flush_current()
            break
        if not in_features or not line.startswith("     "):
            continue

        key = line[5:21].strip()
        value = line[21:].rstrip()
        if key:
            flush_current()
            current = {"key": key, "location": value.strip(),
"qualifiers": []}
        elif current is not None:
            current["qualifiers"].append(value.strip())

    return features
```

```
def location_span(location: str) -> tuple[int | None, int | None]:
    nums = [int(x) for x in re.findall(r"\d+", location)]
    if not nums:
        return None, None
    return min(nums), max(nums)


def feature_gene(feature: dict) -> str:
    text = feature.get("qualifier_text", "")
    match = re.search(r'/gene="([^"]+)"', text)
    if match:
        return match.group(1)
    match = re.search(r'/locus_tag="([^"]+)"', text)
    if match:
        return match.group(1)
    return ""


def is_target_gene(feature: dict, prefixes: tuple[str, ...]) -> bool:
    gene = feature_gene(feature).lower()
    return any(gene.startswith(prefix) for prefix in prefixes)


def adjust_location(location: str, region_start: int) -> str:
    def repl(match: re.Match) -> str:
        return str(int(match.group(0)) - region_start + 1)

    return re.sub(r"\d+", repl, location)


def wrap_origin(seq: str) -> str:
    lines = ["ORIGIN"]
    for i in range(0, len(seq), 60):
        chunk = seq[i : i + 60]
        groups = " ".join(chunk[j : j + 10] for j in range(0,
len(chunk), 10))
        lines.append(f"{i + 1:>9} {groups}")
    return "\n".join(lines)


def safe_stem(path: Path) -> str:
    return re.sub(r"[^A-Za-z0-9_.-]+", "_", path.stem)


def write_cluster_gbk(
    outpath: Path,
    source_path: Path,
    record_locus: str,
```

```
    seq: str,
    features: list[dict],
    region_start: int,
    region_end: int,
) -> None:
    region_seq = seq[region_start - 1 : region_end]
    today = dt.datetime.now().strftime("%d-%b-%Y").upper()
    locus = safe_stem(outpath).replace("_pco_sil_cluster", "")

    with outpath.open("w") as handle:
        handle.write(f"LOCUS       {locus:<16}{len(region_seq):>11} bp
DNA     linear   {today}\n")
        handle.write(
            f"DEFINITION  pco/sil cluster from {source_path.name}; "
            f"original locus {record_locus}:{region_start}..
{region_end}.\n"
        )
        handle.write(f"ACCESSION   {locus}_pco_sil\n")
        handle.write(f"VERSION     {locus}_pco_sil\n")
        handle.write("KEYWORDS    pco; sil; copper resistance; silver
resistance.\n")
        handle.write("SOURCE      extracted sequence\n")
        handle.write("  ORGANISM  extracted sequence\n")
        handle.write("            Unclassified.\n")
        handle.write("FEATURES             Location/Qualifiers\n")
        handle.write(f"     source          1..{len(region_seq)}\n")
        handle.write(f'                     /note="Extracted from
{source_path.name}"\n')
        handle.write(
            f'                     /note="Original coordinates: '
            f'{record_locus}:{region_start}..{region_end}"\n'
        )

        for feature in features:
            start = feature["start"]
            end = feature["end"]
            if start is None or end is None:
                continue
            if start < region_start or end > region_end:
                continue
            adjusted = adjust_location(feature["location"],
region_start)
            handle.write(f"     {feature['key']:<16}{adjusted}\n")
            for qualifier in feature["qualifiers"]:
                if qualifier:
                    handle.write(f"                     {qualifier}
\n")

        handle.write(wrap_origin(region_seq))
        handle.write("\n//\n")
```

```
def process_file(path: Path, outdir: Path, flank: int, prefixes:
tuple[str, ...]) -> dict:
    text = path.read_text(errors="replace")
    records = split_genbank_records(text)
    if not records:
        return {"input": str(path), "status": "no_records"}

    results = []
    for idx, record_text in enumerate(records, start=1):
        lines = record_text.splitlines()
        seq = parse_sequence(lines)
        record_locus = parse_locus(lines, path.stem)
        features = parse_features(lines)
        targets = [
            f
            for f in features
            if f["start"] is not None and f["end"] is not None and
is_target_gene(f, prefixes)
        ]

        if not targets:
            results.append({"status": "no_pco_sil_genes"})
            continue

        raw_start = min(f["start"] for f in targets)
        raw_end = max(f["end"] for f in targets)
        region_start = max(1, raw_start - flank)
        region_end = min(len(seq), raw_end + flank)

        suffix = "" if len(records) == 1 else f"_record{idx}"
        outpath = outdir / f"{safe_stem(path)}{suffix}
_pco_sil_cluster.gbk"
        write_cluster_gbk(outpath, path, record_locus, seq, features,
region_start, region_end)

        target_genes = ",".join(feature_gene(f) for f in
sorted(targets, key=lambda x: x["start"]))
        results.append(
            {
                "status": "extracted",
                "output": str(outpath),
                "genes": target_genes,
                "target_start": raw_start,
                "target_end": raw_end,
                "extract_start": region_start,
                "extract_end": region_end,
                "extract_length": region_end - region_start + 1,
            }
```

```
        )

    return {"input": str(path), "records": results}


def main() -> None:
    args = parse_args()
    outdir = Path(args.outdir)
    outdir.mkdir(parents=True, exist_ok=True)
    prefixes = tuple(x.strip().lower() for x in
args.prefixes.split(",") if x.strip())

    summary_rows = [
        [
            "input",
            "status",
            "output",
            "genes",
            "target_start",
            "target_end",
            "extract_start",
            "extract_end",
            "extract_length",
        ]
    ]

    for raw_path in args.gbk:
        path = Path(raw_path)
        result = process_file(path, outdir, args.flank, prefixes)
        for record in result.get("records", [{"status":
result.get("status", "unknown")}]):
            summary_rows.append(
                [
                    result["input"],
                    record.get("status", ""),
                    record.get("output", ""),
                    record.get("genes", ""),
                    str(record.get("target_start", "")),
                    str(record.get("target_end", "")),
                    str(record.get("extract_start", "")),
                    str(record.get("extract_end", "")),
                    str(record.get("extract_length", "")),
                ]
            )

    summary_path = outdir / "pco_sil_cluster_extraction_summary.tsv"
    with summary_path.open("w") as handle:
        for row in summary_rows:
            handle.write("\t".join(row) + "\n")
```

```python
    print(f"Wrote summary: {summary_path}")


if __name__ == "__main__":
    main()
```


Script 6: extract_reference_gene_similarity.py
Source file: extract_reference_gene_similarity.py
Line count: 236


```python
#!/usr/bin/env python3
"""
Extract gene similarity values from a clinker alignments TSV.

The main use here is to compare each pco/sil isolate against the R478
reference and report the best target-gene match for every R478 gene.
"""

from __future__ import annotations

import argparse
import csv
import re
from collections import defaultdict
from pathlib import Path


def parse_args() -> argparse.Namespace:
    parser = argparse.ArgumentParser(
        description="Summarize clinker gene identity/similarity
against a reference."
    )
    parser.add_argument("alignments", help="clinker alignments TSV
file.")
    parser.add_argument(
        "-r",
        "--reference",
        default="R478",
        help="Reference cluster label to compare against. Default:
R478",
    )
    parser.add_argument(
        "-o",
        "--output",
        help="Long-format output TSV. Default: print to stdout",
```

```
    )
    parser.add_argument(
        "--matrix-output",
        help="Optional wide similarity-percent matrix TSV, genes x 
isolates.",
    )
    parser.add_argument(
        "--remove-label-text",
        action="append",
        default=["_pco_sil_only", "_pco_sil", "_only"],
        help=(
            "Text to remove from cluster labels in output. "
            "Defaults remove clinker pco/sil suffixes. Can be 
repeated."
        ),
    )
    parser.add_argument(
        "--best-by",
        choices=["similarity", "identity"],
        default="similarity",
        help="Metric used to choose the best hit when multiple genes 
align. Default: similarity",
    )
    parser.add_argument(
        "--same-gene-first",
        action="store_true",
        help="Prefer same-name target genes before choosing the best 
score.",
    )
    return parser.parse_args()


def clean_label(label: str, remove_texts: list[str]) -> str:
    for text in remove_texts:
        label = label.replace(text, "")
    return label


def label_matches_reference(label: str, reference: str, remove_texts: 
list[str]) -> bool:
    clean = clean_label(label, remove_texts)
    return clean == reference or label == reference or 
label.startswith(reference + "_")


def parse_alignment_sections(text: str) -> list[dict]:
    sections = []
    for raw_section in re.split(r"\n\s*\n", text.strip()):
        lines = [line.rstrip() for line in raw_section.splitlines() if 
line.strip()]
```

```
        if len(lines) < 4 or " vs " not in lines[0]:
            continue

        left, right = lines[0].split(" vs ", 1)
        rows = []
        for line in lines[3:]:
            parts = line.split()
            if len(parts) < 4:
                continue
            try:
                identity = float(parts[-2])
                similarity = float(parts[-1])
            except ValueError:
                continue
            rows.append(
                {
                    "query": parts[0],
                    "target": parts[1],
                    "identity": identity,
                    "similarity": similarity,
                }
            )
        sections.append({"left": left, "right": right, "rows": rows})
    return sections


def hit_sort_key(hit: dict, best_by: str, same_gene_first: bool) ->
tuple:
    same_gene = hit["reference_gene"] == hit["matched_gene"]
    same_gene_score = 1 if same_gene else 0
    primary = hit[best_by]
    secondary = hit["identity" if best_by == "similarity" else
"similarity"]
    if same_gene_first:
        return same_gene_score, primary, secondary
    return primary, secondary, same_gene_score


def summarize_reference_hits(
    sections: list[dict],
    reference: str,
    remove_texts: list[str],
    best_by: str,
    same_gene_first: bool,
) -> list[dict]:
    best_hits: dict[tuple[str, str], dict] = {}

    for section in sections:
        left = section["left"]
        right = section["right"]
```

```
        left_is_reference = label_matches_reference(left, reference,
remove_texts)
        right_is_reference = label_matches_reference(right, reference,
remove_texts)

        if left_is_reference == right_is_reference:
            continue

        isolate = clean_label(right if left_is_reference else left,
remove_texts)
        reference_cluster = clean_label(left if left_is_reference else
right, remove_texts)

        for row in section["rows"]:
            if left_is_reference:
                reference_gene = row["query"]
                matched_gene = row["target"]
            else:
                reference_gene = row["target"]
                matched_gene = row["query"]

            hit = {
                "reference": reference_cluster,
                "isolate": isolate,
                "reference_gene": reference_gene,
                "matched_gene": matched_gene,
                "identity": row["identity"],
                "similarity": row["similarity"],
                "identity_pct": row["identity"] * 100,
                "similarity_pct": row["similarity"] * 100,
            }
            key = (isolate, reference_gene)
            old = best_hits.get(key)
            if old is None or hit_sort_key(hit, best_by,
same_gene_first) > hit_sort_key(
                old, best_by, same_gene_first
            ):
                best_hits[key] = hit

    return sorted(best_hits.values(), key=lambda h: (h["isolate"],
natural_gene_key(h["reference_gene"])))


def natural_gene_key(value: str) -> tuple:
    return tuple(int(part) if part.isdigit() else part.lower() for
part in re.split(r"(\d+)", value))


def write_long_table(rows: list[dict], output: str | None) -> None:
    fieldnames = [
```

```
        "reference",
        "isolate",
        "reference_gene",
        "matched_gene",
        "identity",
        "similarity",
        "identity_pct",
        "similarity_pct",
    ]

    handle = open(output, "w", newline="") if output else None
    try:
        out = handle if handle is not None else None
        writer = csv.DictWriter(out or __import__("sys").stdout,
fieldnames=fieldnames, delimiter="\t")
        writer.writeheader()
        for row in rows:
            formatted = row.copy()
            formatted["identity"] = f"{row['identity']:.4f}"
            formatted["similarity"] = f"{row['similarity']:.4f}"
            formatted["identity_pct"] = f"{row['identity_pct']:.1f}"
            formatted["similarity_pct"] =
f"{row['similarity_pct']:.1f}"
            writer.writerow(formatted)
    finally:
        if handle is not None:
            handle.close()


def write_similarity_matrix(rows: list[dict], output: str) -> None:
    isolates = sorted({row["isolate"] for row in rows})
    genes = sorted({row["reference_gene"] for row in rows},
key=natural_gene_key)
    values = defaultdict(dict)
    for row in rows:
        values[row["reference_gene"]][row["isolate"]] =
row["similarity_pct"]

    with open(output, "w", newline="") as handle:
        writer = csv.writer(handle, delimiter="\t")
        writer.writerow(["reference_gene", *isolates])
        for gene in genes:
            writer.writerow(
                [gene, *[f"{values[gene][isolate]:.1f}" if isolate in
values[gene] else "" for isolate in isolates]]
            )


def main() -> None:
    args = parse_args()
```

```
    text = Path(args.alignments).read_text(errors="replace")
    sections = parse_alignment_sections(text)
    rows = summarize_reference_hits(
        sections,
        args.reference,
        args.remove_label_text,
        args.best_by,
        args.same_gene_first,
    )

    write_long_table(rows, args.output)
    if args.matrix_output:
        write_similarity_matrix(rows, args.matrix_output)

    if args.output:
        print(f"Wrote long table: {args.output}")
    if args.matrix_output:
        print(f"Wrote similarity matrix: {args.matrix_output}")
    print(f"Reference: {args.reference}")
    print(f"Comparisons summarized: {len({row['isolate'] for row in
rows})}")
    print(f"Reference genes summarized: {len({row['reference_gene']
for row in rows})}")


if __name__ == "__main__":
    main()
```


Script 7: annotate_clinker_similarity_on_figure.py
Source file: annotate_clinker_similarity_on_figure.py
Line count: 444


```python
#!/usr/bin/env python3
"""
Annotate a self-contained clinker HTML figure with similarity to a
reference.

This rewrites the embedded clinker data so genes are grouped/colored
by their
best similarity to a reference cluster such as R478. It also adds
hover metadata
fields to each gene: R478_similarity, R478_match, and
R478_similarity_pct.
The rendered right-side group legend is hidden so the figure remains
uncluttered.
```

```
"""

from __future__ import annotations

import argparse
import csv
import json
import re
from pathlib import Path
from typing import Any


DATA_PREFIX = "<script>const data="
DATA_SUFFIX = ";function serialise(svg)"

CATEGORIES = [
    ("reference", "R478 reference", "#b8b8b8"),
    ("very_high", "95-100% similar to R478", "#2f6df6"),
    ("high", "80-94% similar to R478", "#b3d8ff"),
    ("moderate", "50-79% similar to R478", "#fff2b2"),
    ("low", "<50% similar to R478", "#ffd6bf"),
    ("no_match", "No R478 match", "#eeeeee"),
]

GENE_LABEL_CONFIG = """      gene: {
        label: {
          show: true,
          fontSize: 9,
          position: "top",
          spacing: 3,
          rotation: 25,
        }
      },
      link: {
        label: {
          show: false,
        },
        groupColour: false,
      },
      legend: {
        show: false,
        fontSize: 11,
        entryHeight: 16,
      },
"""


def parse_args() -> argparse.Namespace:
    parser = argparse.ArgumentParser(
        description="Color/annotate clinker genes by similarity to a
```

```
reference cluster."
    )
    parser.add_argument("html", help="Input self-contained clinker
HTML.")
    parser.add_argument(
        "-o",
        "--output",
        help="Output HTML. Default: INPUT stem +
_R478_similarity.html",
    )
    parser.add_argument(
        "-r",
        "--reference",
        default="R478",
        help="Reference cluster label. Default: R478",
    )
    parser.add_argument(
        "--summary-tsv",
        help="Optional TSV with per-gene similarity category
assignments.",
    )
    parser.add_argument(
        "--remove-label-text",
        action="append",
        default=["_only"],
        help='Text to remove from labels before annotating. Default:
"_only". Can be repeated.',
    )
    parser.add_argument(
        "--very-high",
        type=float,
        default=0.95,
        help="Similarity cutoff for Very high. Default: 0.95",
    )
    parser.add_argument(
        "--high",
        type=float,
        default=0.80,
        help="Similarity cutoff for High. Default: 0.80",
    )
    parser.add_argument(
        "--moderate",
        type=float,
        default=0.50,
        help="Similarity cutoff for Moderate. Default: 0.50",
    )
    parser.add_argument(
        "--append-isolate-summary",
        action="store_true",
        help="Append an overall similarity category to each non-
```

```
reference left-side cluster label.",
    )
    parser.add_argument(
        "--hide-gene-labels",
        action="store_true",
        help="Do not turn on gene labels in the output HTML.",
    )
    return parser.parse_args()


def default_output_path(input_path: Path, reference: str) -> Path:
    return input_path.with_name(f"{input_path.stem}_{reference}
_similarity{input_path.suffix}")


def extract_data(text: str) -> tuple[str, Any, str]:
    start = text.find(DATA_PREFIX)
    if start == -1:
        raise ValueError(f"Could not find embedded data prefix:
{DATA_PREFIX!r}")

    json_start = start + len(DATA_PREFIX)
    end = text.find(DATA_SUFFIX, json_start)
    if end == -1:
        raise ValueError(f"Could not find embedded data suffix:
{DATA_SUFFIX!r}")

    return text[:json_start], json.loads(text[json_start:end]),
text[end:]


def clean_strings(value: Any, remove_texts: list[str]) -> Any:
    if isinstance(value, str):
        for text in remove_texts:
            value = value.replace(text, "")
        return value
    if isinstance(value, list):
        return [clean_strings(item, remove_texts) for item in value]
    if isinstance(value, dict):
        return {key: clean_strings(item, remove_texts) for key, item
in value.items()}
    return value


def clean_label(label: str, remove_texts: list[str]) -> str:
    for text in remove_texts:
        label = label.replace(text, "")
    return label
```

```
def normalize_gene(label: str) -> str:
    return re.sub(r"[_-]\d+$", "", label.strip()).lower()


def label_matches_reference(label: str, reference: str) -> bool:
    return label == reference or label.startswith(reference + "_")


def iter_cluster_genes(data: dict) -> list[tuple[dict, dict, dict]]:
    genes = []
    for cluster in data.get("clusters", []):
        for locus in cluster.get("loci", []):
            for gene in locus.get("genes", []):
                genes.append((cluster, locus, gene))
    return genes


def category_for_similarity(value: float | None, args:
argparse.Namespace) -> tuple[str, str]:
    if value is None:
        return "no_match", "No R478 match"
    if value >= args.very_high:
        return "very_high", "95-100% similar to R478"
    if value >= args.high:
        return "high", "80-94% similar to R478"
    if value >= args.moderate:
        return "moderate", "50-79% similar to R478"
    return "low", "<50% similar to R478"


def hit_sort_key(hit: dict) -> tuple:
    return (
        1 if hit["same_gene"] else 0,
        hit["similarity"],
        hit["identity"],
    )


def build_annotations(data: dict, reference: str, args:
argparse.Namespace) -> tuple[dict[str, dict], list[dict]]:
    gene_to_cluster: dict[str, str] = {}
    all_gene_uids: set[str] = set()
    reference_gene_uids: set[str] = set()

    for cluster, _locus, gene in iter_cluster_genes(data):
        cluster_name = cluster.get("name", "")
        uid = gene.get("uid")
        if not uid:
            continue
        gene_to_cluster[uid] = cluster_name
```

```
        all_gene_uids.add(uid)
        if label_matches_reference(cluster_name, reference):
            reference_gene_uids.add(uid)

    best_by_gene: dict[str, dict] = {}
    for link in data.get("links", []):
        query = link.get("query", {})
        target = link.get("target", {})
        query_uid = query.get("uid")
        target_uid = target.get("uid")
        if not query_uid or not target_uid:
            continue

        query_is_ref = query_uid in reference_gene_uids
        target_is_ref = target_uid in reference_gene_uids
        if query_is_ref == target_is_ref:
            continue

        ref_gene = query if query_is_ref else target
        other_gene = target if query_is_ref else query
        other_uid = other_gene.get("uid")
        if not other_uid:
            continue

        hit = {
            "reference_gene": ref_gene.get("label", ""),
            "matched_gene": other_gene.get("label", ""),
            "identity": float(link.get("identity", 0.0)),
            "similarity": float(link.get("similarity",
link.get("identity", 0.0))),
            "same_gene": normalize_gene(ref_gene.get("label", ""))
            == normalize_gene(other_gene.get("label", "")),
        }
        current = best_by_gene.get(other_uid)
        if current is None or hit_sort_key(hit) >
hit_sort_key(current):
            best_by_gene[other_uid] = hit

    annotations: dict[str, dict] = {}
    rows: list[dict] = []
    for cluster, _locus, gene in iter_cluster_genes(data):
        uid = gene.get("uid")
        if not uid:
            continue

        cluster_name = cluster.get("name", "")
        if uid in reference_gene_uids:
            category_id, category_label = "reference", "R478
reference"
            hit = {
```

```
                "reference_gene": gene.get("label", ""),
                "matched_gene": gene.get("label", ""),
                "identity": 1.0,
                "similarity": 1.0,
            }
        else:
            hit = best_by_gene.get(uid)
            category_id, category_label = category_for_similarity(
                hit["similarity"] if hit else None,
                args,
            )

        annotation = {
            "category_id": category_id,
            "category_label": category_label,
            "reference_gene": hit["reference_gene"] if hit else "",
            "identity": hit["identity"] if hit else None,
            "similarity": hit["similarity"] if hit else None,
        }
        annotations[uid] = annotation
        rows.append(
            {
                "cluster": cluster_name,
                "gene": gene.get("label", ""),
                "category": category_label,
                "R478_gene": annotation["reference_gene"],
                "identity_pct": "" if annotation["identity"] is None
else f"{annotation['identity'] * 100:.1f}",
                "similarity_pct": ""
                if annotation["similarity"] is None
                else f"{annotation['similarity'] * 100:.1f}",
            }
        )

    return annotations, rows


def apply_annotations_to_gene(gene: dict, annotation: dict) -> None:
    names = gene.setdefault("names", {})
    names["R478_similarity"] = annotation["category_label"]
    if annotation["reference_gene"]:
        names["R478_match"] = annotation["reference_gene"]
    if annotation["similarity"] is not None:
        names["R478_similarity_pct"] = f"{annotation['similarity'] *
100:.1f}"


def walk_and_annotate(value: Any, annotations: dict[str, dict]) ->
None:
    if isinstance(value, dict):
```

```
        uid = value.get("uid")
        if uid in annotations and isinstance(value.get("names"),
dict):
            apply_annotations_to_gene(value, annotations[uid])
        for item in value.values():
            walk_and_annotate(item, annotations)
    elif isinstance(value, list):
        for item in value:
            walk_and_annotate(item, annotations)


def replace_groups(data: dict, annotations: dict[str, dict]) -> None:
    grouped: dict[str, list[str]] = {category_id: [] for category_id,
_label, _colour in CATEGORIES}
    for uid, annotation in annotations.items():
        grouped.setdefault(annotation["category_id"], []).append(uid)

    groups = []
    for category_id, label, colour in CATEGORIES:
        genes = grouped.get(category_id, [])
        if not genes:
            continue
        groups.append(
            {
                "uid": f"R478_similarity_{category_id}",
                "label": label,
                "genes": genes,
                "hidden": False,
                "colour": colour,
            }
        )
    data["groups"] = groups


def preserve_custom_groups(data: dict) -> None:
    config = data.setdefault("config", {})
    config["updateGroups"] = False


def patch_plot_config(text: str, show_gene_labels: bool) -> str:
    if not show_gene_labels:
        return text

    old = """      gene: {
        label: {
          show: false,
        }
      },
"""
    if old not in text:
```

```
        return text
    return text.replace(old, GENE_LABEL_CONFIG, 1)


def inject_readable_label_css(text: str) -> str:
    css = """
text.geneLabel {
  fill: #111;
  paint-order: stroke;
  stroke: #fff;
  stroke-width: 3px;
  stroke-linejoin: round;
}
"""
    marker = "</style>"
    if css.strip() in text or marker not in text:
        return text
    return text.replace(marker, css + marker, 1)


def inject_hide_legend_css(text: str) -> str:
    css = """
svg.clusterMap g.legend {
  display: none !important;
  pointer-events: none;
}
"""
    marker = "</style>"
    if css.strip() in text or marker not in text:
        return text
    return text.replace(marker, css + marker, 1)


def append_isolate_summaries(data: dict, annotations: dict[str, dict],
reference: str) -> None:
    rank = {
        "reference": 4,
        "very_high": 3,
        "high": 2,
        "moderate": 1,
        "low": 0,
        "no_match": -1,
    }
    label_by_rank = {
        3: "mostly very high",
        2: "mostly high",
        1: "mostly moderate",
        0: "mostly low",
        -1: "mostly no match",
    }
```

```
    for cluster in data.get("clusters", []):
        name = cluster.get("name", "")
        if label_matches_reference(name, reference):
            continue
        scores = []
        for locus in cluster.get("loci", []):
            for gene in locus.get("genes", []):
                ann = annotations.get(gene.get("uid", ""))
                if ann and ann["category_id"] != "reference":
                    scores.append(rank[ann["category_id"]])
        if not scores:
            continue
        median_score = sorted(scores)[len(scores) // 2]
        cluster["name"] = f"{name} ({label_by_rank.get(median_score,
'mixed')})"


def write_summary(rows: list[dict], path: str) -> None:
    fieldnames = ["cluster", "gene", "category", "R478_gene",
"identity_pct", "similarity_pct"]
    with open(path, "w", newline="") as handle:
        writer = csv.DictWriter(handle, fieldnames=fieldnames,
delimiter="\t")
        writer.writeheader()
        writer.writerows(rows)


def main() -> None:
    args = parse_args()
    input_path = Path(args.html)
    output_path = Path(args.output) if args.output else
default_output_path(input_path, args.reference)

    text = input_path.read_text(errors="replace")
    before, data, after = extract_data(text)
    data = clean_strings(data, args.remove_label_text)

    annotations, rows = build_annotations(data, args.reference, args)
    walk_and_annotate(data, annotations)
    replace_groups(data, annotations)
    preserve_custom_groups(data)
    if args.append_isolate_summary:
        append_isolate_summaries(data, annotations, args.reference)

    output_text = before + json.dumps(data, separators=(",", ":")) +
after
    output_text = patch_plot_config(output_text, not
args.hide_gene_labels)
    output_text = inject_readable_label_css(output_text)
```

```
    output_text = inject_hide_legend_css(output_text)
    output_path.write_text(output_text)
    if args.summary_tsv:
        write_summary(rows, args.summary_tsv)

    print(f"Wrote annotated HTML: {output_path}")
    if args.summary_tsv:
        print(f"Wrote annotation summary: {args.summary_tsv}")
    print(f"Annotated genes: {len(annotations)}")
    print("Genes are lightly shaded by R478 similarity range.")


if __name__ == "__main__":
    main()
```


Script 8: make_clinker_gene_labels.py
Source file: make_clinker_gene_labels.py
Line count: 259


```python
#!/usr/bin/env python3
"""
Make GenBank copies with cleaner gene labels for clinker figures.

clinker often labels CDS arrows using /protein_id or /locus_tag.
Prokka/NCBI
files can therefore show protein accessions or locus numbers instead
of gene
names. This script creates visualization-only GenBank files where each
CDS has
a clean /gene and /locus_tag label, and /protein_id is removed so
clinker falls
back to that clean label.
"""

from __future__ import annotations

import argparse
import re
from pathlib import Path


QUALIFIER_INDENT = "                     "


def parse_args() -> argparse.Namespace:
```

```
    parser = argparse.ArgumentParser(
        description="Create clinker-friendly GenBank files with gene-
name labels."
    )
    parser.add_argument("gbk", nargs="+", help="Input GenBank
file(s).")
    parser.add_argument(
        "-o",
        "--outdir",
        required=True,
        help="Output directory for relabeled GenBank files.",
    )
    parser.add_argument(
        "--unknown-prefix",
        default="hypo",
        help="Prefix for CDS features without a real gene name.
Default: hypo",
    )
    parser.add_argument(
        "--unknown-separator",
        default=".",
        help="Separator for unnamed CDS numbering. Default: .",
    )
    return parser.parse_args()


def sanitize_label(value: str) -> str:
    value = value.strip()
    value = re.sub(r"\s+", "_", value)
    value = re.sub(r"[^A-Za-z0-9_.-]", "_", value)
    value = re.sub(r"_+", "_", value).strip("_")
    return value or "gene"


def get_qualifier(block: list[str], key: str) -> str | None:
    text = "\n".join(block)
    match = re.search(rf'/{re.escape(key)}="([^"]*)"', text,
flags=re.S)
    if not match:
        return None
    return re.sub(r"\s+", " ", match.group(1)).strip()


def canonical_product_gene(product: str) -> str | None:
    match = re.search(r"\b(pco[A-Za-z0-9_]*|sil[A-Za-z0-9_]*|cus[A-Za-
z0-9_]*|cop[A-Za-z0-9_]*)\b", product, flags=re.I)
    if not match:
        return None

    raw = match.group(1)
```

```
    prefix = raw[:3].lower()
    suffix = raw[3:]
    if len(suffix) == 1:
        suffix = suffix.upper()
    return sanitize_label(prefix + suffix)


def infer_label(block: list[str], index: int, unknown_prefix: str) ->
str:
    gene = get_qualifier(block, "gene")
    if gene:
        return sanitize_label(gene)

    product_raw = get_qualifier(block, "product") or ""
    product_gene = canonical_product_gene(product_raw)
    if product_gene:
        return product_gene

    product = product_raw.lower()

    product_rules = [
        ("silver-binding protein", "silE"),
        ("sensor kinase", "silS"),
        ("histidine kinase", "silS"),
        ("transcriptional regulatory", "silR"),
        ("outer membrane", "silC"),
        ("outer-membrane", "silC"),
        ("periplasmic adaptor", "silB"),
        ("permease", "silA"),
        ("p-type atpase", "silP"),
        ("atpase", "silP"),
        ("copper resistance protein a", "pcoA"),
        ("copper resistance protein b", "pcoB"),
        ("copper resistance protein c", "pcoC"),
        ("copper resistance protein d", "pcoD"),
        ("copper-binding protein", "pcoE"),
        ("murein dd-endopeptidase", "mepM"),
    ]

    for needle, label in product_rules:
        if needle in product:
            return label

    return sanitize_label(unknown_prefix)


def uniquify(
    label: str,
    seen: dict[str, int],
    unknown_prefix: str,
```

```
    unknown_separator: str,
) -> str:
    if label not in seen:
        seen[label] = 1
        if label == unknown_prefix:
            return f"{label}{unknown_separator}1"
        return label

    seen[label] += 1
    if label == unknown_prefix:
        return f"{label}{unknown_separator}{seen[label]}"
    return f"{label}_{seen[label]}"


def split_feature_blocks(lines: list[str]) -> list[list[str] | str]:
    chunks: list[list[str] | str] = []
    current: list[str] | None = None
    in_features = False

    for line in lines:
        if line.startswith("FEATURES"):
            in_features = True
            chunks.append(line)
            continue
        if line.startswith("ORIGIN"):
            if current is not None:
                chunks.append(current)
                current = None
            in_features = False
            chunks.append(line)
            continue
        if not in_features:
            chunks.append(line)
            continue

        is_feature_start = line.startswith("     ") and
line[5:21].strip()
        if is_feature_start:
            if current is not None:
                chunks.append(current)
            current = [line]
        elif current is not None:
            current.append(line)
        else:
            chunks.append(line)

    if current is not None:
        chunks.append(current)
    return chunks
```

```
def feature_key(block: list[str]) -> str:
    if not block:
        return ""
    return block[0][5:21].strip()


def replace_or_insert_qualifier(block: list[str], key: str, value:
str) -> list[str]:
    pattern = re.compile(rf"{re.escape(QUALIFIER_INDENT)}/
{re.escape(key)}=")
    new_block = []
    replaced = False

    for line in block:
        if pattern.match(line):
            if not replaced:
                new_block.append(f'{QUALIFIER_INDENT}/{key}
="{value}"')
                replaced = True
            continue
        new_block.append(line)

    if not replaced:
        insert_at = 1
        new_block.insert(insert_at, f'{QUALIFIER_INDENT}/{key}
="{value}"')
    return new_block


def remove_qualifier(block: list[str], key: str) -> list[str]:
    pattern = re.compile(rf"{re.escape(QUALIFIER_INDENT)}/
{re.escape(key)}=")
    return [line for line in block if not pattern.match(line)]


def relabel_file(
    input_path: Path,
    output_path: Path,
    unknown_prefix: str,
    unknown_separator: str,
) -> None:
    lines = input_path.read_text(errors="replace").splitlines()
    chunks = split_feature_blocks(lines)

    locus_to_label: dict[str, str] = {}
    seen: dict[str, int] = {}
    cds_index = 0

    for chunk in chunks:
```

```
        if not isinstance(chunk, list) or feature_key(chunk) != "CDS":
            continue
        cds_index += 1
        old_locus = get_qualifier(chunk, "locus_tag")
        label = uniquify(
            infer_label(chunk, cds_index, unknown_prefix),
            seen,
            unknown_prefix,
            unknown_separator,
        )
        if old_locus:
            locus_to_label[old_locus] = label

    relabeled: list[str] = []
    for chunk in chunks:
        if not isinstance(chunk, list):
            relabeled.append(chunk)
            continue

        key = feature_key(chunk)
        old_locus = get_qualifier(chunk, "locus_tag")
        label = locus_to_label.get(old_locus or "")

        if label:
            chunk = replace_or_insert_qualifier(chunk, "locus_tag",
label)
            if key in {"gene", "CDS"}:
                chunk = replace_or_insert_qualifier(chunk, "gene",
label)
            if key == "CDS":
                chunk = remove_qualifier(chunk, "protein_id")

        relabeled.extend(chunk)

    output_path.write_text("\n".join(relabeled) + "\n")


def main() -> None:
    args = parse_args()
    outdir = Path(args.outdir)
    outdir.mkdir(parents=True, exist_ok=True)

    for raw in args.gbk:
        input_path = Path(raw)
        output_path = outdir / input_path.name
        relabel_file(input_path, output_path, args.unknown_prefix,
args.unknown_separator)
        print(f"Wrote {output_path}")
```

```
if __name__ == "__main__":
    main()
```


Script 9: orient_pco_sil_clusters.py
Source file: orient_pco_sil_clusters.py
Line count: 527


```python
#!/usr/bin/env python3
"""
Orient pco/sil GenBank cluster files consistently for clinker figures.

The script can auto-detect clusters whose pco/sil marker genes run
opposite to
the expected order, reverse-complement those records, and write a
complete set
of oriented GenBank files to a new output directory. It does not
require
Biopython.
"""

from __future__ import annotations

import argparse
import datetime as dt
import re
import shutil
from pathlib import Path


FEATURE_KEY_START = 5
FEATURE_KEY_END = 21
QUALIFIER_INDENT = " " * FEATURE_KEY_END
DEFAULT_ANCHOR_ORDER = (
    "silS,silC,silB,silA,silP,silE,pcoA,pcoB,pcoC,pcoD,pcoR,pcoS,pcoE"
)
DNA_COMPLEMENT = str.maketrans(
    "ACGTRYKMSWBDHVNacgtrykmswbdhvn",
    "TGCAYRMKSWVHDBNtgcayrmkswvhdbn",
)


def parse_args() -> argparse.Namespace:
    parser = argparse.ArgumentParser(
        description="Reverse-complement reversed pco/sil cluster
GenBank files."
```

```
    )
    parser.add_argument("gbk", nargs="+", help="Input GenBank
file(s).")
    parser.add_argument(
        "-o",
        "--outdir",
        required=True,
        help="Output directory for oriented GenBank files.",
    )
    parser.add_argument(
        "--anchor-order",
        default=DEFAULT_ANCHOR_ORDER,
        help=(
            "Comma-separated gene order used for auto-detection. "
            f"Default: {DEFAULT_ANCHOR_ORDER}"
        ),
    )
    parser.add_argument(
        "--flip",
        action="append",
        default=[],
        metavar="NAME",
        help=(
            "Force a file to be reverse-complemented. NAME may be a
filename, "
            "stem, or isolate label. Can be used multiple times."
        ),
    )
    parser.add_argument(
        "--flip-list",
        help="Optional text file with one filename/stem/isolate label
to flip per line.",
    )
    parser.add_argument(
        "--no-auto",
        action="store_true",
        help="Disable automatic orientation detection and use only --
flip/--flip-list.",
    )
    parser.add_argument(
        "--min-matches",
        type=int,
        default=4,
        help="Minimum anchor genes required before auto-calling
orientation. Default: 4",
    )
    parser.add_argument(
        "--min-margin",
        type=int,
        default=2,
```

```
        help="Minimum directional-vote margin for an auto flip/keep
call. Default: 2",
    )
    parser.add_argument(
        "--dry-run",
        action="store_true",
        help="Report planned actions without writing output files.",
    )
    parser.add_argument(
        "--in-place",
        action="store_true",
        help="Overwrite input files in place. A backup is written
unless --backup-ext '' is used.",
    )
    parser.add_argument(
        "--backup-ext",
        default=".orientation.bak",
        help="Backup extension for --in-place.
Default: .orientation.bak",
    )
    return parser.parse_args()


def split_genbank_records(text: str) -> list[str]:
    records = []
    for block in text.split("\n//"):
        block = block.strip("\n")
        if block:
            records.append(block + "\n//\n")
    return records


def parse_sequence(lines: list[str]) -> str:
    in_origin = False
    parts = []
    for line in lines:
        if line.startswith("ORIGIN"):
            in_origin = True
            continue
        if in_origin:
            if line.startswith("//"):
                break
            parts.append(re.sub(r"[^A-Za-z]", "", line))
    return "".join(parts)


def wrap_origin(seq: str) -> list[str]:
    lines = ["ORIGIN"]
    seq = seq.lower()
    for i in range(0, len(seq), 60):
```

```
        chunk = seq[i : i + 60]
        groups = " ".join(chunk[j : j + 10] for j in range(0,
len(chunk), 10))
        lines.append(f"{i + 1:>9} {groups}")
    return lines


def reverse_complement(seq: str) -> str:
    return seq.translate(DNA_COMPLEMENT)[::-1]


def normalize_gene(name: str) -> str:
    name = name.strip()
    name = re.sub(r"[_-]\d+$", "", name)
    if len(name) >= 4 and name[:3].lower() in {"pco", "sil"}:
        return name[:3].lower() + name[3:].upper()
    return name


def feature_key(line: str) -> str:
    if not line.startswith("     "):
        return ""
    return line[FEATURE_KEY_START:FEATURE_KEY_END].strip()


def split_feature_blocks(lines: list[str]) -> tuple[list[str],
list[list[str]], list[str]]:
    before_features: list[str] = []
    blocks: list[list[str]] = []
    after_features: list[str] = []
    current: list[str] | None = None
    in_features = False
    in_after = False

    for line in lines:
        if line.startswith("FEATURES"):
            in_features = True
            before_features.append(line)
            continue
        if line.startswith("ORIGIN"):
            if current is not None:
                blocks.append(current)
                current = None
            in_features = False
            in_after = True
            after_features.append(line)
            continue
        if in_after:
            after_features.append(line)
            continue
```

```
        if not in_features:
            before_features.append(line)
            continue

        if feature_key(line):
            if current is not None:
                blocks.append(current)
            current = [line]
        elif current is not None:
            current.append(line)
        else:
            before_features.append(line)

    if current is not None:
        blocks.append(current)
    return before_features, blocks, after_features


def split_top_level_commas(text: str) -> list[str]:
    parts = []
    depth = 0
    start = 0
    for i, char in enumerate(text):
        if char == "(":
            depth += 1
        elif char == ")":
            depth -= 1
        elif char == "," and depth == 0:
            parts.append(text[start:i].strip())
            start = i + 1
    parts.append(text[start:].strip())
    return parts


def top_level_operator(text: str) -> tuple[str, str] | None:
    stripped = text.strip()
    match = re.match(r"^([A-Za-z_][A-Za-z0-9_]*)\((.*)\)$", stripped)
    if not match:
        return None

    depth = 0
    inner_start = stripped.find("(")
    for i, char in enumerate(stripped[inner_start:],
start=inner_start):
        if char == "(":
            depth += 1
        elif char == ")":
            depth -= 1
            if depth == 0 and i != len(stripped) - 1:
                return None
```

```
    return match.group(1), match.group(2)


def transform_interval(match: re.Match[str], seq_len: int) -> str:
    left_mark = match.group("left_mark") or ""
    start = int(match.group("start"))
    sep = match.group("sep")
    right_mark = match.group("right_mark") or ""
    end = int(match.group("end"))

    new_start = seq_len - end + 1
    new_end = seq_len - start + 1
    new_left_mark = "<" if right_mark == ">" else ""
    new_right_mark = ">" if left_mark == "<" else ""
    return f"{new_left_mark}{new_start}{sep}{new_right_mark}{new_end}"


def transform_location_body(text: str, seq_len: int) -> str:
    text = text.strip()
    operator = top_level_operator(text)
    if operator and operator[0] in {"join", "order", "bond"}:
        op, inner = operator
        parts = [transform_location_body(part, seq_len) for part in
split_top_level_commas(inner)]
        parts.sort(key=location_min)
        return f"{op}({','.join(parts)})"
    if operator and operator[0] == "complement":
        inner = transform_location_body(operator[1], seq_len)
        return f"complement({inner})"

    interval_pattern = re.compile(
        r"(?P<left_mark>[<>]?)(?P<start>\d+)"
        r"(?P<sep>\.\.|\^)"
        r"(?P<right_mark>[<>]?)(?P<end>\d+)"
    )
    intervals: list[str] = []

    def interval_repl(match: re.Match[str]) -> str:
        token = f"__LOC_INTERVAL_{len(intervals)}__"
        intervals.append(transform_interval(match, seq_len))
        return token

    text = interval_pattern.sub(interval_repl, text)

    def single_repl(match: re.Match[str]) -> str:
        return str(seq_len - int(match.group(0)) + 1)

    text = re.sub(r"(?<![A-Za-z0-9_])\d+(?![A-Za-z0-9_])",
single_repl, text)
    for i, interval in enumerate(intervals):
```

```
        text = text.replace(f"__LOC_INTERVAL_{i}__", interval)
    return text


def location_min(location: str) -> int:
    nums = [int(x) for x in re.findall(r"\d+", location)]
    return min(nums) if nums else 0


def reverse_location(location: str, seq_len: int) -> str:
    location = location.strip()
    operator = top_level_operator(location)
    if operator and operator[0] == "complement":
        return transform_location_body(operator[1], seq_len)
    transformed = transform_location_body(location, seq_len)
    return f"complement({transformed})"


def collect_location_lines(block: list[str]) -> tuple[str, int]:
    location_parts = [block[0][FEATURE_KEY_END:].strip()]
    consumed = 1
    while consumed < len(block):
        stripped = block[consumed].strip()
        if stripped.startswith("/"):
            break
        location_parts.append(stripped)
        consumed += 1
    return "".join(location_parts), consumed


def wrap_feature_location(key: str, location: str) -> list[str]:
    first_prefix = f"     {key:<16}"
    continuation_prefix = QUALIFIER_INDENT
    width = 79
    available_first = width - len(first_prefix)
    available_next = width - len(continuation_prefix)

    if len(location) <= available_first:
        return [first_prefix + location]

    lines = [first_prefix + location[:available_first]]
    location = location[available_first:]
    while location:
        lines.append(continuation_prefix + location[:available_next])
        location = location[available_next:]
    return lines


def reverse_feature_block(block: list[str], seq_len: int) ->
list[str]:
```

```
    key = feature_key(block[0])
    location, consumed = collect_location_lines(block)
    new_location = reverse_location(location, seq_len)
    new_block = wrap_feature_location(key, new_location)
    new_block.extend(block[consumed:])
    return new_block


def reverse_record(record_text: str) -> str:
    lines = record_text.splitlines()
    seq = parse_sequence(lines)
    if not seq:
        raise ValueError("record has no ORIGIN sequence")

    before_features, blocks, _after_features =
split_feature_blocks(lines)
    seq_len = len(seq)
    reversed_blocks = [reverse_feature_block(block, seq_len) for block
in blocks]

    out_lines: list[str] = []
    out_lines.extend(before_features)
    for block in sorted(reversed_blocks, key=lambda b:
location_min(b[0][FEATURE_KEY_END:])):
        out_lines.extend(block)
    out_lines.extend(wrap_origin(reverse_complement(seq)))
    out_lines.append("//")
    return "\n".join(out_lines) + "\n"


def extract_gene_order(record_text: str) -> list[str]:
    lines = record_text.splitlines()
    _, blocks, _ = split_feature_blocks(lines)
    rows: list[tuple[int, str]] = []

    for block in blocks:
        key = feature_key(block[0])
        if key != "CDS":
            continue
        location, _ = collect_location_lines(block)
        text = "\n".join(block)
        match = re.search(r'/gene="([^"]+)"', text)
        if not match:
            continue
        gene = normalize_gene(match.group(1))
        if gene.lower().startswith(("pco", "sil")):
            rows.append((location_min(location), gene))

    if not rows:
        for block in blocks:
```

```
            key = feature_key(block[0])
            if key != "gene":
                continue
            location, _ = collect_location_lines(block)
            text = "\n".join(block)
            match = re.search(r'/gene="([^"]+)"', text)
            if not match:
                continue
            gene = normalize_gene(match.group(1))
            if gene.lower().startswith(("pco", "sil")):
                rows.append((location_min(location), gene))

    ordered = []
    seen_adjacent: str | None = None
    for _, gene in sorted(rows):
        if gene != seen_adjacent:
            ordered.append(gene)
        seen_adjacent = gene
    return ordered


def orientation_call(
    genes: list[str],
    anchor_order: list[str],
    min_matches: int,
    min_margin: int,
) -> tuple[str, str]:
    anchor_index = {normalize_gene(gene): i for i, gene in
enumerate(anchor_order)}
    indices = [anchor_index[gene] for gene in genes if gene in
anchor_index]

    if len(indices) < min_matches:
        return "keep", f"only {len(indices)} anchor genes matched"

    forward = 0
    reverse = 0
    for left, right in zip(indices, indices[1:]):
        if right > left:
            forward += 1
        elif right < left:
            reverse += 1

    if reverse - forward >= min_margin:
        return "flip", f"reverse trend {reverse} vs forward trend
{forward}"
    if forward - reverse >= min_margin:
        return "keep", f"forward trend {forward} vs reverse trend
{reverse}"
    return "keep", f"ambiguous trend forward={forward},
```

```
reverse={reverse}"


def load_forced_flips(args: argparse.Namespace) -> set[str]:
    names = set(args.flip)
    if args.flip_list:
        for line in
Path(args.flip_list).read_text(errors="replace").splitlines():
            line = line.strip()
            if line and not line.startswith("#"):
                names.add(line)
    return {normalize_match_name(name) for name in names}


def normalize_match_name(name: str) -> str:
    name = Path(name).name
    name = re.sub(r"\.(gbk|gb|gbff|genbank)$", "", name, flags=re.I)
    name = re.sub(r"_pco_sil_cluster$", "", name, flags=re.I)
    name = re.sub(r"[_-]contig[_-]?\d+", "", name, flags=re.I)
    return name.lower()


def matches_forced_flip(path: Path, forced: set[str]) -> bool:
    candidates = {
        normalize_match_name(path.name),
        normalize_match_name(path.stem),
        path.name.lower(),
        path.stem.lower(),
    }
    return bool(candidates & forced)


def orient_file(
    path: Path,
    output_path: Path,
    action: str,
    dry_run: bool,
    in_place: bool,
    backup_ext: str,
) -> None:
    if dry_run:
        return

    text = path.read_text(errors="replace")
    if action == "flip":
        records = split_genbank_records(text)
        new_text = "".join(reverse_record(record) for record in
records)
    else:
        new_text = text
```

```
    if in_place:
        if backup_ext:
            backup_path = path.with_name(path.name + backup_ext)
            if not backup_path.exists():
                shutil.copy2(path, backup_path)
        path.write_text(new_text)
        return

    output_path.parent.mkdir(parents=True, exist_ok=True)
    output_path.write_text(new_text)


def write_summary(outdir: Path, rows: list[list[str]], dry_run: bool)
-> None:
    if dry_run:
        return
    summary_path = outdir / "pco_sil_orientation_summary.tsv"
    with summary_path.open("w") as handle:
        handle.write("input\taction\treason\tgenes\toutput\n")
        for row in rows:
            handle.write("\t".join(row) + "\n")


def main() -> None:
    args = parse_args()
    paths = [Path(raw) for raw in args.gbk]
    outdir = Path(args.outdir)
    anchor_order = [normalize_gene(gene.strip()) for gene in
args.anchor_order.split(",") if gene.strip()]
    forced_flips = load_forced_flips(args)
    rows: list[list[str]] = []

    for path in paths:
        text = path.read_text(errors="replace")
        records = split_genbank_records(text)
        first_record = records[0] if records else text
        genes = extract_gene_order(first_record)

        if matches_forced_flip(path, forced_flips):
            action = "flip"
            reason = "forced by --flip/--flip-list"
        elif args.no_auto:
            action = "keep"
            reason = "auto-detection disabled"
        else:
            action, reason = orientation_call(
                genes,
                anchor_order,
                args.min_matches,
```

```
                args.min_margin,
            )

        output_path = path if args.in_place else outdir / path.name
        orient_file(path, output_path, action, args.dry_run,
args.in_place, args.backup_ext)
        rows.append([str(path), action, reason, ",".join(genes),
str(output_path)])
        print(f"{path.name}: {action} ({reason})")

    if not args.in_place:
        write_summary(outdir, rows, args.dry_run)
        if not args.dry_run:
            print(f"Wrote oriented files: {outdir}")
            print(f"Wrote summary: {outdir /
'pco_sil_orientation_summary.tsv'}")
    elif not args.dry_run:
        print(f"Updated files in place on
{dt.datetime.now().isoformat(timespec='seconds')}")


if __name__ == "__main__":
    main()
```


Script 10: clean_clinker_html_labels.py
Source file: clean_clinker_html_labels.py
Line count: 121


```python
#!/usr/bin/env python3
"""
Clean labels in a self-contained clinker/clustermap HTML file.

By default this removes "_only" from embedded labels and removes the
group
legend/labels from the right side of the rendered figure by emptying
the
embedded "groups" list.
"""

from __future__ import annotations

import argparse
import json
from pathlib import Path
from typing import Any
```

```
DATA_PREFIX = "<script>const data="
DATA_SUFFIX = ";function serialise(svg)"


def parse_args() -> argparse.Namespace:
    parser = argparse.ArgumentParser(
        description="Remove suffix text and group labels from clinker
HTML output."
    )
    parser.add_argument("html", help="Input self-contained clinker
HTML file.")
    parser.add_argument(
        "-o",
        "--output",
        help="Output HTML path. Default: INPUT stem +
_labels_cleaned.html",
    )
    parser.add_argument(
        "--remove-text",
        action="append",
        default=["_only"],
        help='Text to remove from embedded labels. Default: "_only".
Can be repeated.',
    )
    parser.add_argument(
        "--keep-groups",
        action="store_true",
        help="Keep the groups data instead of removing the right-side
group labels/legend.",
    )
    parser.add_argument(
        "--blank-group-labels",
        action="store_true",
        help="Keep groups for coloring but set each group label to an
empty string.",
    )
    return parser.parse_args()


def default_output_path(input_path: Path) -> Path:
    return input_path.with_name(f"{input_path.stem}
_labels_cleaned{input_path.suffix}")


def extract_data(text: str) -> tuple[str, Any, str]:
    start = text.find(DATA_PREFIX)
    if start == -1:
        raise ValueError(f"Could not find embedded data prefix:
```

```
{DATA_PREFIX!r}")

    json_start = start + len(DATA_PREFIX)
    end = text.find(DATA_SUFFIX, json_start)
    if end == -1:
        raise ValueError(f"Could not find embedded data suffix:
{DATA_SUFFIX!r}")

    before = text[:json_start]
    raw_json = text[json_start:end]
    after = text[end:]
    return before, json.loads(raw_json), after


def clean_strings(value: Any, remove_texts: list[str]) -> Any:
    if isinstance(value, str):
        for text in remove_texts:
            value = value.replace(text, "")
        return value
    if isinstance(value, list):
        return [clean_strings(item, remove_texts) for item in value]
    if isinstance(value, dict):
        return {key: clean_strings(item, remove_texts) for key, item
in value.items()}
    return value


def remove_groups(data: Any, blank_labels: bool, keep_groups: bool) ->
None:
    if not isinstance(data, dict) or "groups" not in data:
        return

    if keep_groups:
        return
    if blank_labels and isinstance(data["groups"], list):
        for group in data["groups"]:
            if isinstance(group, dict):
                group["label"] = ""
        return
    data["groups"] = []


def main() -> None:
    args = parse_args()
    input_path = Path(args.html)
    output_path = Path(args.output) if args.output else
default_output_path(input_path)

    text = input_path.read_text(errors="replace")
    before, data, after = extract_data(text)
```

```
    data = clean_strings(data, args.remove_text)
    remove_groups(data, args.blank_group_labels, args.keep_groups)

    cleaned_json = json.dumps(data, separators=(",", ":"))
    output_path.write_text(before + cleaned_json + after)

    print(f"Wrote cleaned HTML: {output_path}")
    print(f"Removed text: {', '.join(args.remove_text)}")
    if args.keep_groups:
        print("Groups: kept")
    elif args.blank_group_labels:
        print("Groups: labels blanked")
    else:
        print("Groups: removed from embedded data")


if __name__ == "__main__":
    main()
```


Script 11: search_cusabfcrs_genes.py
Source file: search_cusabfcrs_genes.py
Line count: 486


```python
#!/usr/bin/env python3
"""
Search GenBank files for CusABFCRS genes.

By default this scans the oriented pco/sil cluster GenBank files in
pco_sil_clusters_oriented/ and reports CDS features matching:

    cusA, cusB, cusF, cusC, cusR, cusS

The script also maps common silver-system annotations onto Cus
homologs:
silA->cusA, silB/sibB->cusB, silF->cusF, silC->cusC, silR->cusR,
and silS->cusS. R478's older CusX annotation is treated as cusF.
"""

from __future__ import annotations

import argparse
import csv
import re
import sys
from dataclasses import dataclass
```

```python
from pathlib import Path


FEATURE_KEY_START = 5
FEATURE_KEY_END = 21
TARGET_ORDER = ("cusA", "cusB", "cusF", "cusC", "cusR", "cusS")
TARGET_RANK = {gene: i for i, gene in enumerate(TARGET_ORDER)}
CANONICAL_TARGET = {gene.lower(): gene for gene in TARGET_ORDER}
ALIAS_TO_TARGET = {
    "sila": "cusA",
    "silb": "cusB",
    "sibb": "cusB",
    "silf": "cusF",
    "silc": "cusC",
    "silr": "cusR",
    "sils": "cusS",
}
SOURCE_PRIORITY = {
    "gene": 0,
    "locus_tag": 1,
    "gene_alias": 2,
    "locus_tag_alias": 3,
    "product": 4,
    "note_cusx_synonym": 5,
    "note": 6,
}
CUS_TOKEN_RE = re.compile(
    r"(?<![A-Za-z0-9])cus[-_\s]*([abfcrsx])(?=[^A-Za-z0-9]|$)",
    re.IGNORECASE,
)


@dataclass(frozen=True)
class Feature:
    source_file: Path
    isolate: str
    record_id: str
    feature_type: str
    location: str
    start: int | None
    end: int | None
    strand: str
    qualifiers: dict[str, list[str]]


def parse_args() -> argparse.Namespace:
    parser = argparse.ArgumentParser(
        description="Search R478, pSH111_227, and NCTR GenBank files
for CusABFCRS genes."
    )
```

```
    parser.add_argument(
        "gbk",
        nargs="*",
        help=(
            "Input GenBank file(s). Default: all *.gbk files in "
            "pco_sil_clusters_oriented/."
        ),
    )
    parser.add_argument(
        "--gbk-dir",
        default="pco_sil_clusters_oriented",
        help="Directory to scan when no input files are given.
Default: pco_sil_clusters_oriented",
    )
    parser.add_argument(
        "-o",
        "--output",
        help="Long-format hit TSV. Default: print to stdout.",
    )
    parser.add_argument(
        "--matrix-output",
        help="Optional presence matrix TSV with one row per isolate.",
    )
    parser.add_argument(
        "--protein-fasta",
        help="Optional FASTA file containing matched CDS
translations.",
    )
    parser.add_argument(
        "--include-note-homologs",
        action="store_true",
        help=(
            "Search /note text for all Cus terms. By default, /note
text is "
            "used only for the old CusX->cusF synonym to avoid broad
homolog hits."
        ),
    )
    parser.add_argument(
        "--no-aliases",
        action="store_true",
        help="Disable sil/sib alias mapping and use only literal Cus
annotations.",
    )
    return parser.parse_args()


def discover_inputs(args: argparse.Namespace) -> list[Path]:
    if args.gbk:
        paths = [Path(path) for path in args.gbk]
```

```
    else:
        paths = sorted(Path(args.gbk_dir).glob("*.gbk"))

    missing = [str(path) for path in paths if not path.exists()]
    if missing:
        raise SystemExit(f"Input file(s) not found: {',
'.join(missing)}")
    if not paths:
        raise SystemExit("No GenBank files found to scan.")
    return paths


def split_genbank_records(text: str) -> list[str]:
    records = []
    for block in text.split("\n//"):
        block = block.strip("\n")
        if block:
            records.append(block + "\n//\n")
    return records


def record_id_from_lines(lines: list[str]) -> str:
    for prefix in ("VERSION", "ACCESSION", "LOCUS"):
        for line in lines:
            if line.startswith(prefix):
                parts = line.split()
                if len(parts) >= 2:
                    return parts[1]
    return "unknown"


def feature_key(line: str) -> str:
    if not line.startswith("     "):
        return ""
    return line[FEATURE_KEY_START:FEATURE_KEY_END].strip()


def split_feature_blocks(lines: list[str]) -> list[list[str]]:
    blocks: list[list[str]] = []
    current: list[str] | None = None
    in_features = False

    for line in lines:
        if line.startswith("FEATURES"):
            in_features = True
            continue
        if line.startswith("ORIGIN"):
            break
        if not in_features:
            continue
```

```
        if feature_key(line):
            if current is not None:
                blocks.append(current)
            current = [line]
        elif current is not None:
            current.append(line)

    if current is not None:
        blocks.append(current)
    return blocks


def clean_qualifier_value(value: str) -> str:
    value = value.strip()
    if value.startswith('"'):
        value = value[1:]
    if value.endswith('"'):
        value = value[:-1]
    return re.sub(r"\s+", " ", value).strip()


def parse_feature_block(
    block: list[str],
    source_file: Path,
    isolate: str,
    record_id: str,
) -> Feature:
    feature_type = block[0][FEATURE_KEY_START:FEATURE_KEY_END].strip()
    location_parts = [block[0][FEATURE_KEY_END:].strip()]
    qualifiers: dict[str, list[str]] = {}
    current_key: str | None = None
    current_parts: list[str] = []

    def flush_qualifier() -> None:
        nonlocal current_key, current_parts
        if current_key is None:
            return
        qualifiers.setdefault(current_key,
[]).append(clean_qualifier_value(" ".join(current_parts)))
        current_key = None
        current_parts = []

    for line in block[1:]:
        text = line[FEATURE_KEY_END:].rstrip()
        stripped = text.strip()
        if stripped.startswith("/"):
            flush_qualifier()
            key_value = stripped[1:]
            if "=" in key_value:
```

```
                current_key, value = key_value.split("=", 1)
                current_parts = [value]
            else:
                current_key = key_value
                current_parts = [""]
        elif current_key is not None:
            current_parts.append(stripped)
        elif stripped:
            location_parts.append(stripped)

    flush_qualifier()
    location = "".join(location_parts)
    start, end = parse_location_bounds(location)
    strand = "-" if "complement" in location else "+"
    return Feature(
        source_file=source_file,
        isolate=isolate,
        record_id=record_id,
        feature_type=feature_type,
        location=location,
        start=start,
        end=end,
        strand=strand,
        qualifiers=qualifiers,
    )


def parse_location_bounds(location: str) -> tuple[int | None, int |
None]:
    numbers = [int(value) for value in re.findall(r"\d+", location)]
    if not numbers:
        return None, None
    return min(numbers), max(numbers)


def parse_genbank(path: Path) -> list[Feature]:
    text = path.read_text(errors="replace")
    isolate = clean_isolate_label(path.stem)
    features: list[Feature] = []
    for record in split_genbank_records(text):
        lines = record.splitlines()
        record_id = record_id_from_lines(lines)
        for block in split_feature_blocks(lines):
            features.append(parse_feature_block(block, path, isolate,
record_id))
    return features


def clean_isolate_label(label: str) -> str:
    for suffix in ("_pco_sil_only", "_pco_sil", "_only"):
```

```
        label = label.replace(suffix, "")
    return label


def normalize_gene_name(value: str) -> str:
    value = value.strip().lower()
    value = re.sub(r"[_-]\d+$", "", value)
    return re.sub(r"[^a-z0-9]", "", value)


def target_from_gene_name(value: str, use_aliases: bool) -> tuple[str,
str] | None:
    normalized = normalize_gene_name(value)
    if normalized in CANONICAL_TARGET:
        return CANONICAL_TARGET[normalized], "literal"
    if use_aliases and normalized in ALIAS_TO_TARGET:
        return ALIAS_TO_TARGET[normalized], "alias"
    return None


def targets_from_text(text: str) -> list[tuple[str, str]]:
    targets = []
    for match in CUS_TOKEN_RE.finditer(text):
        letter = match.group(1).lower()
        target = "cusF" if letter == "x" else
CANONICAL_TARGET[f"cus{letter}"]
        targets.append((target, match.group(0)))
    return targets


def pick_qualifier(feature: Feature, key: str) -> str:
    return "; ".join(feature.qualifiers.get(key, []))


def feature_matches(
    feature: Feature,
    use_aliases: bool,
    include_note_homologs: bool,
) -> list[dict]:
    if feature.feature_type != "CDS":
        return []

    matches: dict[str, dict] = {}

    def add_match(target: str, source: str, value: str) -> None:
        row = {
            "target_cus_gene": target,
            "match_source": source,
            "match_value": value,
        }
```

```
        old = matches.get(target)
        if old is None or SOURCE_PRIORITY[source] <
SOURCE_PRIORITY[old["match_source"]]:
            matches[target] = row

    for key in ("gene", "locus_tag"):
        for value in feature.qualifiers.get(key, []):
            target = target_from_gene_name(value, use_aliases)
            if target is None:
                continue
            target_gene, match_type = target
            source = key if match_type == "literal" else f"{key}
_alias"
            add_match(target_gene, source, value)

    for value in feature.qualifiers.get("product", []):
        for target, matched_text in targets_from_text(value):
            add_match(target, "product", matched_text)

    for value in feature.qualifiers.get("note", []):
        for target, matched_text in targets_from_text(value):
            if include_note_homologs:
                add_match(target, "note", matched_text)
            elif target == "cusF" and
matched_text.lower().replace("_", "").replace(" ", "") == "cusx":
                add_match(target, "note_cusx_synonym", matched_text)

    return [matches[target] for target in sorted(matches, key=lambda
g: TARGET_RANK[g])]


def hit_rows(
    features: list[Feature],
    use_aliases: bool,
    include_note_homologs: bool,
) -> list[dict]:
    rows = []
    for feature in features:
        for match in feature_matches(feature, use_aliases,
include_note_homologs):
            start = feature.start if feature.start is not None else ""
            end = feature.end if feature.end is not None else ""
            length_nt = (
                feature.end - feature.start + 1
                if feature.start is not None and feature.end is not
None
                else ""
            )
            rows.append(
                {
```

```
                    "isolate": feature.isolate,
                    "source_file": str(feature.source_file),
                    "record_id": feature.record_id,
                    "target_cus_gene": match["target_cus_gene"],
                    "gene": pick_qualifier(feature, "gene"),
                    "locus_tag": pick_qualifier(feature, "locus_tag"),
                    "feature_type": feature.feature_type,
                    "start": start,
                    "end": end,
                    "strand": feature.strand,
                    "length_nt": length_nt,
                    "product": pick_qualifier(feature, "product"),
                    "match_source": match["match_source"],
                    "match_value": match["match_value"],
                    "translation": pick_qualifier(feature,
"translation"),
                }
            )
    return sorted(rows, key=hit_sort_key)


def isolate_sort_key(label: str) -> tuple:
    if label == "R478":
        return (0, label)
    if label == "pSH111_227":
        return (1, label)
    match = re.match(r"NCTR(\d+)$", label)
    if match:
        return (2, int(match.group(1)))
    return (3, label)


def hit_sort_key(row: dict) -> tuple:
    start = row["start"] if isinstance(row["start"], int) else 0
    return (isolate_sort_key(row["isolate"]),
TARGET_RANK[row["target_cus_gene"]], start)


def write_hits(rows: list[dict], output: str | None) -> None:
    fieldnames = [
        "isolate",
        "source_file",
        "record_id",
        "target_cus_gene",
        "gene",
        "locus_tag",
        "feature_type",
        "start",
        "end",
        "strand",
```

```
        "length_nt",
        "product",
        "match_source",
        "match_value",
    ]
    handle = open(output, "w", newline="") if output else sys.stdout
    try:
        writer = csv.DictWriter(handle, fieldnames=fieldnames,
delimiter="\t")
        writer.writeheader()
        for row in rows:
            writer.writerow({field: row[field] for field in
fieldnames})
    finally:
        if output:
            handle.close()


def matrix_value(rows: list[dict]) -> str:
    values = []
    for row in sorted(rows, key=lambda r: (int(r["start"] or 0),
r["gene"], r["locus_tag"])):
        name = row["gene"] or row["locus_tag"] or
row["target_cus_gene"]
        coord = f"{row['start']}-{row['end']}({row['strand']})"
        values.append(f"{name}@{coord}")
    return ";".join(values)


def write_matrix(rows: list[dict], output: str) -> None:
    grouped: dict[tuple[str, str], list[dict]] = {}
    for row in rows:
        grouped.setdefault((row["isolate"], row["target_cus_gene"]),
[]).append(row)

    isolates = sorted({row["isolate"] for row in rows},
key=isolate_sort_key)
    with open(output, "w", newline="") as handle:
        writer = csv.writer(handle, delimiter="\t")
        writer.writerow(["isolate", *TARGET_ORDER])
        for isolate in isolates:
            writer.writerow(
                [
                    isolate,
                    *[
                        matrix_value(grouped.get((isolate, target),
[]))
                        for target in TARGET_ORDER
                    ],
                ]
```

```
            )


def fasta_wrap(sequence: str, width: int = 60) -> str:
    return "\n".join(sequence[i : i + width] for i in range(0,
len(sequence), width))


def write_protein_fasta(rows: list[dict], output: str) -> None:
    with open(output, "w") as handle:
        for row in rows:
            sequence = re.sub(r"[^A-Za-z*]", "", row["translation"])
            if not sequence:
                continue
            name = row["gene"] or row["locus_tag"] or
row["target_cus_gene"]
            header = (
                f"{row['isolate']}|{row['target_cus_gene']}|{name}|"
                f"{row['start']}-{row['end']}({row['strand']})"
            )
            handle.write(f">{header}\n{fasta_wrap(sequence)}\n")


def main() -> None:
    args = parse_args()
    paths = discover_inputs(args)
    features = []
    for path in paths:
        features.extend(parse_genbank(path))

    rows = hit_rows(
        features,
        use_aliases=not args.no_aliases,
        include_note_homologs=args.include_note_homologs,
    )
    write_hits(rows, args.output)
    if args.matrix_output:
        write_matrix(rows, args.matrix_output)
    if args.protein_fasta:
        write_protein_fasta(rows, args.protein_fasta)


if __name__ == "__main__":
    main()
```


Script 12: search_cus_genes_pco_sil_wide.py
Source file: search_cus_genes_pco_sil_wide.py

Line count: 148

```python
#!/usr/bin/env python3
"""
Search Cus genes in the pco/sil wide GenBank files.

Default input:
    ~/Desktop/clinker_Project1/pco_sil_clusters_wide_display_names/*.gbk

Default outputs are written beside this script:
    cus_genes_pco_sil_wide_hits.tsv
    cus_genes_pco_sil_wide_matrix.tsv
    cus_genes_pco_sil_wide_proteins.faa

This searches the CusABFCRS set and maps common pco/sil-cluster labels onto
their Cus homologs: silA->cusA, silB/sibB->cusB, silF->cusF, silC->cusC,
silR->cusR, and silS->cusS. R478's older CusX annotation is treated as cusF.
"""

from __future__ import annotations

import argparse
import sys
from pathlib import Path

from search_cusabfcrs_genes import (
    hit_rows,
    parse_genbank,
    write_hits,
    write_matrix,
    write_protein_fasta,
)


SCRIPT_DIR = Path(__file__).resolve().parent
DEFAULT_GBK_DIR = (
    Path.home()
    / "Desktop"
    / "clinker_Project1"
    / "pco_sil_clusters_wide_display_names"
)
DEFAULT_HITS = SCRIPT_DIR / "cus_genes_pco_sil_wide_hits.tsv"
DEFAULT_MATRIX = SCRIPT_DIR / "cus_genes_pco_sil_wide_matrix.tsv"
DEFAULT_FASTA = SCRIPT_DIR / "cus_genes_pco_sil_wide_proteins.faa"
```

```
def parse_args() -> argparse.Namespace:
    parser = argparse.ArgumentParser(
        description=(
            "Search all isolate and reference pco/sil wide GenBank
files for "
            "CusABFCRS genes/homologs."
        )
    )
    parser.add_argument(
        "gbk",
        nargs="*",
        help="Optional GenBank file(s). Default: all *.gbk files in
the wide pco/sil folder.",
    )
    parser.add_argument(
        "--gbk-dir",
        default=str(DEFAULT_GBK_DIR),
        help=f"Wide pco/sil GenBank directory. Default:
{DEFAULT_GBK_DIR}",
    )
    parser.add_argument(
        "-o",
        "--output",
        default=str(DEFAULT_HITS),
        help=f"Long-format hit TSV. Default: {DEFAULT_HITS}",
    )
    parser.add_argument(
        "--matrix-output",
        default=str(DEFAULT_MATRIX),
        help=f"Presence/coordinate matrix TSV. Default:
{DEFAULT_MATRIX}",
    )
    parser.add_argument(
        "--protein-fasta",
        default=str(DEFAULT_FASTA),
        help=f"Matched protein FASTA. Default: {DEFAULT_FASTA}",
    )
    parser.add_argument(
        "--no-protein-fasta",
        action="store_true",
        help="Do not write matched protein FASTA output.",
    )
    parser.add_argument(
        "--literal-only",
        action="store_true",
        help="Only report literal cus* annotations; disable sil/sib
alias mapping.",
    )
```

```
    parser.add_argument(
        "--include-note-homologs",
        action="store_true",
        help=(
            "Also scan /note text for Cus homolog mentions. This can 
add broad "
            "homolog hits such as pco/cop genes that mention Cus in 
similarity notes."
        ),
    )
    return parser.parse_args()


def discover_inputs(args: argparse.Namespace) -> list[Path]:
    if args.gbk:
        paths = [Path(path).expanduser() for path in args.gbk]
    else:
        gbk_dir = Path(args.gbk_dir).expanduser()
        paths = sorted(gbk_dir.glob("*.gbk"))

    missing = [str(path) for path in paths if not path.exists()]
    if missing:
        raise SystemExit(f"Input file(s) not found: {', 
'.join(missing)}")
    if not paths:
        raise SystemExit(f"No .gbk files found in {args.gbk_dir}")
    return paths


def main() -> None:
    args = parse_args()
    paths = discover_inputs(args)

    features = []
    for path in paths:
        features.extend(parse_genbank(path))

    rows = hit_rows(
        features,
        use_aliases=not args.literal_only,
        include_note_homologs=args.include_note_homologs,
    )

    write_hits(rows, args.output)
    if args.matrix_output:
        write_matrix(rows, args.matrix_output)
    if args.protein_fasta and not args.no_protein_fasta:
        write_protein_fasta(rows, args.protein_fasta)

    isolates = sorted({row["isolate"] for row in rows})
```

```
    print(
        f"Scanned {len(paths)} GenBank files; found {len(rows)} Cus hits "
        f"across {len(isolates)} isolates/reference strains.",
        file=sys.stderr,
    )
    print(f"Wrote: {args.output}", file=sys.stderr)
    if args.matrix_output:
        print(f"Wrote: {args.matrix_output}", file=sys.stderr)
    if args.protein_fasta and not args.no_protein_fasta:
        print(f"Wrote: {args.protein_fasta}", file=sys.stderr)


if __name__ == "__main__":
    main()
```

##
##################
D. MOB-suite plasmid prediction table generation
##
##################

Script 13: combine_mob_results.py
Source file: combine_mob_results.py
Line count: 113


```python
#!/usr/bin/env python3
"""Combine MOB-suite mobtyper_results.txt files by contig group.

The script expects folders like:

  mob_results_contig2/NCTR117_contig2/mobtyper_results.txt
  mob_results_contig3/NCTR117_contig3/mobtyper_results.txt

It writes one combined TSV for contig2 and one for contig3.
"""

from __future__ import annotations

import argparse
import csv
from pathlib import Path


HELPER_COLUMNS = ["contig_set", "source_folder", "isolate", "contig"]
```

```
def parse_sample_folder(folder_name: str) -> tuple[str, str]:
    """Return isolate and contig name from a folder like
NCTR117_contig2."""
    if "_contig" not in folder_name:
        return folder_name, ""

    isolate, contig_number = folder_name.rsplit("_contig", 1)
    return isolate, f"contig{contig_number}"


def read_mobtyper_tables(results_dir: Path, contig_set: str) ->
tuple[list[str], list[dict[str, str]]]:
    """Read all mobtyper_results.txt files from one MOB-suite results
directory."""
    rows: list[dict[str, str]] = []
    fieldnames: list[str] = []

    for result_file in sorted(results_dir.glob("*/
mobtyper_results.txt")):
        source_folder = result_file.parent.name
        isolate, contig = parse_sample_folder(source_folder)

        with result_file.open(newline="") as handle:
            reader = csv.DictReader(handle, delimiter="\t")
            if reader.fieldnames is None:
                continue

            for field in reader.fieldnames:
                if field not in fieldnames:
                    fieldnames.append(field)

            for row in reader:
                rows.append(
                    {
                        "contig_set": contig_set,
                        "source_folder": source_folder,
                        "isolate": isolate,
                        "contig": contig,
                        **row,
                    }
                )

    return HELPER_COLUMNS + fieldnames, rows


def write_tsv(path: Path, fieldnames: list[str], rows: list[dict[str,
str]]) -> None:
    path.parent.mkdir(parents=True, exist_ok=True)
```

```
    with path.open("w", newline="") as handle:
        writer = csv.DictWriter(handle, delimiter="\t",
fieldnames=fieldnames, extrasaction="ignore")
        writer.writeheader()
        for row in rows:
            writer.writerow(row)


def main() -> int:
    parser = argparse.ArgumentParser(
        description="Combine MOB-suite mobtyper_results.txt files into
contig2 and contig3 summary tables."
    )
    parser.add_argument(
        "--project-dir",
        type=Path,
        default=Path("."),
        help="Project folder containing mob_results_contig2 and
mob_results_contig3.",
    )
    parser.add_argument(
        "--output-dir",
        type=Path,
        default=None,
        help="Folder for combined output tables. Defaults to
PROJECT_DIR/mob_combined_tables.",
    )
    args = parser.parse_args()

    project_dir = args.project_dir.expanduser().resolve()
    output_dir = args.output_dir.expanduser().resolve() if
args.output_dir else project_dir / "mob_combined_tables"

    jobs = [
        ("contig2", project_dir / "mob_results_contig2", output_dir /
"contig2_mobtyper_combined.tsv"),
        ("contig3", project_dir / "mob_results_contig3", output_dir /
"contig3_mobtyper_combined.tsv"),
    ]

    for contig_set, results_dir, output_file in jobs:
        if not results_dir.exists():
            raise FileNotFoundError(f"Missing results directory:
{results_dir}")

        fieldnames, rows = read_mobtyper_tables(results_dir,
contig_set)
        if not rows:
            raise ValueError(f"No mobtyper_results.txt rows found in
{results_dir}")
```

```
        write_tsv(output_file, fieldnames, rows)
        print(f"Wrote {len(rows)} rows: {output_file}")

    return 0


if __name__ == "__main__":
    raise SystemExit(main())
```


Script 14: make_contig2_plasmid_type_table.py
Source file: make_contig2_plasmid_type_table.py
Line count: 195


```python
#!/usr/bin/env python3
"""Create a colored plasmid-type table for contig2 MOB-suite
results."""

from __future__ import annotations

import argparse
import csv
import html
from pathlib import Path


DEFAULT_PROJECT_DIR = Path("<project_directory>")
DEFAULT_OUTPUT_DIR = Path("<output_directory>/amr_gene_comparison")

COLOR_BY_TYPE = {
    "IncHI2A": "#6c63b8",
    "IncHI2": "#6c63b8",
    "IncC": "#b8324c",
    "IncX1": "#2d7fb8",
    "IncI-gamma/K1": "#159a7d",
    "IncI1/B/O": "#d4861d",
    "unknown": "#777777",
}


def clean_plasmid_type(rep_types: str) -> str:
    """Return a display plasmid type from MOB-suite rep_type(s)."""
    if not rep_types or rep_types == "-":
        return "unknown"
```

```
    parts = [part.strip() for part in rep_types.split(",") if
part.strip()]
    inc_parts = [part for part in parts if part.startswith("Inc")]
    if inc_parts:
        return ",".join(inc_parts)

    return ",".join(parts) if parts else "unknown"


def format_size(value: str) -> str:
    try:
        return f"{int(float(value)):,} bp"
    except (TypeError, ValueError):
        return value


def read_rows(input_tsv: Path) -> list[dict[str, str]]:
    rows: list[dict[str, str]] = []
    with input_tsv.open(newline="") as handle:
        reader = csv.DictReader(handle, delimiter="\t")
        for row in reader:
            plasmid_type = clean_plasmid_type(row.get("rep_type(s)",
""))
            cluster = "/".join(
                value
                for value in [
                    row.get("primary_cluster_id", "").strip(),
                    row.get("secondary_cluster_id", "").strip(),
                ]
                if value and value != "-"
            )
            rows.append(
                {
                    "isolate": row.get("isolate", "").strip(),
                    "contig2_plasmid_type": plasmid_type,
                    "size": format_size(row.get("size", "").strip()),
                    "mobility": row.get("predicted_mobility",
"").strip() or "-",
                    "cluster": cluster or "-",
                    "nearest_neighbor":
row.get("mash_nearest_neighbor", "").strip() or "-",
                    "rep_type_raw": row.get("rep_type(s)", "").strip()
or "-",
                }
            )

    return sorted(rows, key=lambda row: row["isolate"])


def write_tsv(path: Path, rows: list[dict[str, str]]) -> None:
```

```
    columns = [
        "isolate",
        "contig2_plasmid_type",
        "size",
        "mobility",
        "cluster",
        "nearest_neighbor",
        "rep_type_raw",
    ]
    with path.open("w", newline="") as handle:
        writer = csv.DictWriter(handle, delimiter="\t",
fieldnames=columns)
        writer.writeheader()
        writer.writerows(rows)


def color_for_type(plasmid_type: str) -> str:
    return COLOR_BY_TYPE.get(plasmid_type, "#4f7f8f")


def write_svg(path: Path, rows: list[dict[str, str]]) -> None:
    width = 900
    row_h = 34
    top = 104
    height = top + len(rows) * row_h + 58

    isolate_x = 95
    type_x = 230
    size_x = 405
    mob_x = 540
    cluster_x = 690

    svg: list[str] = []
    svg.append(
        f'<svg xmlns="http://www.w3.org/2000/svg" width="{width}"
height="{height}" '
        f'viewBox="0 0 {width} {height}">'
    )
    svg.append('<rect width="100%" height="100%" fill="#ffffff"/>')
    svg.append(
        "<style>"
        "text{font-family:Arial,Helvetica,sans-serif}"
        ".title{font-size:18px;font-weight:700}"
        ".head{font-size:12px;font-weight:700}"
        ".txt{font-size:12px}"
        "</style>"
    )
    svg.append('<text x="24" y="32" class="title">Contig 2 Plasmid
Types Across NCTR Isolates</text>')
    svg.append(
```

```
        '<text x="24" y="56" class="txt" fill="#444">'
        "Plasmid types based on MOB-suite replicon/mobility
classification."
        "</text>"
    )

    header_y = 88
    svg.append(f'<text x="{isolate_x}" y="{header_y}"
class="head">Isolate</text>')
    svg.append(f'<text x="{type_x}" y="{header_y}"
class="head">Plasmid type</text>')
    svg.append(f'<text x="{size_x}" y="{header_y}" class="head">Size</
text>')
    svg.append(f'<text x="{mob_x}" y="{header_y}"
class="head">Mobility</text>')
    svg.append(f'<text x="{cluster_x}" y="{header_y}"
class="head">Cluster</text>')
    svg.append(f'<line x1="24" y1="98" x2="{width - 24}" y2="98"
stroke="#d8d8d8" stroke-width="1"/>')

    for i, row in enumerate(rows):
        y = top + i * row_h + 21
        band_y = top + i * row_h
        plasmid_type = row["contig2_plasmid_type"]
        fill = color_for_type(plasmid_type)

        if i % 2 == 0:
            svg.append(f'<rect x="24" y="{band_y}" width="{width -
48}" height="{row_h}" fill="#f7f7f7"/>')

        svg.append(f'<text x="{isolate_x}" y="{y}"
class="txt">{html.escape(row["isolate"])}</text>')
        svg.append(f'<rect x="{type_x}" y="{y - 17}" width="130"
height="22" rx="3" fill="{fill}"/>')
        svg.append(
            f'<text x="{type_x + 65}" y="{y - 2}" class="txt" text-
anchor="middle" fill="#ffffff">'
            f'{html.escape(plasmid_type)}</text>'
        )
        svg.append(f'<text x="{size_x}" y="{y}"
class="txt">{html.escape(row["size"])}</text>')
        svg.append(f'<text x="{mob_x}" y="{y}"
class="txt">{html.escape(row["mobility"])}</text>')
        svg.append(f'<text x="{cluster_x}" y="{y}"
class="txt">{html.escape(row["cluster"])}</text>')

    svg.append("</svg>")
    path.write_text("\n".join(svg))
```

```
def main() -> int:
    parser = argparse.ArgumentParser(description="Make a colored
contig2 plasmid-type table from MOB-suite output.")
    parser.add_argument("--project-dir", type=Path,
default=DEFAULT_PROJECT_DIR)
    parser.add_argument("--input", type=Path, default=None,
help="Combined contig2 MOB-typer TSV.")
    parser.add_argument("--output-dir", type=Path,
default=DEFAULT_OUTPUT_DIR)
    args = parser.parse_args()

    project_dir = args.project_dir.expanduser().resolve()
    input_tsv = (
        args.input.expanduser().resolve()
        if args.input
        else project_dir / "mob_combined_tables" /
"contig2_mobtyper_combined.tsv"
    )
    output_dir = args.output_dir.expanduser().resolve()
    output_dir.mkdir(parents=True, exist_ok=True)

    rows = read_rows(input_tsv)
    if not rows:
        raise ValueError(f"No rows found in {input_tsv}")

    tsv_path = output_dir / "contig2_plasmid_types.tsv"
    svg_path = output_dir / "contig2_plasmid_types.svg"
    fixed_svg_path = output_dir / "contig2_plasmid_types_fixed.svg"

    write_tsv(tsv_path, rows)
    write_svg(svg_path, rows)
    write_svg(fixed_svg_path, rows)

    print(f"Wrote {len(rows)} rows")
    print(tsv_path)
    print(svg_path)
    print(fixed_svg_path)
    return 0


if __name__ == "__main__":
    raise SystemExit(main())
```


Script 15: make_contig2_plasmid_type_table_standalone.py
Source file: make_contig2_plasmid_type_table_standalone.py
Line count: 264

```python
#!/usr/bin/env python3
"""
Make a colored plasmid-type table for contig2 MOB-suite results.

This is a standalone script. It does not need Codex.

Expected input:
  mob_combined_tables/contig2_mobtyper_combined.tsv

Outputs:
  Clinker_Output/amr_gene_comparison/contig2_plasmid_types.tsv
  Clinker_Output/amr_gene_comparison/contig2_plasmid_types.svg
  Clinker_Output/amr_gene_comparison/contig2_plasmid_types_fixed.svg

Run from your project folder:
  cd <project_directory>
  python3 codes/make_contig2_plasmid_type_table_standalone.py

Or provide paths manually:
  python3 make_contig2_plasmid_type_table_standalone.py \
    --input mob_combined_tables/contig2_mobtyper_combined.tsv \
    --output-dir Clinker_Output/amr_gene_comparison
"""

from __future__ import annotations

import argparse
import csv
import html
from pathlib import Path


COLOR_BY_TYPE = {
    "IncHI2A": "#6c63b8",
    "IncHI2": "#6c63b8",
    "IncHI2/FIB-like": "#8b6bb8",
    "IncC": "#b8324c",
    "IncX1": "#2d7fb8",
    "IncI-gamma/K1": "#159a7d",
    "IncI1/B/O": "#d4861d",
    "unknown": "#777777",
}


REFERENCE_ROWS = [
    {
        "isolate": "R478",
        "contig2_plasmid_type": "IncHI2",
```

```
        "size": "274,762 bp",
        "mobility": "conjugative",
        "cluster": "reference",
        "nearest_neighbor": "R478",
        "rep_type_raw": "IncHI2 reference plasmid",
    },
    {
        "isolate": "pSH111_227",
        "contig2_plasmid_type": "IncHI2/FIB-like",
        "size": "227,608 bp",
        "mobility": "conjugative",
        "cluster": "reference",
        "nearest_neighbor": "pSH111_227",
        "rep_type_raw": "repHI2; RepFIB-like reference plasmid",
    },
]


def clean_plasmid_type(rep_types: str) -> str:
    """Extract a clean plasmid type from the MOB-suite rep_type(s)
field."""
    if not rep_types or rep_types == "-":
        return "unknown"

    parts = [part.strip() for part in rep_types.split(",") if
part.strip()]
    inc_parts = [part for part in parts if part.startswith("Inc")]

    if inc_parts:
        return ",".join(inc_parts)

    return ",".join(parts) if parts else "unknown"


def format_size(value: str) -> str:
    """Format plasmid size as 261,448 bp."""
    try:
        return f"{int(float(value)):,} bp"
    except (TypeError, ValueError):
        return value or "-"


def read_rows(input_tsv: Path) -> list[dict[str, str]]:
    rows: list[dict[str, str]] = []

    with input_tsv.open(newline="") as handle:
        reader = csv.DictReader(handle, delimiter="\t")

        for row in reader:
            plasmid_type = clean_plasmid_type(row.get("rep_type(s)",
```

```
""))

            primary_cluster = row.get("primary_cluster_id",
"").strip()
            secondary_cluster = row.get("secondary_cluster_id",
"").strip()
            cluster = "/".join(
                value for value in [primary_cluster,
secondary_cluster] if value and value != "-"
            )

            rows.append(
                {
                    "isolate": row.get("isolate", "").strip(),
                    "contig2_plasmid_type": plasmid_type,
                    "size": format_size(row.get("size", "").strip()),
                    "mobility": row.get("predicted_mobility",
"").strip() or "-",
                    "cluster": cluster or "-",
                    "nearest_neighbor":
row.get("mash_nearest_neighbor", "").strip() or "-",
                    "rep_type_raw": row.get("rep_type(s)", "").strip()
or "-",
                }
            )

    return sorted(rows, key=lambda row: row["isolate"])


def with_reference_rows(rows: list[dict[str, str]]) -> list[dict[str,
str]]:
    """Add reference plasmids at the top of the table."""
    existing = {row["isolate"] for row in rows}
    references = [row for row in REFERENCE_ROWS if row["isolate"] not
in existing]
    return references + rows


def write_tsv(path: Path, rows: list[dict[str, str]]) -> None:
    columns = [
        "isolate",
        "contig2_plasmid_type",
        "size",
        "mobility",
        "cluster",
        "nearest_neighbor",
        "rep_type_raw",
    ]

    with path.open("w", newline="") as handle:
```

```
        writer = csv.DictWriter(handle, delimiter="\t",
fieldnames=columns)
        writer.writeheader()
        writer.writerows(rows)


def color_for_type(plasmid_type: str) -> str:
    return COLOR_BY_TYPE.get(plasmid_type, "#4f7f8f")


def write_svg(path: Path, rows: list[dict[str, str]]) -> None:
    width = 900
    row_h = 34
    top = 104
    height = top + len(rows) * row_h + 58

    isolate_x = 95
    type_x = 230
    size_x = 405
    mob_x = 540
    cluster_x = 690

    svg: list[str] = []
    svg.append(
        f'<svg xmlns="http://www.w3.org/2000/svg" width="{width}"
height="{height}" '
        f'viewBox="0 0 {width} {height}">'
    )
    svg.append('<rect width="100%" height="100%" fill="#ffffff"/>')
    svg.append(
        "<style>"
        "text{font-family:Arial,Helvetica,sans-serif}"
        ".title{font-size:18px;font-weight:700}"
        ".head{font-size:12px;font-weight:700}"
        ".txt{font-size:12px}"
        "</style>"
    )
    svg.append('<text x="24" y="32" class="title">Contig 2 Plasmid
Types Across NCTR Isolates</text>')
    svg.append(
        '<text x="24" y="56" class="txt" fill="#444">'
        "Plasmid types based on MOB-suite replicon/mobility
classification."
        "</text>"
    )

    header_y = 88
    svg.append(f'<text x="{isolate_x}" y="{header_y}"
class="head">Isolate</text>')
    svg.append(f'<text x="{type_x}" y="{header_y}"
```

```
class="head">Plasmid type</text>')
    svg.append(f'<text x="{size_x}" y="{header_y}" class="head">Size</
text>')
    svg.append(f'<text x="{mob_x}" y="{header_y}"
class="head">Mobility</text>')
    svg.append(f'<text x="{cluster_x}" y="{header_y}"
class="head">Cluster</text>')
    svg.append(f'<line x1="24" y1="98" x2="{width - 24}" y2="98"
stroke="#d8d8d8" stroke-width="1"/>')

    for i, row in enumerate(rows):
        y = top + i * row_h + 21
        band_y = top + i * row_h
        plasmid_type = row["contig2_plasmid_type"]
        fill = color_for_type(plasmid_type)

        if i % 2 == 0:
            svg.append(f'<rect x="24" y="{band_y}" width="{width -
48}" height="{row_h}" fill="#f7f7f7"/>')

        svg.append(f'<text x="{isolate_x}" y="{y}"
class="txt">{html.escape(row["isolate"])}</text>')
        svg.append(f'<rect x="{type_x}" y="{y - 17}" width="130"
height="22" rx="3" fill="{fill}"/>')
        svg.append(
            f'<text x="{type_x + 65}" y="{y - 2}" class="txt" text-
anchor="middle" fill="#ffffff">'
            f'{html.escape(plasmid_type)}</text>'
        )
        svg.append(f'<text x="{size_x}" y="{y}"
class="txt">{html.escape(row["size"])}</text>')
        svg.append(f'<text x="{mob_x}" y="{y}"
class="txt">{html.escape(row["mobility"])}</text>')
        svg.append(f'<text x="{cluster_x}" y="{y}"
class="txt">{html.escape(row["cluster"])}</text>')

    svg.append("</svg>")
    path.write_text("\n".join(svg))


def main() -> int:
    parser = argparse.ArgumentParser(
        description="Make a colored contig2 plasmid-type table from
MOB-suite combined results."
    )
    parser.add_argument(
        "--input",
        type=Path,
        default=Path("mob_combined_tables/
contig2_mobtyper_combined.tsv"),
```

```
        help="Input combined MOB-typer TSV.",
    )
    parser.add_argument(
        "--output-dir",
        type=Path,
        default=Path("Clinker_Output/amr_gene_comparison"),
        help="Folder for output TSV and SVG files.",
    )
    parser.add_argument(
        "--no-references",
        action="store_true",
        help="Do not add R478 and pSH111_227 reference plasmids to the
table.",
    )
    args = parser.parse_args()

    input_tsv = args.input.expanduser().resolve()
    output_dir = args.output_dir.expanduser().resolve()
    output_dir.mkdir(parents=True, exist_ok=True)

    if not input_tsv.exists():
        raise FileNotFoundError(f"Input file not found: {input_tsv}")

    rows = read_rows(input_tsv)
    if not args.no_references:
        rows = with_reference_rows(rows)

    if not rows:
        raise ValueError(f"No rows found in {input_tsv}")

    tsv_path = output_dir / "contig2_plasmid_types.tsv"
    svg_path = output_dir / "contig2_plasmid_types.svg"
    fixed_svg_path = output_dir / "contig2_plasmid_types_fixed.svg"

    write_tsv(tsv_path, rows)
    write_svg(svg_path, rows)
    write_svg(fixed_svg_path, rows)

    print(f"Wrote {len(rows)} rows")
    print(f"TSV: {tsv_path}")
    print(f"SVG: {svg_path}")
    print(f"SVG copy: {fixed_svg_path}")
    return 0


if __name__ == "__main__":
    raise SystemExit(main())
```

Script 16: make_contig3_plasmid_types_colored_table.py
Source file: make_contig3_plasmid_types_colored_table.py
Line count: 224


```python
#!/usr/bin/env python3
from pathlib import Path
import argparse
import csv
import html
import re
import xml.sax.saxutils as sx


DEFAULT_SVG = Path(
    "<project_data_directory>/"
    "Salm sequencing paper 2025/Figures/
contig3_plasmid_types_fixed.svg"
)

HEADERS = ["Isolate", "Plasmid type", "Size", "Mobility", "Cluster"]
FALLBACK_COLORS = {
    "IncC": "#b8324c",
    "IncX1": "#2d7fb8",
    "IncI-gamma/K1": "#159a7d",
    "IncI1/B/O": "#d4861d",
}


def strip_tags(value):
    value = re.sub(r"<[^>]+>", "", value)
    return html.unescape(value).strip()


def extract_source_rows(svg_path):
    svg = svg_path.read_text(errors="replace")
    texts = [strip_tags(match.group(1)) for match in
re.finditer(r"<text\b[^>]*>(.*?)</text>", svg, re.S)]
    texts = [text for text in texts if text]

    try:
        start = texts.index("Cluster") + 1
    except ValueError:
        raise SystemExit("Could not find the table header 'Cluster' in
the SVG.")

    values = texts[start:]
    if len(values) % len(HEADERS) != 0:
```

```
        raise SystemExit(f"Could not split SVG text into table rows.
Found {len(values)} data values.")

    rows = []
    for i in range(0, len(values), len(HEADERS)):
        row_values = values[i : i + len(HEADERS)]
        rows.append(dict(zip(HEADERS, row_values)))

    color_matches = []
    for match in re.finditer(r"<rect\b([^>]*)>", svg):
        attrs = match.group(1)
        if 'x="230"' not in attrs or 'width="130"' not in attrs:
            continue
        fill_match = re.search(r'fill="([^"]+)"', attrs)
        if fill_match:
            color_matches.append(fill_match.group(1))

    type_colors = dict(FALLBACK_COLORS)
    for row, color in zip(rows, color_matches):
        type_colors[row["Plasmid type"]] = color

    return rows, type_colors


def write_tsv(rows, path):
    with path.open("w", newline="") as handle:
        writer = csv.DictWriter(handle, fieldnames=HEADERS,
delimiter="\t")
        writer.writeheader()
        writer.writerows(rows)


def write_html(rows, type_colors, path):
    parts = [
        "<!doctype html>",
        "<html>",
        "<head>",
        "<meta charset='utf-8'>",
        "<title>Contig 3 plasmid types colored table</title>",
        "<style>",
        "body{font-family:Arial,Helvetica,sans-
serif;margin:24px;color:#222;background:#fff}",
        "h2{font-size:20px;margin:0 0 6px 0}",
        ".note{font-size:13px;color:#555;margin:0 0 16px 0}",
        "table{border-collapse:collapse;font-size:13px}",
        "th,td{border:1px solid #cfcfcf;padding:8px 12px;text-
align:left;white-space:nowrap}",
        "th{background:#f1f3f5;font-weight:700;text-align:center}",
        "tbody tr:nth-child(odd) td{background:#fbfbfb}",
        "td.isolate{font-weight:700;background:#fafafa}",
```

```
        "td.type{color:#fff;font-weight:700;text-align:center}",
        "td.size,td.mobility{text-align:center}",
        "</style>",
        "</head>",
        "<body>",
        "<h2>Contig 3 Plasmid Types Across NCTR Isolates</h2>",
        "<p class='note'>Plasmid types based on MOB-suite replicon/
mobility classification.</p>",
        "<table>",
        "<thead><tr>",
    ]

    for header in HEADERS:
        parts.append(f"<th>{html.escape(header)}</th>")
    parts.append("</tr></thead><tbody>")

    for row in rows:
        color = type_colors.get(row["Plasmid type"], "#777777")
        parts.append("<tr>")
        parts.append(f"<td
class='isolate'>{html.escape(row['Isolate'])}</td>")
        parts.append(
            f"<td class='type' style='background:
{html.escape(color)}'>{html.escape(row['Plasmid type'])}</td>"
        )
        parts.append(f"<td class='size'>{html.escape(row['Size'])}</
td>")
        parts.append(f"<td
class='mobility'>{html.escape(row['Mobility'])}</td>")
        parts.append(f"<td>{html.escape(row['Cluster'])}</td>")
        parts.append("</tr>")

    parts.extend(["</tbody></table>", "</body>", "</html>"])
    path.write_text("\n".join(parts))


def svg_text(x, y, text, size=13, weight="400", anchor="start",
fill="#222"):
    return (
        f'<text x="{x}" y="{y}" font-family="Arial, Helvetica, sans-
serif" '
        f'font-size="{size}" font-weight="{weight}" text-
anchor="{anchor}" fill="{fill}">'
        f"{sx.escape(text)}</text>"
    )


def write_svg(rows, type_colors, path):
    width = 920
    height = 455
```

```
    margin_x = 28
    title_y = 34
    subtitle_y = 58
    table_y = 82
    header_h = 40
    row_h = 36
    table_w = width - (margin_x * 2)
    col_w = [142, 170, 128, 150, table_w - 142 - 170 - 128 - 150]
    col_x = [margin_x]
    for w in col_w[:-1]:
        col_x.append(col_x[-1] + w)

    parts = [
        f'<svg xmlns="http://www.w3.org/2000/svg" width="{width}"
height="{height}" viewBox="0 0 {width} {height}">',
        '<rect width="100%" height="100%" fill="#ffffff"/>',
        svg_text(margin_x, title_y, "Contig 3 Plasmid Types Across
NCTR Isolates", size=20, weight="700"),
        svg_text(
            margin_x,
            subtitle_y,
            "Plasmid types based on MOB-suite replicon/mobility
classification.",
            size=13,
            fill="#555",
        ),
        f'<rect x="{margin_x}" y="{table_y}" width="{table_w}"
height="{header_h}" fill="#f1f3f5" stroke="#cfcfcf"/>',
    ]

    for i, header in enumerate(HEADERS):
        x = col_x[i]
        parts.append(f'<line x1="{x}" y1="{table_y}" x2="{x}"
y2="{table_y + header_h}" stroke="#cfcfcf"/>')
        parts.append(svg_text(x + col_w[i] / 2, table_y + 25, header,
size=13, weight="700", anchor="middle"))
    parts.append(
        f'<line x1="{margin_x + table_w}" y1="{table_y}" x2="{margin_x
+ table_w}" y2="{table_y + header_h}" stroke="#cfcfcf"/>'
    )

    y = table_y + header_h
    for row_idx, row in enumerate(rows):
        row_fill = "#fbfbfb" if row_idx % 2 == 0 else "#ffffff"
        parts.append(f'<rect x="{margin_x}" y="{y}" width="{table_w}"
height="{row_h}" fill="{row_fill}" stroke="#cfcfcf"/>')
        for x in col_x:
            parts.append(f'<line x1="{x}" y1="{y}" x2="{x}" y2="{y +
row_h}" stroke="#cfcfcf"/>')
        parts.append(
```

```
            f'<line x1="{margin_x + table_w}" y1="{y}" x2="{margin_x +
table_w}" y2="{y + row_h}" stroke="#cfcfcf"/>'
        )

        type_color = type_colors.get(row["Plasmid type"], "#777777")
        parts.append(
            f'<rect x="{col_x[1]}" y="{y}" width="{col_w[1]}"
height="{row_h}" fill="{type_color}" stroke="#cfcfcf"/>'
        )
        parts.append(svg_text(col_x[0] + 12, y + 23, row["Isolate"],
size=13, weight="700"))
        parts.append(
            svg_text(
                col_x[1] + col_w[1] / 2,
                y + 23,
                row["Plasmid type"],
                size=13,
                weight="700",
                anchor="middle",
                fill="#ffffff",
            )
        )
        parts.append(svg_text(col_x[2] + col_w[2] / 2, y + 23,
row["Size"], size=13, anchor="middle"))
        parts.append(svg_text(col_x[3] + col_w[3] / 2, y + 23,
row["Mobility"], size=13, anchor="middle"))
        parts.append(svg_text(col_x[4] + 12, y + 23, row["Cluster"],
size=13))
        y += row_h

    parts.append("</svg>")
    path.write_text("\n".join(parts))


def main():
    parser = argparse.ArgumentParser(description="Convert the contig3
plasmid-type SVG into a colored table.")
    parser.add_argument("--input-svg", type=Path, default=DEFAULT_SVG,
help="Input SVG figure.")
    parser.add_argument("--out-prefix", type=Path, default=None,
help="Output prefix without extension.")
    args = parser.parse_args()

    if not args.input_svg.exists():
        raise SystemExit(f"Cannot find input SVG: {args.input_svg}")

    out_prefix = args.out_prefix
    if out_prefix is None:
        out_prefix =
args.input_svg.with_name("contig3_plasmid_types_colored_table")
```

```
    rows, type_colors = extract_source_rows(args.input_svg)
    write_tsv(rows, out_prefix.with_suffix(".tsv"))
    write_html(rows, type_colors, out_prefix.with_suffix(".html"))
    write_svg(rows, type_colors, out_prefix.with_suffix(".svg"))

    print(f"Rows extracted: {len(rows)}")
    print("Saved:")
    print(out_prefix.with_suffix(".tsv"))
    print(out_prefix.with_suffix(".html"))
    print(out_prefix.with_suffix(".svg"))


if __name__ == "__main__":
    main()
```


Script 17: build_contig3_plasmid_types_word_table.py
Source file: build_contig3_plasmid_types_word_table.py
Line count: 194


```python
#!/usr/bin/env python3
from pathlib import Path
import argparse
import csv

from docx import Document
from docx.enum.table import WD_CELL_VERTICAL_ALIGNMENT
from docx.enum.text import WD_ALIGN_PARAGRAPH
from docx.oxml import OxmlElement
from docx.oxml.ns import qn
from docx.shared import Inches, Pt


TYPE_COLORS = {
    "IncC": "B8324C",
    "IncX1": "2D7FB8",
    "IncI-gamma/K1": "159A7D",
    "IncI1/B/O": "D4861D",
}


def set_cell_shading(cell, fill):
    tc_pr = cell._tc.get_or_add_tcPr()
    shd = tc_pr.find(qn("w:shd"))
    if shd is None:
```

```
        shd = OxmlElement("w:shd")
        tc_pr.append(shd)
    shd.set(qn("w:fill"), fill)


def set_cell_text(cell, text, bold=False, color="000000", size=9.5,
align=WD_ALIGN_PARAGRAPH.CENTER):
    cell.text = ""
    paragraph = cell.paragraphs[0]
    paragraph.alignment = align
    run = paragraph.add_run(text)
    run.bold = bold
    run.font.name = "Arial"
    run._element.rPr.rFonts.set(qn("w:eastAsia"), "Arial")
    run.font.size = Pt(size)
    run.font.color.rgb = None
    color_element = run._element.get_or_add_rPr().find(qn("w:color"))
    if color_element is None:
        color_element = OxmlElement("w:color")
        run._element.get_or_add_rPr().append(color_element)
    color_element.set(qn("w:val"), color)
    cell.vertical_alignment = WD_CELL_VERTICAL_ALIGNMENT.CENTER


def set_cell_width(cell, width_inches):
    tc = cell._tc
    tc_pr = tc.get_or_add_tcPr()
    tc_w = tc_pr.find(qn("w:tcW"))
    if tc_w is None:
        tc_w = OxmlElement("w:tcW")
        tc_pr.append(tc_w)
    tc_w.set(qn("w:w"), str(int(width_inches * 1440)))
    tc_w.set(qn("w:type"), "dxa")


def set_cell_margins(cell, top=80, start=100, bottom=80, end=100):
    tc = cell._tc
    tc_pr = tc.get_or_add_tcPr()
    tc_mar = tc_pr.find(qn("w:tcMar"))
    if tc_mar is None:
        tc_mar = OxmlElement("w:tcMar")
        tc_pr.append(tc_mar)
    for margin_name, value in [("top", top), ("start", start),
("bottom", bottom), ("end", end)]:
        element = tc_mar.find(qn(f"w:{margin_name}"))
        if element is None:
            element = OxmlElement(f"w:{margin_name}")
            tc_mar.append(element)
        element.set(qn("w:w"), str(value))
        element.set(qn("w:type"), "dxa")
```

```
def set_cell_borders(cell, color="000000", size="8"):
    tc_pr = cell._tc.get_or_add_tcPr()
    tc_borders = tc_pr.find(qn("w:tcBorders"))
    if tc_borders is None:
        tc_borders = OxmlElement("w:tcBorders")
        tc_pr.append(tc_borders)

    for edge in ("top", "left", "bottom", "right", "insideH",
"insideV"):
        tag = qn(f"w:{edge}")
        element = tc_borders.find(tag)
        if element is None:
            element = OxmlElement(f"w:{edge}")
            tc_borders.append(element)
        element.set(qn("w:val"), "single")
        element.set(qn("w:sz"), size)
        element.set(qn("w:space"), "0")
        element.set(qn("w:color"), color)


def set_table_width(table, width_inches):
    tbl_pr = table._tbl.tblPr
    tbl_w = tbl_pr.find(qn("w:tblW"))
    if tbl_w is None:
        tbl_w = OxmlElement("w:tblW")
        tbl_pr.append(tbl_w)
    tbl_w.set(qn("w:w"), str(int(width_inches * 1440)))
    tbl_w.set(qn("w:type"), "dxa")


def load_rows(tsv_path):
    with tsv_path.open(newline="") as handle:
        return list(csv.DictReader(handle, delimiter="\t"))


def build_docx(rows, output_docx):
    doc = Document()
    section = doc.sections[0]
    section.top_margin = Inches(0.8)
    section.bottom_margin = Inches(0.8)
    section.left_margin = Inches(0.75)
    section.right_margin = Inches(0.75)

    styles = doc.styles
    styles["Normal"].font.name = "Arial"
    styles["Normal"]._element.rPr.rFonts.set(qn("w:eastAsia"),
"Arial")
    styles["Normal"].font.size = Pt(10)
```

```
    title = doc.add_paragraph()
    title.alignment = WD_ALIGN_PARAGRAPH.LEFT
    run = title.add_run("Table X. Contig 3 plasmid types across NCTR
isolates")
    run.bold = True
    run.font.name = "Arial"
    run._element.rPr.rFonts.set(qn("w:eastAsia"), "Arial")
    run.font.size = Pt(11)

    note = doc.add_paragraph()
    note.alignment = WD_ALIGN_PARAGRAPH.LEFT
    note_run = note.add_run("Plasmid types are based on MOB-suite
replicon/mobility classification.")
    note_run.font.name = "Arial"
    note_run._element.rPr.rFonts.set(qn("w:eastAsia"), "Arial")
    note_run.font.size = Pt(9)
    note.paragraph_format.space_after = Pt(6)

    headers = ["Isolate", "Plasmid type", "Size", "Mobility",
"Cluster"]
    widths = [1.15, 1.55, 1.15, 1.25, 1.85]
    table = doc.add_table(rows=1, cols=len(headers))
    table.style = "Table Grid"
    table.autofit = False
    set_table_width(table, sum(widths))

    for idx, header in enumerate(headers):
        cell = table.rows[0].cells[idx]
        set_cell_width(cell, widths[idx])
        set_cell_margins(cell)
        set_cell_shading(cell, "EDEFF2")
        set_cell_borders(cell, color="000000")
        set_cell_text(cell, header, bold=True, size=9.5)

    for row in rows:
        cells = table.add_row().cells
        values = [
            row["Isolate"],
            row["Plasmid type"],
            row["Size"],
            row["Mobility"],
            row["Cluster"],
        ]
        for idx, value in enumerate(values):
            cell = cells[idx]
            set_cell_width(cell, widths[idx])
            set_cell_margins(cell)
            if idx == 1:
                fill = TYPE_COLORS.get(value, "777777")
```

```
                set_cell_shading(cell, fill)
                set_cell_borders(cell, color=fill)
                set_cell_text(cell, value, bold=True, color="FFFFFF",
size=9.5)
            else:
                set_cell_shading(cell, "FFFFFF")
                set_cell_borders(cell, color="000000")
                align = WD_ALIGN_PARAGRAPH.LEFT if idx in {0, 4} else
WD_ALIGN_PARAGRAPH.CENTER
                set_cell_text(cell, value, bold=(idx == 0), size=9.5,
align=align)

    output_docx.parent.mkdir(parents=True, exist_ok=True)
    doc.save(output_docx)


def main():
    parser = argparse.ArgumentParser(description="Build an editable
Word table from the contig3 plasmid type TSV.")
    parser.add_argument("--input-tsv", type=Path, required=True)
    parser.add_argument("--output-docx", type=Path, required=True)
    args = parser.parse_args()

    rows = load_rows(args.input_tsv)
    build_docx(rows, args.output_docx)
    print(f"Wrote: {args.output_docx}")


if __name__ == "__main__":
    main()
```

##
##################
E. AMR and metal-resistance summary tables/heatmaps
##
##################

Script 18: make_contig3_amr_heatmap_and_table.py
Source file: make_contig3_amr_heatmap_and_table.py
Line count: 500


```python
#!/usr/bin/env python3
"""
Scan NCTR contig3 GenBank annotations for AMR-associated genes and
make:
```

```
  - presence/absence TSV matrix
  - hit/evidence TSV table
  - SVG heatmap
  - HTML colored table

This is annotation-based. For database-grade AMR calling, run
AMRFinderPlus,
ABRicate/ResFinder, or RGI separately and use those calls as
confirmation.
"""

from __future__ import annotations

import argparse
import csv
import glob
import html
import re
from dataclasses import dataclass
from pathlib import Path


PROJECT_DIR = Path("<project_directory>")

AMR_GENE_RE = re.compile(
    r"^(bla|tet|sul|dfr|dhfr|aad|aac|aph|ant|str|cat|cml|flo|qnr|oqx|
mcr|mph|erm|lnu|arr|fos|rmt|armA|npmA|qac|ble|bcr|ere)",
    re.I,
)

EXCLUDE_GENE_RE = re.compile(r"^(pco|sil|cop|ter|ars|mer|cus|czc)",
re.I)
EXCLUDE_PRODUCT_WORDS = [
    "copper",
    "silver",
    "tellurium",
    "tellurite",
    "arsenic",
    "mercury",
]

AMR_PRODUCT_WORDS = [
    "beta-lactamase",
    "new delhi metallo",
    "bleomycin resistance",
    "tetracycline resistance",
    "tetracycline efflux",
    "tetracycline repressor",
    "sulfonamide resistance",
    "dihydropteroate synthase",
```

```
    "trimethoprim-resistant",
    "dihydrofolate reductase",
    "aminoglycoside",
    "adenylyltransferase",
    "nucleotidyltransferase",
    "phosphotransferase",
    "16s rrna methyl",
    "streptomycin",
    "spectinomycin",
    "chloramphenicol",
    "florfenicol",
    "phenicol",
    "quinolone resistance",
    "macrolide",
    "erythromycin esterase",
    "rifampicin adp-ribosyl",
    "rifamycin adp-ribosyl",
    "fosfomycin resistance",
    "colistin resistance",
    "bicyclomycin resistance",
    "quaternary ammonium compound",
    "multidrug efflux",
]

CLASS_ORDER = [
    "beta-lactam",
    "aminoglycoside",
    "trimethoprim",
    "sulfonamide",
    "tetracycline",
    "phenicol",
    "macrolide",
    "rifamycin",
    "bleomycin",
    "bicyclomycin",
    "quinolone",
    "colistin",
    "qac/efflux",
    "other",
]

COLORS = {
    "beta-lactam": "#b8324c",
    "aminoglycoside": "#2d7fb8",
    "trimethoprim": "#9a6f2d",
    "sulfonamide": "#568b2f",
    "tetracycline": "#d4861d",
    "phenicol": "#5f3b9a",
    "macrolide": "#c4517d",
    "rifamycin": "#8a6a35",
```

```
    "bleomycin": "#7d5fb2",
    "bicyclomycin": "#159a7d",
    "quinolone": "#4669b2",
    "colistin": "#8b3f2f",
    "qac/efflux": "#6b6b6b",
    "other": "#4f7f6f",
    "absent": "#eeeeee",
}


@dataclass(frozen=True)
class Hit:
    sample: str
    gene: str
    gene_class: str
    location: str
    source_gene: str
    product: str
    note: str
    matched_text: str
    source_file: Path


def parse_args() -> argparse.Namespace:
    parser = argparse.ArgumentParser(
        description="Make an annotation-based AMR heatmap/colored
table for contig3 GenBank files."
    )
    parser.add_argument(
        "--gbk-glob",
        default=str(PROJECT_DIR / "NCTR*_contig3.gbk"),
        help="Input GenBank glob. Default: NCTR contig3 GenBank files
in the project root.",
    )
    parser.add_argument(
        "--outdir",
        type=Path,
        default=PROJECT_DIR / "contig3_amr_results",
        help="Output directory.",
    )
    parser.add_argument(
        "--prefix",
        default="contig3_amr",
        help="Output file prefix.",
    )
    parser.add_argument(
        "--title",
        default="AMR genes detected in NCTR contig3 plasmid
sequences",
        help="Figure/table title.",
```

```
    )
    return parser.parse_args()


def sample_name(path: Path) -> str:
    stem = path.stem
    stem = re.sub(r"_contig[_-]?3$", "", stem, flags=re.I)
    return stem


def clean_text(value: str) -> str:
    return re.sub(r"\s+", " ", value.replace("\n", " ")).strip()


def parse_cds_blocks(path: Path) -> list[tuple[str, str]]:
    blocks: list[tuple[str, str]] = []
    current: list[str] = []
    location = ""
    in_cds = False

    for line in path.read_text(errors="replace").splitlines():
        if line.startswith("     CDS"):
            if current:
                blocks.append((location, "\n".join(current)))
            current = [line]
            location = line[21:].strip()
            in_cds = True
            continue

        if in_cds and re.match(r"^     \S", line) and not
line.startswith("                     "):
            blocks.append((location, "\n".join(current)))
            current = []
            location = ""
            in_cds = False
            continue

        if in_cds:
            current.append(line)

    if current:
        blocks.append((location, "\n".join(current)))
    return blocks


def qualifier_values(block: str, key: str) -> list[str]:
    values = re.findall(rf'/{re.escape(key)}="([^"]*)"', block,
flags=re.S)
    return [clean_text(value) for value in values]
```

```
def normalize_label(label: str) -> str:
    label = clean_text(label)
    label = label.replace("bla ", "bla")
    label = re.sub(r"\bbla[-_ ]?(TEM|CMY|NDM|SHV|OXA)[-_ ]?([A-Za-
z0-9]*)\b", lambda m: "bla" + m.group(1).upper() + (("-" + m.group(2))
if m.group(2) else ""), label, flags=re.I)
    label = re.sub(r"\bCMY[-_ ]?(\d+)\b", r"blaCMY-\1", label,
flags=re.I)
    label = re.sub(r"\bNDM[-_ ]?(\d+)\b", r"blaNDM-\1", label,
flags=re.I)
    label = re.sub(r"\bCmlA(\d+)\b", r"cmlA\1", label, flags=re.I)
    label = re.sub(r"\s+", "", label)
    label = re.sub(r"_(\d+)$", "", label)
    label = re.sub(r"^dhfrI$", "dfrA1/dhfrI", label, flags=re.I)
    if label.lower() == "tap":
        label = "tap"
    return label


def infer_label(gene_values: list[str], product_note: str) -> str:
    beta_match = re.search(
        r"\bbeta[- ]lactamase\s+(TEM|CMY|NDM|SHV|OXA)[-_ ]?([A-Za-
z0-9]*)\b",
        product_note,
        flags=re.I,
    )
    if beta_match:
        family = beta_match.group(1).upper()
        suffix = beta_match.group(2)
        return "bla" + family + (("-" + suffix) if suffix else "")

    patterns = [
        r"\bbla[-_ ]?(?:TEM|CMY|NDM|SHV|OXA)[-_ ]?[A-Za-z0-9]*\b",
        r"\b(?:CMY|NDM|TEM|SHV|OXA)[-_ ]?\d+\b",
        r"\btet\([A-Za-z0-9]+\)\b",
        r"\btet[A-Z0-9]{1,3}\b",
        r"\bsul[123](?:_\d+)?\b",
        r"\bdhfrI\b",
        r"\bdfrA\d+\b",
        r"\baadA[A-Za-z0-9_-]*\b",
        r"\baadB\b",
        r"\baac\([^)]+\)-[A-Za-z0-9]+",
        r"\baph\([^)]+\)-[A-Za-z0-9]+",
        r"\bant\([^)]+\)-[A-Za-z0-9]+",
        r"\bneo\b",
        r"\brmtB\b",
        r"\bfloR\b",
        r"\bcat[A-Za-z0-9_-]*\b",
        r"\bcmlA\d+\b",
```

```
        r"\bqnr[A-Za-z0-9]+\b",
        r"\bmcr-?\d+\b",
        r"\bereC\b",
        r"\barr-?3\b",
        r"\bbleMBL\b",
        r"\bbcr\b",
        r"\bqac[A-Za-z0-9_-]*\b",
        r"\bmdtL\b",
        r"\btap\b",
    ]
    for pattern in patterns:
        match = re.search(pattern, product_note, flags=re.I)
        if match:
            return normalize_label(match.group(0))

    for gene in gene_values:
        if gene and AMR_GENE_RE.search(gene) and not
EXCLUDE_GENE_RE.search(gene):
            return normalize_label(gene)

    low = product_note.lower()
    if "new delhi metallo" in low:
        return "blaNDM-like"
    if "aminoglycoside" in low and "adenylyltransferase" in low:
        return "aadA-like"
    if "aminoglycoside" in low and "nucleotidyltransferase" in low:
        return "aad-like"
    if "aminoglycoside" in low and "phosphotransferase" in low:
        return "aph-like"
    if "dihydrofolate reductase" in low:
        return "dfrA1/dhfrI"
    if "16s rrna methyl" in low:
        return "rmt-like"
    if "erythromycin esterase" in low:
        return "ere-like"
    if "rifampicin adp-ribosyl" in low or "rifamycin adp-ribosyl" in
low:
        return "arr-like"
    if "bicyclomycin resistance" in low:
        return "bcr"
    if "bleomycin resistance" in low:
        return "ble-like"
    return "unclassified_AMR"


def gene_class(label: str, product_note: str) -> str:
    low_label = label.lower()
    low_text = product_note.lower()
    if low_label.startswith("bla") or "beta-lactamase" in low_text or
"new delhi metallo" in low_text:
```

```
        return "beta-lactam"
    if low_label.startswith(("aad", "aac", "aph", "ant", "str", "rmt",
"neo")) or "aminoglycoside" in low_text or "16s rrna methyl" in
low_text:
        return "aminoglycoside"
    if low_label.startswith(("dfr", "dhfr")) or "dihydrofolate
reductase" in low_text or "trimethoprim" in low_text:
        return "trimethoprim"
    if low_label.startswith("sul") or "sulfonamide" in low_text or
"dihydropteroate" in low_text:
        return "sulfonamide"
    if low_label.startswith("tet") or "tetracycline" in low_text:
        return "tetracycline"
    if low_label.startswith(("cml", "cat", "flo")) or
"chloramphenicol" in low_text or "florfenicol" in low_text or
"phenicol" in low_text:
        return "phenicol"
    if low_label.startswith(("erm", "mph", "lnu", "ere")) or
"macrolide" in low_text or "erythromycin" in low_text:
        return "macrolide"
    if low_label.startswith("arr") or "rifampicin" in low_text or
"rifamycin" in low_text:
        return "rifamycin"
    if low_label.startswith("ble") or "bleomycin" in low_text:
        return "bleomycin"
    if low_label.startswith("bcr") or "bicyclomycin" in low_text:
        return "bicyclomycin"
    if low_label.startswith(("qnr", "oqx")) or "quinolone" in
low_text:
        return "quinolone"
    if low_label.startswith("mcr") or "colistin" in low_text:
        return "colistin"
    if low_label.startswith(("qac", "mdt", "tap")) or "quaternary
ammonium" in low_text or "efflux" in low_text:
        return "qac/efflux"
    return "other"


def is_amr(gene_values: list[str], product_note: str) -> bool:
    low = product_note.lower()
    if any(word in low for word in EXCLUDE_PRODUCT_WORDS):
        return False
    for gene in gene_values:
        if EXCLUDE_GENE_RE.search(gene):
            return False
        if AMR_GENE_RE.search(gene):
            return True
    return any(word in low for word in AMR_PRODUCT_WORDS)
```

```
def find_hits(sample: str, path: Path) -> list[Hit]:
    hits: list[Hit] = []
    seen: set[tuple[str, str]] = set()
    for location, block in parse_cds_blocks(path):
        genes = qualifier_values(block, "gene")
        products = qualifier_values(block, "product")
        notes = qualifier_values(block, "note") +
qualifier_values(block, "function")
        product_note = clean_text(" | ".join(products + notes))
        if not is_amr(genes, product_note):
            continue
        label = infer_label(genes, product_note)
        key = (label, location)
        if key in seen:
            continue
        seen.add(key)
        hits.append(
            Hit(
                sample=sample,
                gene=label,
                gene_class=gene_class(label, product_note),
                location=location,
                source_gene=";".join(genes) if genes else "-",
                product=";".join(products) if products else "-",
                note=";".join(notes) if notes else "-",
                matched_text=product_note,
                source_file=path,
            )
        )
    return hits


def sort_gene(label: str, classes: dict[str, str]) -> tuple[int, str]:
    cls = classes.get(label, "other")
    return (CLASS_ORDER.index(cls) if cls in CLASS_ORDER else 99,
label.lower())


def write_matrix(path: Path, samples: list[str], genes: list[str],
present: dict[str, set[str]]) -> None:
    with path.open("w", newline="") as handle:
        writer = csv.writer(handle, delimiter="\t")
        writer.writerow(["isolate"] + genes)
        for sample in samples:
            writer.writerow([sample] + ["present" if gene in
present[sample] else "absent" for gene in genes])


def write_hits(path: Path, hits: list[Hit]) -> None:
    with path.open("w", newline="") as handle:
```

```
        writer = csv.writer(handle, delimiter="\t")
        writer.writerow(["isolate", "amr_gene", "class", "location", 
"gene_annotation", "product", "note", "matched_text", "source_file"])
        for hit in hits:
            writer.writerow([hit.sample, hit.gene, hit.gene_class, 
hit.location, hit.source_gene, hit.product, hit.note, 
hit.matched_text, str(hit.source_file)])


def write_svg(path: Path, title: str, samples: list[str], genes: 
list[str], classes: dict[str, str], present: dict[str, set[str]]) -> 
None:
    cell_w, row_h = 36, 30
    left, top, right, bottom = 165, 145, 70, 130
    width = max(700, left + len(genes) * cell_w + right)
    height = top + len(samples) * row_h + bottom
    parts = [
        f'<svg xmlns="http://www.w3.org/2000/svg" width="{width}" 
height="{height}" viewBox="0 0 {width} {height}">',
        '<rect width="100%" height="100%" fill="white"/>',
        '<style>text{font-family:Arial,Helvetica,sans-
serif}.title{font-size:18px;font-weight:700}.small{font-
size:11px}.gene{font-size:11px}.sample{font-size:12px}</style>',
        f'<text x="24" y="34" class="title">{html.escape(title)}</
text>',
        '<text x="24" y="56" class="small" fill="#444">Filled cells 
indicate AMR-associated CDS annotations detected from gene/product/
note fields.</text>',
    ]
    if not genes:
        parts.append('<text x="24" y="95" class="sample">No AMR-
associated annotations detected.</text>')
        parts.append("</svg>")
        path.write_text("\n".join(parts))
        return
    for j, gene in enumerate(genes):
        x = left + j * cell_w + cell_w / 2
        parts.append(f'<g transform="translate({x:.1f},{top - 14}) 
rotate(-55)"><text class="gene" text-
anchor="start">{html.escape(gene)}</text></g>')
        parts.append(f'<rect x="{left + j * cell_w}" y="{top - 112}" 
width="{cell_w}" height="8" fill="{COLORS[classes[gene]]}"/>')
    for i, sample in enumerate(samples):
        y = top + i * row_h
        parts.append(f'<text x="{left - 10}" y="{y + 20}" 
class="sample" text-anchor="end">{html.escape(sample)}</text>')
        for j, gene in enumerate(genes):
            x = left + j * cell_w
            hit = gene in present[sample]
            fill = COLORS[classes[gene]] if hit else COLORS["absent"]
```

```
            stroke = "#777" if hit else "#cfcfcf"
            parts.append(f'<rect x="{x}" y="{y}" width="{cell_w - 2}"
height="{row_h - 3}" rx="2" fill="{fill}" stroke="{stroke}" stroke-
width="0.7"/>')
            if hit:
                parts.append(f'<text x="{x + (cell_w - 2) / 2}" y="{y
+ 19}" class="small" text-anchor="middle" fill="white">+</text>')
    legend_y = top + len(samples) * row_h + 38
    legend_x = 24
    for cls in CLASS_ORDER:
        if cls not in set(classes.values()):
            continue
        parts.append(f'<rect x="{legend_x}" y="{legend_y - 14}"
width="14" height="14" fill="{COLORS[cls]}"/>')
        parts.append(f'<text x="{legend_x + 20}" y="{legend_y - 3}"
class="small">{html.escape(cls)}</text>')
        legend_x += max(105, len(cls) * 8 + 40)
    parts.append(f'<rect x="24" y="{legend_y + 24}" width="14"
height="14" fill="{COLORS["absent"]}" stroke="#cfcfcf"/>')
    parts.append(f'<text x="44" y="{legend_y + 35}"
class="small">absent/not detected</text>')
    parts.append("</svg>")
    path.write_text("\n".join(parts))


def write_html(path: Path, title: str, samples: list[str], genes:
list[str], classes: dict[str, str], present: dict[str, set[str]]) ->
None:
    rows = [
        "<!doctype html>",
        "<meta charset='utf-8'>",
        f"<title>{html.escape(title)}</title>",
        "<style>body{font-family:Arial,Helvetica,sans-
serif;margin:18px;color:#222}h2{font-size:18px;margin:0 0 8px}p{font-
size:12px;margin:0 0 12px}.figure-wrap{display:flex;align-items:flex-
start;gap:16px;width:max-content}table{border-collapse:collapse}
th,td{border:1px solid #d0d0d0;padding:4px 6px;text-align:center;font-
size:12px}th.gene{writing-mode:vertical-
rl;transform:rotate(180deg);height:112px;min-width:24px}
td.sample{text-align:right;font-weight:600;background:#fafafa;white-
space:nowrap}.absent{background:#eee;color:#777}.legend{display:flex;f
lex-direction:column;gap:7px;margin:0;padding:10px 12px;border:1px
solid #d0d0d0;background:#fafafa;min-width:142px;font-
size:12px}.legend-title{font-weight:700;margin-
bottom:3px}.swatch{display:inline-block;width:14px;height:14px;margin-
right:7px;vertical-align:-2px;border:1px solid #aaa}@media
print{body{margin:8px}.figure-wrap{gap:10px}th,td{padding:3px
5px;font-size:11px}.legend{font-size:11px;min-width:126px}}</style>",
        f"<h2>{html.escape(title)}</h2>",
        "<p>Filled cells indicate AMR-associated CDS annotations
```

```
detected from GenBank gene/product/note fields.</p>",
        "<div class='figure-wrap'>",
        "<table><thead><tr><th>isolate</th>",
    ]
    for gene in genes:
        rows.append(f"<th class='gene'>{html.escape(gene)}</th>")
    rows.append("</tr></thead><tbody>")
    for sample in samples:
        rows.append(f"<tr><td class='sample'>{html.escape(sample)}</
td>")
        for gene in genes:
            if gene in present[sample]:
                color = COLORS[classes[gene]]
                rows.append(f"<td style='background:
{color};color:white;font-weight:700'>+</td>")
            else:
                rows.append("<td class='absent'>-</td>")
        rows.append("</tr>")
    rows.append("</tbody></table>")
    rows.append("<div class='legend'><div class='legend-
title'>Antimicrobial class</div>")
    for cls in CLASS_ORDER:
        if cls in set(classes.values()):
            rows.append(f"<span><span class='swatch'
style='background:{COLORS[cls]}'></span>{html.escape(cls)}</span>")
    rows.extend(["<span><span class='swatch'
style='background:#eee'></span>absent/not detected</span>", "</div>",
"</div>"])
    path.write_text("\n".join(rows))


def main() -> int:
    args = parse_args()
    paths = [Path(path) for path in sorted(glob.glob(args.gbk_glob))]
    if not paths:
        raise SystemExit(f"No GenBank files found for glob:
{args.gbk_glob}")

    sample_hits: dict[str, list[Hit]] = {}
    all_hits: list[Hit] = []
    for path in paths:
        sample = sample_name(path)
        hits = find_hits(sample, path)
        sample_hits[sample] = hits
        all_hits.extend(hits)

    samples = list(sample_hits)
    class_by_gene: dict[str, str] = {}
    for hit in all_hits:
        class_by_gene.setdefault(hit.gene, hit.gene_class)
```

```python
    genes = sorted(class_by_gene, key=lambda gene: sort_gene(gene, 
class_by_gene))
    present = {sample: {hit.gene for hit in hits} for sample, hits in 
sample_hits.items()}

    args.outdir.mkdir(parents=True, exist_ok=True)
    matrix_path = args.outdir / f"{args.prefix}_presence_absence.tsv"
    hits_path = args.outdir / f"{args.prefix}_hits.tsv"
    svg_path = args.outdir / f"{args.prefix}_heatmap.svg"
    html_path = args.outdir / f"{args.prefix}_colored_table.html"

    write_matrix(matrix_path, samples, genes, present)
    write_hits(hits_path, all_hits)
    write_svg(svg_path, args.title, samples, genes, class_by_gene, 
present)
    write_html(html_path, args.title, samples, genes, class_by_gene, 
present)

    print(f"Samples scanned: {len(samples)}")
    print(f"AMR genes detected: {len(genes)}")
    print("Outputs:")
    print(f"  {matrix_path}")
    print(f"  {hits_path}")
    print(f"  {svg_path}")
    print(f"  {html_path}")
    return 0


if __name__ == "__main__":
    raise SystemExit(main())
```


Script 19: make_pndm_contig3_amr_heatmap.py
Source file: make_pndm_contig3_amr_heatmap.py
Line count: 643


```python
#!/usr/bin/env python3
"""
Regenerate an annotation-based AMR heatmap for pNDM and NCTR contig3 
plasmids.

Outputs:
  - <prefix>_matrix.tsv: 0/1 presence-absence matrix
  - <prefix>_hits.tsv: evidence table showing matched GenBank 
annotations
  - <prefix>_heatmap.svg: heatmap figure
```

```
This script scans GenBank CDS annotations only. It recognizes gene
names plus
product/note annotations such as "NDM-1", "CMY-6", "RmtB", "Sul1", and
"CmlA7",
which were missed by the older heatmap script.
"""

from __future__ import annotations

import argparse
import csv
import html
import re
import sys
from dataclasses import dataclass
from pathlib import Path


PROJECT_DIR = Path("<project_directory>")
OUT_DIR = Path("<output_directory>/amr_gene_comparison")


@dataclass(frozen=True)
class Target:
    label: str
    gene_class: str
    patterns: tuple[str, ...]


@dataclass
class Feature:
    key: str
    location: str
    qualifiers: dict[str, list[str]]


@dataclass
class Hit:
    sample: str
    target: str
    gene_class: str
    location: str
    gene: str
    product: str
    note: str
    matched_text: str
    source_file: Path
```

```
TARGETS = [
    Target(
        "blaTEM",
        "beta-lactam",
        (
            r"\bbla[-_ ]?TEM[A-Z0-9_-]*\b",
            r"\bTEM[-_ ]?\d*\b.*\bbeta[- ]lactamase\b",
            r"\bbeta[- ]lactamase\s+TEM[-_ ]?\d*\b",
        ),
    ),
    Target(
        "blaCMY-2",
        "beta-lactam",
        (
            r"\bbla[-_ ]?CMY[-_ ]?2\b",
            r"\bCMY[-_ ]?2\b",
            r"\bbeta[- ]lactamase\s+CMY[-_ ]?2\b",
        ),
    ),
    Target(
        "blaCMY-6",
        "beta-lactam",
        (
            r"\bbla[-_ ]?CMY[-_ ]?6\b",
            r"\bCMY[-_ ]?6\b",
            r"\bbeta[- ]lactamase\s+CMY[-_ ]?6\b",
        ),
    ),
    Target(
        "blaNDM-1",
        "beta-lactam",
        (
            r"\bbla[-_ ]?NDM[-_ ]?1\b",
            r"\bNDM[-_ ]?1\b",
            r"\bNew Delhi metallo[- ]beta[- ]lactamase\b",
        ),
    ),
    Target(
        "bleMBL",
        "bleomycin",
        (
            r"\bbleMBL\b",
            r"\bbleomycin resistance\b",
        ),
    ),
    Target(
        "aadA-like",
        "aminoglycoside",
        (
            r"\baadA[A-Z0-9_-]*\b",
```

```
            r"\bAadA[A-Z0-9_-]*\b",
            r"\baminoglycoside.*adenylyltransferase\b",
        ),
    ),
    Target(
        "aadB",
        "aminoglycoside",
        (
            r"\baadB\b",
            r"\b2['\"]?-aminoglycoside nucleotidyltransferase\b",
            r"\baminoglycoside.*nucleotidyltransferase\b",
        ),
    ),
    Target(
        "neo/aph(3')",
        "aminoglycoside",
        (
            r"\bneo[A-Z0-9_-]*\b",
            r"\baph\(3['\"]?\)[A-Z0-9_-]*\b",
            r"\baminoglycoside\s+3['\"]?-phosphotransferase\b",
        ),
    ),
    Target(
        "rmtB",
        "aminoglycoside",
        (
            r"\brmtB\b",
            r"\b16S rRNA methylase\b",
            r"\b16S rRNA methyltransferase\b",
        ),
    ),
    Target(
        "sul1",
        "sulfonamide",
        (
            r"\bsul1\b",
            r"\bSul1\b",
        ),
    ),
    Target(
        "sul2",
        "sulfonamide",
        (
            r"\bsul2\b",
            r"\bSul2\b",
            r"\bdihydropteroate synthase type[- ]?2\b",
        ),
    ),
    Target(
        "tetA",
```

```
        "tetracycline",
        (
            r"\btetA\b",
            r"\btetracycline resistance protein,\s*class [AC]\b",
        ),
    ),
    Target(
        "tetR",
        "tetracycline",
        (
            r"\btetR\b",
            r"\btetracycline repressor\b",
        ),
    ),
    Target(
        "tet(M)",
        "tetracycline",
        (
            r"\btet\(M\)\b",
            r"\btetM\b",
            r"\btetracycline resistance ribosomal protection\b",
        ),
    ),
    Target(
        "cmlA5",
        "phenicol",
        (
            r"\bcmlA5\b",
            r"\bCmlA5\b",
        ),
    ),
    Target(
        "cmlA7",
        "phenicol",
        (
            r"\bcmlA7\b",
            r"\bCmlA7\b",
        ),
    ),
    Target(
        "ereC",
        "macrolide",
        (
            r"\bereC\b",
            r"\bEreC\b",
            r"\berythromycin esterase\b",
        ),
    ),
    Target(
        "arr3",
```

```
        "rifamycin",
        (
            r"\barr3\b",
            r"\brifampicin ADP-ribosyl\w* transferase\b",
            r"\brifamycin ADP-ribosyl\w* transferase\b",
        ),
    ),
    Target(
        "bcr",
        "bicyclomycin",
        (
            r"\bbcr\b",
            r"\bbicyclomycin resistance\b",
        ),
    ),
    Target(
        "mdtL",
        "multidrug efflux",
        (
            r"\bmdtL\b",
            r"\bMdtL\b",
        ),
    ),
    Target(
        "tap",
        "multidrug efflux",
        (
            r"\btap\b",
            r"\bTap\b",
        ),
    ),
]


COLORS = {
    "beta-lactam": "#b8324c",
    "bleomycin": "#7d5fb2",
    "aminoglycoside": "#2d7fb8",
    "sulfonamide": "#568b2f",
    "tetracycline": "#d4861d",
    "phenicol": "#5f3b9a",
    "macrolide": "#c4517d",
    "rifamycin": "#8a6a35",
    "bicyclomycin": "#159a7d",
    "multidrug efflux": "#6b6b6b",
}


def parse_args() -> argparse.Namespace:
    parser = argparse.ArgumentParser(
```

```
        description="Make a corrected AMR heatmap for pNDM and NCTR
contig3 GenBank files."
    )
    parser.add_argument("--project-dir", type=Path,
default=PROJECT_DIR)
    parser.add_argument("--outdir", type=Path, default=OUT_DIR)
    parser.add_argument("--prefix",
default="pNDM_contig3_amr_corrected")
    parser.add_argument(
        "--pndm",
        type=Path,
        default=None,
        help="pNDM GenBank file. Default: auto-detect common pNDM
paths.",
    )
    parser.add_argument(
        "--include-psh111",
        action="store_true",
        help="Also include pSH111_227 as the first reference row.",
    )
    parser.add_argument(
        "--show-empty-columns",
        action="store_true",
        help="Keep AMR target columns even if no sample has a hit.",
    )
    return parser.parse_args()


def first_existing(paths: list[Path], label: str) -> Path:
    for path in paths:
        if path.exists():
            return path
    raise FileNotFoundError(
        f"Could not find {label}. Tried:\n" + "\n".join(str(path) for
path in paths)
    )


def sample_paths(project_dir: Path, pndm_path: Path | None,
include_psh111: bool) -> list[tuple[str, Path]]:
    pndm = pndm_path or first_existing(
        [
            project_dir / " pNDM.gbk",
            project_dir / "pNDM.gbk",
            project_dir / "clinker with plasmids" / " pNDM.gbk",
            Path("<reference_genbank_directory>/pNDM-KN.gb"),
        ],
        "pNDM GenBank file",
    )
```

```
    samples: list[tuple[str, Path]] = []
    if include_psh111:
        samples.append(
            (
                "pSH111_227",
                first_existing(
                    [
                        project_dir / "pSH111_227.gbk",
                        project_dir / "pSH111_227.gb",
                        project_dir / "all_contigs" / "pSH111_227.gb",
                    ],
                    "pSH111_227 GenBank file",
                ),
            )
        )

    samples.append(("pNDM", pndm))

    for isolate in ["117", "122", "136", "137", "718", "719", "830",
"938"]:
        sample = f"NCTR{isolate}_contig3"
        samples.append(
            (
                sample,
                first_existing(
                    [
                        project_dir / "all_contigs" / f"{sample}.gbk",
                        project_dir / f"{sample}.gbk",
                        project_dir / "gbk" / f"{sample}_prokka" /
f"{sample}.gbk",
                        project_dir / "gbk" / "all_contigs" /
f"{sample}.gbk",
                    ],
                    sample,
                ),
            )
        )
    return samples


def parse_qualifier(line: str) -> tuple[str, str] | None:
    match = re.match(r'\s*/([^=\s]+)(?:=(.*))?$', line)
    if not match:
        return None
    key = match.group(1)
    value = match.group(2)
    if value is None:
        return key, "true"
    value = value.strip()
    if value.startswith('"') and value.endswith('"'):
```

```
        value = value[1:-1]
    elif value.startswith('"'):
        value = value[1:]
    return key, value


def parse_genbank_features(path: Path) -> list[Feature]:
    features: list[Feature] = []
    current: Feature | None = None
    current_qualifier: str | None = None
    in_features = False

    for raw_line in path.read_text(encoding="utf-8",
errors="replace").splitlines():
        if raw_line.startswith("FEATURES"):
            in_features = True
            continue
        if raw_line.startswith("ORIGIN"):
            break
        if not in_features:
            continue

        feature_match = re.match(r"^     (\S+)\s+(.+)$", raw_line)
        if feature_match and not
raw_line[21:].lstrip().startswith("/"):
            current = Feature(
                key=feature_match.group(1),
                location=feature_match.group(2).strip(),
                qualifiers={},
            )
            features.append(current)
            current_qualifier = None
            continue

        if current is None:
            continue

        qualifier_match = parse_qualifier(raw_line)
        if qualifier_match:
            key, value = qualifier_match
            current.qualifiers.setdefault(key, []).append(value)
            current_qualifier = key
            if value.endswith('"'):
                current.qualifiers[key][-1] = value[:-1]
            continue

        if current_qualifier and raw_line.startswith("
"):
            continuation = raw_line.strip()
            if continuation.endswith('"'):
```

```
                continuation = continuation[:-1]
            if continuation:
                current.qualifiers[current_qualifier][-1] += " " +
continuation

    return features


def qualifier_text(feature: Feature, names: tuple[str, ...]) -> str:
    values: list[str] = []
    for name in names:
        values.extend(feature.qualifiers.get(name, []))
    return "; ".join(value.strip() for value in values if
value.strip())


def clean_text(text: str) -> str:
    return re.sub(r"\s+", " ", text).strip()


def find_hits(sample: str, path: Path, targets: list[Target]) ->
list[Hit]:
    hits: list[Hit] = []
    features = parse_genbank_features(path)
    compiled = [
        (target, [re.compile(pattern, re.IGNORECASE) for pattern in
target.patterns])
        for target in targets
    ]

    for feature in features:
        if feature.key != "CDS":
            continue
        gene = qualifier_text(feature, ("gene", "locus_tag"))
        product = qualifier_text(feature, ("product",))
        note = qualifier_text(feature, ("note", "function"))
        searchable = clean_text(" | ".join([gene, product, note]))
        if not searchable:
            continue

        for target, patterns in compiled:
            if any(pattern.search(searchable) for pattern in
patterns):
                hits.append(
                    Hit(
                        sample=sample,
                        target=target.label,
                        gene_class=target.gene_class,
                        location=feature.location,
                        gene=gene or "-",
```

```
                        product=product or "-",
                        note=note or "-",
                        matched_text=searchable,
                        source_file=path,
                    )
                )

    return hits


def write_matrix(path: Path, samples: list[tuple[str, Path]], targets:
list[Target], hits: list[Hit]) -> None:
    present = {(hit.sample, hit.target) for hit in hits}
    with path.open("w", newline="") as handle:
        writer = csv.writer(handle, delimiter="\t")
        writer.writerow(["sample"] + [target.label for target in
targets])
        for sample, _ in samples:
            writer.writerow(
                [sample] + [1 if (sample, target.label) in present
else 0 for target in targets]
            )


def write_hits(path: Path, hits: list[Hit]) -> None:
    with path.open("w", newline="") as handle:
        writer = csv.writer(handle, delimiter="\t")
        writer.writerow(
            [
                "sample",
                "amr_gene",
                "class",
                "location",
                "gene_or_locus_tag",
                "product",
                "note",
                "matched_text",
                "source_file",
            ]
        )
        for hit in hits:
            writer.writerow(
                [
                    hit.sample,
                    hit.target,
                    hit.gene_class,
                    hit.location,
                    hit.gene,
                    hit.product,
                    hit.note,
```

```
                    hit.matched_text,
                    str(hit.source_file),
                ]
            )


def rotate_label(svg: list[str], x: float, y: float, label: str) ->
None:
    svg.append(f'<g transform="translate({x:.1f},{y:.1f})
rotate(-55)">')
    svg.append(f'<text class="label" text-
anchor="start">{html.escape(label)}</text>')
    svg.append("</g>")


def write_svg(
    path: Path,
    samples: list[tuple[str, Path]],
    targets: list[Target],
    hits: list[Hit],
    include_psh111: bool,
) -> None:
    present = {(hit.sample, hit.target) for hit in hits}
    left = 170
    top = 150
    cell = 35
    row_h = 30
    label_h = 112
    width = left + len(targets) * cell + 120
    height = top + len(samples) * row_h + 125

    title = "Corrected AMR Genes: pNDM and NCTR Contig 3 Plasmids"
    if include_psh111:
        title = "Corrected AMR Genes: pSH111_227, pNDM, and NCTR
Contig 3 Plasmids"

    svg: list[str] = []
    svg.append(
        f'<svg xmlns="http://www.w3.org/2000/svg" width="{width}"
height="{height}" '
        f'viewBox="0 0 {width} {height}">'
    )
    svg.append('<rect width="100%" height="100%" fill="#ffffff"/>')
    svg.append(
        "<style>"
        "text{font-family:Arial,Helvetica,sans-serif}"
        ".title{font-size:18px;font-weight:700}"
        ".small{font-size:11px}"
        ".label{font-size:12px}"
        ".sample{font-size:12px}"
```

```
        ".ref{font-weight:700}"
        "</style>"
    )
    svg.append(f'<text x="24" y="32"
class="title">{html.escape(title)}</text>')
    svg.append(
        '<text x="24" y="54" class="small" fill="#444">'
        "Filled cells indicate AMR-associated CDS annotations detected
from gene/product/note fields."
        "</text>"
    )

    for i, target in enumerate(targets):
        x = left + i * cell + cell / 2
        rotate_label(svg, x, top - 12, target.label)
        svg.append(
            f'<rect x="{left + i * cell}" y="{top - label_h}"
width="{cell}" height="8" '
            f'fill="{COLORS[target.gene_class]}"/>'
        )

    for row_index, (sample, _) in enumerate(samples):
        y = top + row_index * row_h
        label_class = "sample ref" if sample in {"pSH111_227", "pNDM"}
else "sample"
        svg.append(
            f'<text x="{left - 10}" y="{y + 20}" class="{label_class}"
'
            f'text-anchor="end">{html.escape(sample)}</text>'
        )
        for col_index, target in enumerate(targets):
            x = left + col_index * cell
            has_hit = (sample, target.label) in present
            fill = COLORS[target.gene_class] if has_hit else "#eeeeee"
            stroke = "#777777" if has_hit else "#cfcfcf"
            svg.append(
                f'<rect x="{x}" y="{y}" width="{cell - 2}"
height="{row_h - 3}" rx="2" '
                f'fill="{fill}" stroke="{stroke}" stroke-width="0.7"/
>'
            )
            if has_hit:
                svg.append(
                    f'<text x="{x + (cell - 2) / 2}" y="{y + 19}"
class="small" '
                    f'text-anchor="middle" fill="#ffffff">+</text>'
                )

    legend_y = top + len(samples) * row_h + 42
    legend_x = 24
```

```
    seen_classes: list[str] = []
    for target in targets:
        if target.gene_class not in seen_classes:
            seen_classes.append(target.gene_class)

    for gene_class in seen_classes:
        svg.append(
            f'<rect x="{legend_x}" y="{legend_y - 14}" width="14"
height="14" '
            f'fill="{COLORS[gene_class]}"/>'
        )
        svg.append(
            f'<text x="{legend_x + 20}" y="{legend_y - 3}"
class="small">'
            f'{html.escape(gene_class)}</text>'
        )
        legend_x += max(110, len(gene_class) * 8 + 45)

    svg.append(
        f'<rect x="24" y="{legend_y + 22}" width="14" height="14" '
        'fill="#eeeeee" stroke="#cfcfcf"/>'
    )
    svg.append(f'<text x="44" y="{legend_y + 33}" class="small">not
detected</text>')
    svg.append("</svg>")
    path.write_text("\n".join(svg))


def main() -> int:
    args = parse_args()
    try:
        samples = sample_paths(args.project_dir, args.pndm,
args.include_psh111)
    except FileNotFoundError as error:
        print(f"ERROR: {error}", file=sys.stderr)
        return 2

    all_hits: list[Hit] = []
    for sample, path in samples:
        all_hits.extend(find_hits(sample, path, TARGETS))

    if args.show_empty_columns:
        targets = TARGETS
    else:
        detected = {hit.target for hit in all_hits}
        targets = [target for target in TARGETS if target.label in
detected]

    args.outdir.mkdir(parents=True, exist_ok=True)
    matrix_path = args.outdir / f"{args.prefix}_matrix.tsv"
```

```python
    hits_path = args.outdir / f"{args.prefix}_hits.tsv"
    svg_path = args.outdir / f"{args.prefix}_heatmap.svg"

    write_matrix(matrix_path, samples, targets, all_hits)
    write_hits(hits_path, all_hits)
    write_svg(svg_path, samples, targets, all_hits,
args.include_psh111)

    print("Samples used:")
    for sample, path in samples:
        print(f"  {sample}: {path}")
    print()
    print("Detected AMR targets:")
    for target in targets:
        samples_with_hit = sorted({hit.sample for hit in all_hits if
hit.target == target.label})
        print(f"  {target.label}: {', '.join(samples_with_hit)}")
    print()
    print("Saved:")
    print(f"  {matrix_path}")
    print(f"  {hits_path}")
    print(f"  {svg_path}")
    return 0


if __name__ == "__main__":
    raise SystemExit(main())
```


Script 20: extract_card_contig3_amr_table.py
Source file: extract_card_contig3_amr_table.py
Line count: 503


```python
#!/usr/bin/env python3
"""
Extract CARD AMR hits from contig/sequence 3 in the Salmonella
annotation workbook
and build a presence/absence TSV plus a colored HTML table.

This script intentionally uses only the Python standard library so it
can run on
macOS without installing openpyxl.
"""

from __future__ import annotations
```

```python
import argparse
import csv
import html
import re
import sys
import xml.etree.ElementTree as ET
from collections import defaultdict
from pathlib import Path
from zipfile import ZipFile


DEFAULT_XLSX = Path(
    "<project_data_directory>/"
    "Salm sequencing paper 2025/Sequencing data/Annotated data/"
    "HMR genes annotation_Salmonella.xlsx"
)
DEFAULT_OUTDIR = Path("<project_directory>/card_contig3_amr_results")

NS_MAIN = {"m": "http://schemas.openxmlformats.org/spreadsheetml/2006/
main"}
NS_RELS = {"r": "http://schemas.openxmlformats.org/officeDocument/
2006/relationships"}
NS_PKG = {"pr": "http://schemas.openxmlformats.org/package/2006/
relationships"}


CLASS_COLORS = {
    "beta-lactam": "#c0392b",
    "aminoglycoside": "#2f6f9f",
    "tetracycline": "#d95f02",
    "sulfonamide": "#1b9e77",
    "phenicol": "#7b3294",
    "chloramphenicol": "#7b3294",
    "trimethoprim": "#66a61e",
    "fluoroquinolone": "#1f3a93",
    "quinolone": "#1f3a93",
    "macrolide": "#e7298a",
    "rifamycin": "#8c510a",
    "fosfomycin": "#a6761d",
    "glycopeptide": "#7570b3",
    "lincosamide": "#e6ab02",
    "streptogramin": "#e6ab02",
    "multidrug": "#4d4d4d",
    "efflux": "#4d4d4d",
    "biocide": "#666666",
    "triclosan": "#666666",
    "unknown": "#6c757d",
}
```

```
def column_index(cell_ref: str) -> int:
    letters = "".join(ch for ch in cell_ref if ch.isalpha())
    value = 0
    for ch in letters:
        value = value * 26 + (ord(ch.upper()) - ord("A") + 1)
    return value - 1


def text_from_element(element: ET.Element) -> str:
    return "".join(element.itertext()) if element is not None else ""


def read_shared_strings(zf: ZipFile) -> list[str]:
    try:
        raw = zf.read("xl/sharedStrings.xml")
    except KeyError:
        return []
    root = ET.fromstring(raw)
    strings = []
    for item in root.findall("m:si", NS_MAIN):
        strings.append(text_from_element(item))
    return strings


def sheet_path_for_name(zf: ZipFile, sheet_name: str) -> str:
    workbook = ET.fromstring(zf.read("xl/workbook.xml"))
    rels = ET.fromstring(zf.read("xl/_rels/workbook.xml.rels"))

    rel_targets = {}
    for rel in rels.findall("pr:Relationship", NS_PKG):
        rel_targets[rel.attrib["Id"]] = rel.attrib["Target"]

    for sheet in workbook.findall("m:sheets/m:sheet", NS_MAIN):
        if sheet.attrib.get("name") != sheet_name:
            continue
        rel_id = sheet.attrib[f"{{{NS_RELS['r']}}}id"]
        target = rel_targets[rel_id]
        if target.startswith("/"):
            target = target.lstrip("/")
        elif not target.startswith("xl/"):
            target = f"xl/{target}"
        return target

    available = [s.attrib.get("name", "") for s in 
workbook.findall("m:sheets/m:sheet", NS_MAIN)]
    raise SystemExit(f"Could not find sheet {sheet_name!r}. Available 
sheets: {', '.join(available)}")


def read_xlsx_sheet(xlsx: Path, sheet_name: str) -> list[dict[str,
```

```
str]]:
    with ZipFile(xlsx) as zf:
        shared_strings = read_shared_strings(zf)
        sheet_path = sheet_path_for_name(zf, sheet_name)
        root = ET.fromstring(zf.read(sheet_path))

        rows = []
        for row in root.findall(".//m:sheetData/m:row", NS_MAIN):
            values_by_col = {}
            for cell in row.findall("m:c", NS_MAIN):
                ref = cell.attrib.get("r", "")
                if not ref:
                    continue
                col = column_index(ref)
                cell_type = cell.attrib.get("t", "")

                if cell_type == "inlineStr":
                    value = text_from_element(cell.find("m:is",
NS_MAIN))
                else:
                    value_element = cell.find("m:v", NS_MAIN)
                    value = value_element.text if value_element is not
None else ""
                    if cell_type == "s" and value != "":
                        value = shared_strings[int(value)]

                values_by_col[col] = value.strip() if
isinstance(value, str) else str(value)

            if values_by_col:
                max_col = max(values_by_col)
                rows.append([values_by_col.get(i, "") for i in
range(max_col + 1)])

    if not rows:
        return []

    headers = [h.strip() for h in rows[0]]
    records = []
    for values in rows[1:]:
        if not any(values):
            continue
        row = {}
        for idx, header in enumerate(headers):
            if header:
                row[header] = values[idx].strip() if idx < len(values)
else ""
        records.append(row)
    return records
```

```
def natural_key(text: str) -> tuple:
    return tuple(int(part) if part.isdigit() else part.lower() for
part in re.split(r"(\d+)", text))


def is_sequence3(value: str) -> bool:
    normalized = re.sub(r"[\s_-]+", "", str(value).strip().lower())
    return normalized in {"3", "03", "sequence3", "seq3", "contig3"}


def number_value(value: str) -> float | None:
    value = str(value).strip().replace("%", "")
    if not value:
        return None
    try:
        return float(value)
    except ValueError:
        return None


def split_resistance_terms(text: str) -> list[str]:
    terms = []
    for part in re.split(r";|,", text or ""):
        part = re.sub(r"\s+", " ", part.strip())
        if part:
            terms.append(part)
    return terms


def class_for_hit(row: dict[str, str]) -> str:
    haystack = " ".join(
        [
            row.get("RESISTANCE", ""),
            row.get("PRODUCT", ""),
            row.get("GENE", ""),
        ]
    ).lower()

    if any(word in haystack for word in ["beta-lactam",
"cephalosporin", "cephamycin", "carbapenem", "penam"]):
        return "beta-lactam"
    if "aminoglycoside" in haystack:
        return "aminoglycoside"
    if "tetracycline" in haystack or "glycylcycline" in haystack:
        return "tetracycline"
    if "sulfonamide" in haystack:
        return "sulfonamide"
    if "trimethoprim" in haystack or "diaminopyrimidine" in haystack:
        return "trimethoprim"
```

```
    if "phenicol" in haystack or "chloramphenicol" in haystack:
        return "phenicol"
    if "fluoroquinolone" in haystack:
        return "fluoroquinolone"
    if "quinolone" in haystack:
        return "quinolone"
    if "macrolide" in haystack:
        return "macrolide"
    if "rifamycin" in haystack:
        return "rifamycin"
    if "fosfomycin" in haystack:
        return "fosfomycin"
    if "glycopeptide" in haystack:
        return "glycopeptide"
    if "lincosamide" in haystack:
        return "lincosamide"
    if "streptogramin" in haystack:
        return "streptogramin"
    if "efflux" in haystack:
        return "efflux"
    if "multidrug" in haystack or "multiple antibiotic" in haystack:
        return "multidrug"
    if "biocide" in haystack or "triclosan" in haystack or "quaternary
ammonium" in haystack or "qac" in haystack:
        return "biocide"
    return "unknown"


def clean_gene_name(gene: str) -> str:
    gene = re.sub(r"\s+", " ", str(gene).strip())
    return gene or "unnamed_CARD_hit"


def filter_card_rows(
    rows: list[dict[str, str]],
    sequence: str,
    nctr_only: bool,
    min_identity: float,
    min_coverage: float,
) -> list[dict[str, str]]:
    hits = []
    for row in rows:
        sample = row.get("#FILE", row.get("FILE", "")).strip()
        seq = row.get("SEQUENCE", "").strip()
        gene = clean_gene_name(row.get("GENE", ""))

        if sequence == "3" and not is_sequence3(seq):
            continue
        if sequence != "3" and seq != sequence:
            continue
```

```
        if nctr_only and not sample.startswith("NCTR"):
            continue

        identity = number_value(row.get("%IDENTITY", ""))
        coverage = number_value(row.get("%COVERAGE", ""))
        if identity is not None and identity < min_identity:
            continue
        if coverage is not None and coverage < min_coverage:
            continue

        clean = dict(row)
        clean["Sample"] = sample
        clean["Sequence"] = seq
        clean["Gene_clean"] = gene
        clean["Drug_class"] = class_for_hit(clean)
        clean["Resistance_terms_clean"] = ";
".join(split_resistance_terms(row.get("RESISTANCE", "")))
        hits.append(clean)
    return hits


def workbook_samples(rows: list[dict[str, str]], nctr_only: bool) ->
list[str]:
    samples = set()
    for row in rows:
        sample = row.get("#FILE", row.get("FILE", "")).strip()
        if not sample:
            continue
        if nctr_only and not sample.startswith("NCTR"):
            continue
        samples.add(sample)
    return sorted(samples, key=natural_key)


def write_hits_tsv(hits: list[dict[str, str]], path: Path) -> None:
    columns = [
        "Sample",
        "Sequence",
        "START",
        "END",
        "STRAND",
        "Gene_clean",
        "Drug_class",
        "Resistance_terms_clean",
        "%COVERAGE",
        "%IDENTITY",
        "ACCESSION",
        "PRODUCT",
    ]
    with path.open("w", newline="") as handle:
```

```
        writer = csv.DictWriter(handle, fieldnames=columns,
delimiter="\t", extrasaction="ignore")
        writer.writeheader()
        writer.writerows(hits)


def write_presence_absence_tsv(
    samples: list[str],
    genes: list[str],
    present: dict[str, set[str]],
    path: Path,
) -> None:
    with path.open("w", newline="") as handle:
        writer = csv.writer(handle, delimiter="\t")
        writer.writerow(["Sample", *genes])
        for sample in samples:
            writer.writerow([sample, *[1 if gene in present[sample]
else 0 for gene in genes]])


def write_colored_html(
    samples: list[str],
    genes: list[str],
    present: dict[str, set[str]],
    gene_class: dict[str, str],
    gene_resistance: dict[str, set[str]],
    hits: list[dict[str, str]],
    path: Path,
    title: str,
) -> None:
    classes_used = []
    for gene in genes:
        cls = gene_class.get(gene, "unknown")
        if cls not in classes_used:
            classes_used.append(cls)

    cell_count = sum(1 for sample in samples for gene in genes if gene
in present[sample])
    subtitle = (
        f"CARD sheet filtered to sequence/contig 3. "
        f"{len(samples)} isolates, {len(genes)} CARD genes,
{len(hits)} hits, {cell_count} present cells."
    )

    parts = [
        "<!doctype html>",
        "<html>",
        "<head>",
        "<meta charset='utf-8'>",
        f"<title>{html.escape(title)}</title>",
```

```
        "<style>",
        "body{font-family:Arial,Helvetica,sans-
serif;margin:24px;color:#222;background:#fff}",
        "h1{font-size:20px;margin:0 0 6px 0}",
        ".subtitle{font-size:13px;color:#555;margin-bottom:16px}",
        ".wrap{display:flex;gap:18px;align-items:flex-start}",
        ".tablebox{overflow:auto;max-width:calc(100vw -
260px);border:1px solid #d7d7d7}",
        "table{border-collapse:collapse;font-
size:12px;background:#fff}",
        "th,td{border:1px solid #d7d7d7;padding:5px 7px;text-
align:center;white-space:nowrap}",
        "th{background:#f4f4f4;font-
weight:700;position:sticky;top:0;z-index:2}",

"th.sample,td.sample{position:sticky;left:0;background:#fff;text-
align:left;font-weight:700;z-index:3}",
        "th.sample{background:#f4f4f4;z-index:4}",
        "th.gene{writing-mode:vertical-
rl;transform:rotate(180deg);height:145px;vertical-align:bottom;min-
width:24px}",
        "td.present{color:#fff;font-weight:700}",
        "td.absent{background:#f7f7f7;color:#bbb}",
        ".legend{min-width:220px;border:1px solid
#d7d7d7;padding:12px;background:#fff}",
        ".legend h2{font-size:14px;margin:0 0 10px 0}",
        ".legend-row{display:flex;align-items:center;gap:8px;font-
size:12px;margin:7px 0}",
        ".swatch{width:16px;height:16px;border-radius:3px;border:1px
solid rgba(0,0,0,.18)}",
        ".notes{font-size:12px;color:#555;margin-top:14px;max-
width:980px;line-height:1.4}",
        "</style>",
        "</head>",
        "<body>",
        f"<h1>{html.escape(title)}</h1>",
        f"<div class='subtitle'>{html.escape(subtitle)}</div>",
    ]

    if not genes:
        parts.extend(
            [
                "<p>No CARD AMR hits were found in sequence/contig 3
with the selected filters.</p>",
                "</body></html>",
            ]
        )
        path.write_text("\n".join(parts))
        return
```

```
    parts.append("<div class='wrap'>")
    parts.append("<div class='tablebox'>")
    parts.append("<table>")
    parts.append("<thead><tr><th class='sample'>Isolate</th>")
    for gene in genes:
        cls = gene_class.get(gene, "unknown")
        color = CLASS_COLORS.get(cls, CLASS_COLORS["unknown"])
        terms = "; ".join(sorted(gene_resistance.get(gene, [])))
        title_text = f"{gene} | {cls}"
        if terms:
            title_text += f" | {terms}"
        parts.append(
            f"<th class='gene' title='{html.escape(title_text)}' "
            f"style='border-bottom:4px solid
{color}'>{html.escape(gene)}</th>"
        )
    parts.append("</tr></thead><tbody>")

    for sample in samples:
        parts.append(f"<tr><td class='sample'>{html.escape(sample)}</
td>")
        for gene in genes:
            cls = gene_class.get(gene, "unknown")
            color = CLASS_COLORS.get(cls, CLASS_COLORS["unknown"])
            if gene in present[sample]:
                parts.append(f"<td class='present' style='background:
{color}'>+</td>")
            else:
                parts.append("<td class='absent'>-</td>")
        parts.append("</tr>")

    parts.append("</tbody></table></div>")
    parts.append("<div class='legend'><h2>Antimicrobial class</h2>")
    for cls in classes_used:
        color = CLASS_COLORS.get(cls, CLASS_COLORS["unknown"])
        parts.append(
            f"<div class='legend-row'><span class='swatch'
style='background:{color}'></span>"
            f"<span>{html.escape(cls)}</span></div>"
        )
    parts.append("</div></div>")
    parts.append(
        "<div class='notes'>Each colored cell means the CARD sheet
contains that gene for that isolate "
        "on sequence/contig 3. CARD may report acquired genes, efflux
pumps, and regulatory genes; "
        "review the hits TSV for identity, coverage, product, and
resistance terms.</div>"
    )
    parts.append("</body></html>")
```

```
    path.write_text("\n".join(parts))


def main() -> int:
    parser = argparse.ArgumentParser(
        description="Extract CARD AMR genes from sequence/contig 3 and
generate a colored table."
    )
    parser.add_argument("--xlsx", type=Path, default=DEFAULT_XLSX,
help="Input Excel workbook.")
    parser.add_argument("--sheet", default="CARD", help="Workbook
sheet to read.")
    parser.add_argument("--sequence", default="3", help="Sequence/
contig value to extract. Default: 3.")
    parser.add_argument("--outdir", type=Path, default=DEFAULT_OUTDIR,
help="Output directory.")
    parser.add_argument("--include-non-nctr", action="store_true",
help="Also include non-NCTR samples.")
    parser.add_argument(
        "--only-samples-with-hits",
        action="store_true",
        help="Only show isolates with at least one CARD hit on the
selected sequence/contig.",
    )
    parser.add_argument("--min-identity", type=float, default=0.0,
help="Minimum CARD percent identity.")
    parser.add_argument("--min-coverage", type=float, default=0.0,
help="Minimum CARD percent coverage.")
    parser.add_argument(
        "--title",
        default="CARD AMR genes detected in sequence/contig 3 of
Salmonella Heidelberg isolates",
        help="Title for the HTML table.",
    )
    args = parser.parse_args()

    if not args.xlsx.exists():
        raise SystemExit(f"Cannot find workbook: {args.xlsx}")

    args.outdir.mkdir(parents=True, exist_ok=True)
    rows = read_xlsx_sheet(args.xlsx, args.sheet)
    hits = filter_card_rows(
        rows,
        sequence=args.sequence,
        nctr_only=not args.include_non_nctr,
        min_identity=args.min_identity,
        min_coverage=args.min_coverage,
    )

    nctr_only = not args.include_non_nctr
```

```
    if args.only_samples_with_hits:
        samples = sorted({hit["Sample"] for hit in hits},
key=natural_key)
    else:
        samples = workbook_samples(rows, nctr_only=nctr_only)
    present: dict[str, set[str]] = defaultdict(set)
    gene_class: dict[str, str] = {}
    gene_resistance: dict[str, set[str]] = defaultdict(set)

    for hit in hits:
        sample = hit["Sample"]
        gene = hit["Gene_clean"]
        cls = hit["Drug_class"]
        present[sample].add(gene)
        gene_class.setdefault(gene, cls)
        for term in
split_resistance_terms(hit.get("Resistance_terms_clean", "")):
            gene_resistance[gene].add(term)

    genes = sorted(
        {hit["Gene_clean"] for hit in hits},
        key=lambda gene: (gene_class.get(gene, "unknown"),
natural_key(gene)),
    )

    hits_tsv = args.outdir / "card_contig3_amr_hits.tsv"
    matrix_tsv = args.outdir / "card_contig3_amr_presence_absence.tsv"
    html_path = args.outdir / "card_contig3_amr_colored_table.html"

    write_hits_tsv(hits, hits_tsv)
    write_presence_absence_tsv(samples, genes, present, matrix_tsv)
    write_colored_html(samples, genes, present, gene_class,
gene_resistance, hits, html_path, args.title)

    print(f"CARD sheet rows scanned: {len(rows)}")
    print(f"Sequence/contig {args.sequence} CARD hits kept:
{len(hits)}")
    print(f"Samples included: {len(samples)}")
    print(f"CARD genes detected: {len(genes)}")
    print("Saved:")
    print(f"  {hits_tsv}")
    print(f"  {matrix_tsv}")
    print(f"  {html_path}")
    return 0


if __name__ == "__main__":
    sys.exit(main())
```


Script 21: extract_card_amrfinder_contig3_combined_table.py
Source file: extract_card_amrfinder_contig3_combined_table.py
Line count: 623


```python
#!/usr/bin/env python3
"""
Extract contig3/sequence3 AMR genes from the CARD and AMRfinderPlus
sheets
in the Salmonella annotation workbook and consolidate them into one
colored table.

No external Python packages are required.
"""

from __future__ import annotations

import argparse
import csv
import html
import re
import sys
import xml.etree.ElementTree as ET
from collections import defaultdict
from pathlib import Path
from zipfile import ZipFile


DEFAULT_XLSX = Path(
    "<project_data_directory>/"
    "Salm sequencing paper 2025/Sequencing data/Annotated data/"
    "HMR genes annotation_Salmonella.xlsx"
)
DEFAULT_OUTDIR = Path("<project_directory>/
card_amrfinder_contig3_combined_results")

NS_MAIN = {"m": "http://schemas.openxmlformats.org/spreadsheetml/2006/
main"}
NS_RELS = {"r": "http://schemas.openxmlformats.org/officeDocument/
2006/relationships"}
NS_PKG = {"pr": "http://schemas.openxmlformats.org/package/2006/
relationships"}

STATUS_COLORS = {
    "Both": "#1b9e77",
    "CARD": "#2f6f9f",
    "AMRFinder": "#d95f02",
```

```
    "Absent": "#eeeeee",
}

CLASS_COLORS = {
    "beta-lactam": "#c0392b",
    "aminoglycoside": "#2f6f9f",
    "macrolide": "#e7298a",
    "phenicol": "#7b3294",
    "sulfonamide": "#1b9e77",
    "tetracycline": "#d95f02",
    "trimethoprim": "#66a61e",
    "biocide": "#666666",
    "mercury/stress": "#7570b3",
    "unknown": "#6c757d",
}

CLASS_ORDER = {
    "beta-lactam": 0,
    "aminoglycoside": 1,
    "macrolide": 2,
    "phenicol": 3,
    "sulfonamide": 4,
    "tetracycline": 5,
    "trimethoprim": 6,
    "biocide": 7,
    "mercury/stress": 8,
    "unknown": 99,
}


def column_index(cell_ref: str) -> int:
    letters = "".join(ch for ch in cell_ref if ch.isalpha())
    value = 0
    for ch in letters:
        value = value * 26 + (ord(ch.upper()) - ord("A") + 1)
    return value - 1


def element_text(element: ET.Element | None) -> str:
    return "".join(element.itertext()) if element is not None else ""


def read_shared_strings(zf: ZipFile) -> list[str]:
    try:
        root = ET.fromstring(zf.read("xl/sharedStrings.xml"))
    except KeyError:
        return []
    return [element_text(item) for item in root.findall("m:si",
NS_MAIN)]
```

```
def sheet_path_for_name(zf: ZipFile, sheet_name: str) -> str:
    workbook = ET.fromstring(zf.read("xl/workbook.xml"))
    rels = ET.fromstring(zf.read("xl/_rels/workbook.xml.rels"))

    rel_targets = {
        rel.attrib["Id"]: rel.attrib["Target"]
        for rel in rels.findall("pr:Relationship", NS_PKG)
    }

    for sheet in workbook.findall("m:sheets/m:sheet", NS_MAIN):
        if sheet.attrib.get("name") != sheet_name:
            continue
        rel_id = sheet.attrib[f"{{{NS_RELS['r']}}}id"]
        target = rel_targets[rel_id]
        if target.startswith("/"):
            target = target.lstrip("/")
        elif not target.startswith("xl/"):
            target = f"xl/{target}"
        return target

    available = [s.attrib.get("name", "") for s in
workbook.findall("m:sheets/m:sheet", NS_MAIN)]
    raise SystemExit(f"Could not find sheet {sheet_name!r}. Available
sheets: {', '.join(available)}")


def read_xlsx_sheet(xlsx: Path, sheet_name: str) -> list[dict[str,
str]]:
    with ZipFile(xlsx) as zf:
        shared_strings = read_shared_strings(zf)
        sheet_path = sheet_path_for_name(zf, sheet_name)
        root = ET.fromstring(zf.read(sheet_path))

        rows = []
        for row in root.findall(".//m:sheetData/m:row", NS_MAIN):
            values_by_col = {}
            for cell in row.findall("m:c", NS_MAIN):
                ref = cell.attrib.get("r", "")
                if not ref:
                    continue
                col = column_index(ref)
                cell_type = cell.attrib.get("t", "")

                if cell_type == "inlineStr":
                    value = element_text(cell.find("m:is", NS_MAIN))
                else:
                    value_element = cell.find("m:v", NS_MAIN)
                    value = value_element.text if value_element is not
None else ""
```

```
                    if cell_type == "s" and value != "":
                        value = shared_strings[int(value)]

                values_by_col[col] = value.strip() if
isinstance(value, str) else str(value)

            if values_by_col:
                max_col = max(values_by_col)
                rows.append([values_by_col.get(i, "") for i in
range(max_col + 1)])

    if not rows:
        return []

    headers = [h.strip() for h in rows[0]]
    records = []
    for values in rows[1:]:
        if not any(values):
            continue
        record = {}
        for i, header in enumerate(headers):
            if header:
                record[header] = values[i].strip() if i < len(values)
else ""
        records.append(record)
    return records


def natural_key(text: str) -> tuple:
    return tuple(int(part) if part.isdigit() else part.lower() for
part in re.split(r"(\d+)", text))


def is_sequence3(value: str) -> bool:
    normalized = re.sub(r"[\s_-]+", "", str(value).strip().lower())
    return normalized in {"3", "03", "sequence3", "seq3", "contig3"}


def number_value(value: str) -> float | None:
    value = str(value).strip().replace("%", "")
    if not value:
        return None
    try:
        return float(value)
    except ValueError:
        return None


def compact_gene_key(gene: str) -> str:
    gene = gene.strip().replace("’", "'").replace("`", "'")
```

```
    gene = re.sub(r"\s+", "", gene)
    return gene.lower()


def canonical_gene(gene: str, exact_gene_names: bool = False) -> str:
    raw = re.sub(r"\s+", " ", gene.strip())
    key = compact_gene_key(raw)

    if exact_gene_names:
        return raw

    # Merge common CARD/AMRFinder naming differences into one column.
    mapping = {
        "tem-1": "blaTEM-1",
        "blatem-1": "blaTEM-1",
        "mpha": "mph(A)",
        "mph(a)": "mph(A)",
        "tetm": "tet(M)",
        "tet(m)": "tet(M)",
        "teta": "tet(A)",
        "tet(a)": "tet(A)",
        "aada1": "aadA1/ANT(3'')",
        "ant(3'')-ia": "aadA1/ANT(3'')",
        "ant(3'')-iia": "aadA1/ANT(3'')",
        "aada2": "aadA2",
        "aac(3)-via": "aac(3)-VIa",
        "ant(2'')-ia": "ant(2'')-Ia",
        "aph(3'')-ib": "aph(3'')-Ib",
        "aph(3')-ia": "aph(3')-Ia",
        "aph(6)-id": "aph(6)-Id",
        "cmly1": "cmlA1",
        "cmla1": "cmlA1",
        "cmla5": "cmlA5",
        "dfra1": "dfrA1",
        "dfra12": "dfrA12",
        "flor": "floR",
        "sul1": "sul1",
        "sul2": "sul2",
        "sul3": "sul3",
        "qach": "qacH",
        "qacedelta1": "qacEdelta1",
        "qacl": "qacL",
    }
    if key in mapping:
        return mapping[key]

    if re.fullmatch(r"(bla)?cmy-\d+[a-z]?", key):
        # CARD may call this CMY-59 while AMRFinder calls it blaCMY-2.
        # Keep a family-level column and preserve raw calls in the
mapping TSV.
```

```python
        return "blaCMY"

    if key.startswith("mer"):
        return key[:3] + key[3:].upper()

    return raw


def card_class(row: dict[str, str]) -> str:
    haystack = " ".join([row.get("RESISTANCE", ""), row.get("PRODUCT",
""), row.get("GENE", "")]).lower()
    if any(word in haystack for word in ["beta-lactam",
"cephalosporin", "cephamycin", "carbapenem", "penam"]):
        return "beta-lactam"
    if "aminoglycoside" in haystack:
        return "aminoglycoside"
    if "macrolide" in haystack:
        return "macrolide"
    if "phenicol" in haystack or "chloramphenicol" in haystack:
        return "phenicol"
    if "sulfonamide" in haystack:
        return "sulfonamide"
    if "tetracycline" in haystack:
        return "tetracycline"
    if "trimethoprim" in haystack or "diaminopyrimidine" in haystack:
        return "trimethoprim"
    if "qac" in haystack or "quaternary ammonium" in haystack or
"biocide" in haystack:
        return "biocide"
    if "mercur" in haystack:
        return "mercury/stress"
    return "unknown"


def amrfinder_class(row: dict[str, str]) -> str:
    class_value = row.get("Class", "").strip().lower()
    if class_value == "beta-lactam":
        return "beta-lactam"
    if class_value == "aminoglycoside":
        return "aminoglycoside"
    if class_value == "macrolide":
        return "macrolide"
    if class_value == "phenicol":
        return "phenicol"
    if class_value == "sulfonamide":
        return "sulfonamide"
    if class_value == "tetracycline":
        return "tetracycline"
    if class_value == "trimethoprim":
        return "trimethoprim"
```

```
    if class_value == "quaternary ammonium":
        return "biocide"
    if class_value == "mercury":
        return "mercury/stress"
    return class_value or "unknown"


def passes_threshold(value: str, minimum: float) -> bool:
    parsed = number_value(value)
    return parsed is None or parsed >= minimum


def extract_card_hits(rows: list[dict[str, str]], args) ->
list[dict[str, str]]:
    hits = []
    for row in rows:
        sample = row.get("#FILE", "").strip()
        if args.nctr_only and not sample.startswith("NCTR"):
            continue
        if not is_sequence3(row.get("SEQUENCE", "")):
            continue
        if not passes_threshold(row.get("%IDENTITY", ""),
args.min_card_identity):
            continue
        if not passes_threshold(row.get("%COVERAGE", ""),
args.min_card_coverage):
            continue

        raw_gene = row.get("GENE", "").strip()
        if not raw_gene:
            continue
        gene = canonical_gene(raw_gene,
exact_gene_names=args.exact_gene_names)
        hits.append(
            {
                "sample": sample,
                "sequence": row.get("SEQUENCE", ""),
                "tool": "CARD",
                "raw_gene": raw_gene,
                "gene": gene,
                "class": card_class(row),
                "subclass_or_resistance": row.get("RESISTANCE", ""),
                "start": row.get("START", ""),
                "end": row.get("END", ""),
                "strand": row.get("STRAND", ""),
                "percent_coverage": row.get("%COVERAGE", ""),
                "percent_identity": row.get("%IDENTITY", ""),
                "accession": row.get("ACCESSION", ""),
                "product_or_sequence_name": row.get("PRODUCT", ""),
                "element_type": "",
```

```
            }
        )
    return hits


def extract_amrfinder_hits(rows: list[dict[str, str]], args) ->
list[dict[str, str]]:
    allowed_types = {"AMR"}
    if args.include_amrfinder_stress:
        allowed_types.add("STRESS")

    hits = []
    for row in rows:
        sample = row.get("Name", "").strip()
        if args.nctr_only and not sample.startswith("NCTR"):
            continue
        if not is_sequence3(row.get("Contig id", "")):
            continue
        if row.get("Element type", "").strip() not in allowed_types:
            continue
        if not passes_threshold(row.get("% Identity to reference
sequence", ""), args.min_amrfinder_identity):
            continue
        if not passes_threshold(row.get("% Coverage of reference
sequence", ""), args.min_amrfinder_coverage):
            continue

        raw_gene = row.get("Gene symbol", "").strip()
        if not raw_gene:
            continue
        gene = canonical_gene(raw_gene,
exact_gene_names=args.exact_gene_names)
        hits.append(
            {
                "sample": sample,
                "sequence": row.get("Contig id", ""),
                "tool": "AMRFinder",
                "raw_gene": raw_gene,
                "gene": gene,
                "class": amrfinder_class(row),
                "subclass_or_resistance": row.get("Subclass", ""),
                "start": row.get("Start", ""),
                "end": row.get("Stop", ""),
                "strand": row.get("Strand", ""),
                "percent_coverage": row.get("% Coverage of reference
sequence", ""),
                "percent_identity": row.get("% Identity to reference
sequence", ""),
                "accession": row.get("Accession of closest sequence",
""),
```

```
                "product_or_sequence_name": row.get("Sequence name",
""),
                "element_type": row.get("Element type", ""),
            }
        )
    return hits


def collect_samples(card_rows: list[dict[str, str]], amr_rows:
list[dict[str, str]], nctr_only: bool) -> list[str]:
    samples = set()
    for row in card_rows:
        sample = row.get("#FILE", "").strip()
        if sample and (not nctr_only or sample.startswith("NCTR")):
            samples.add(sample)
    for row in amr_rows:
        sample = row.get("Name", "").strip()
        if sample and (not nctr_only or sample.startswith("NCTR")):
            samples.add(sample)
    return sorted(samples, key=natural_key)


def choose_gene_class(hits: list[dict[str, str]]) -> dict[str, str]:
    by_gene = defaultdict(list)
    for hit in hits:
        by_gene[hit["gene"]].append(hit["class"])

    gene_class = {}
    for gene, classes in by_gene.items():
        classes = sorted(set(classes), key=lambda c:
CLASS_ORDER.get(c, 99))
        gene_class[gene] = classes[0] if classes else "unknown"
    return gene_class


def gene_sort_key(gene: str, gene_class: dict[str, str]) -> tuple:
    cls = gene_class.get(gene, "unknown")
    return (CLASS_ORDER.get(cls, 99), natural_key(gene))


def write_hits_tsv(hits: list[dict[str, str]], path: Path) -> None:
    columns = [
        "sample",
        "sequence",
        "tool",
        "raw_gene",
        "gene",
        "class",
        "subclass_or_resistance",
        "start",
```

```
        "end",
        "strand",
        "percent_coverage",
        "percent_identity",
        "accession",
        "element_type",
        "product_or_sequence_name",
    ]
    with path.open("w", newline="") as handle:
        writer = csv.DictWriter(handle, fieldnames=columns,
delimiter="\t")
        writer.writeheader()
        writer.writerows(hits)


def write_matrix_tsv(samples, genes, status_by_sample_gene, path:
Path) -> None:
    with path.open("w", newline="") as handle:
        writer = csv.writer(handle, delimiter="\t")
        writer.writerow(["sample", *genes])
        for sample in samples:
            writer.writerow([sample,
*[status_by_sample_gene[sample].get(gene, "Absent") for gene in
genes]])


def write_mapping_tsv(hits: list[dict[str, str]], genes: list[str],
path: Path) -> None:
    raw_by_gene_tool = defaultdict(lambda: defaultdict(set))
    class_by_gene = defaultdict(set)
    for hit in hits:
        raw_by_gene_tool[hit["gene"]]
[hit["tool"]].add(hit["raw_gene"])
        class_by_gene[hit["gene"]].add(hit["class"])

    with path.open("w", newline="") as handle:
        writer = csv.writer(handle, delimiter="\t")
        writer.writerow(["gene_column", "classes",
"CARD_raw_gene_names", "AMRFinder_raw_gene_names"])
        for gene in genes:
            writer.writerow(
                [
                    gene,
                    "; ".join(sorted(class_by_gene[gene])),
                    ";
".join(sorted(raw_by_gene_tool[gene].get("CARD", []))),
                    ";
".join(sorted(raw_by_gene_tool[gene].get("AMRFinder", []))),
                ]
            )
```

```
def build_status_matrix(hits: list[dict[str, str]]) -> dict[str,
dict[str, str]]:
    tools_by_sample_gene = defaultdict(lambda: defaultdict(set))
    for hit in hits:
        tools_by_sample_gene[hit["sample"]]
[hit["gene"]].add(hit["tool"])

    status = defaultdict(dict)
    for sample, by_gene in tools_by_sample_gene.items():
        for gene, tools in by_gene.items():
            if {"CARD", "AMRFinder"}.issubset(tools):
                status[sample][gene] = "Both"
            elif "CARD" in tools:
                status[sample][gene] = "CARD"
            elif "AMRFinder" in tools:
                status[sample][gene] = "AMRFinder"
    return status


def write_html(samples, genes, status_by_sample_gene, gene_class,
hits, path: Path, title: str) -> None:
    legend_statuses = ["Both", "CARD", "AMRFinder", "Absent"]
    class_labels = []
    for gene in genes:
        cls = gene_class.get(gene, "unknown")
        if cls not in class_labels:
            class_labels.append(cls)

    parts = [
        "<!doctype html>",
        "<html>",
        "<head>",
        "<meta charset='utf-8'>",
        f"<title>{html.escape(title)}</title>",
        "<style>",
        "body{font-family:Arial,Helvetica,sans-
serif;margin:24px;color:#222;background:#fff}",
        "h1{font-size:20px;margin:0 0 6px 0}",
        ".subtitle{font-size:13px;color:#555;margin:0 0 16px 0}",
        ".wrap{display:flex;gap:18px;align-items:flex-start}",
        ".tablebox{overflow:auto;max-width:calc(100vw -
280px);border:1px solid #d7d7d7}",
        "table{border-collapse:collapse;font-
size:12px;background:#fff}",
        "th,td{border:1px solid #d7d7d7;padding:5px 7px;text-
align:center;white-space:nowrap}",
        "th{background:#f4f4f4;font-
weight:700;position:sticky;top:0;z-index:2}",
```

```
"th.sample,td.sample{position:sticky;left:0;background:#fff;text-
align:left;font-weight:700;z-index:3}",
        "th.sample{background:#f4f4f4;z-index:4}",
        "th.gene{writing-mode:vertical-
rl;transform:rotate(180deg);height:150px;vertical-align:bottom;min-
width:24px}",
        "td.present{color:#fff;font-weight:700}",
        "td.absent{background:#eeeeee;color:#777}",
        ".legend{min-width:245px;border:1px solid
#d7d7d7;padding:12px;background:#fff}",
        ".legend h2{font-size:14px;margin:0 0 10px 0}",
        ".legend-row{display:flex;align-items:center;gap:8px;font-
size:12px;margin:7px 0}",
        ".swatch{width:16px;height:16px;border-radius:3px;border:1px
solid rgba(0,0,0,.18)}",
        ".note{font-size:12px;color:#555;margin-top:14px;max-
width:980px;line-height:1.4}",
        "</style>",
        "</head>",
        "<body>",
        f"<h1>{html.escape(title)}</h1>",
        f"<div class='subtitle'>CARD and AMRFinderPlus hits filtered
to contig3/sequence3. "
        f"{len(samples)} isolates, {len(genes)} gene columns,
{len(hits)} total raw hits.</div>",
    ]

    parts.append("<div class='wrap'><div class='tablebox'><table>")
    parts.append("<thead><tr><th class='sample'>Isolate</th>")
    for gene in genes:
        cls = gene_class.get(gene, "unknown")
        border_color = CLASS_COLORS.get(cls, CLASS_COLORS["unknown"])
        parts.append(
            f"<th class='gene' title='{html.escape(cls)}' "
            f"style='border-bottom:4px solid
{border_color}'>{html.escape(gene)}</th>"
        )
    parts.append("</tr></thead><tbody>")

    for sample in samples:
        parts.append(f"<tr><td class='sample'>{html.escape(sample)}</
td>")
        for gene in genes:
            status = status_by_sample_gene[sample].get(gene, "Absent")
            color = STATUS_COLORS[status]
            if status == "Absent":
                parts.append("<td class='absent'>-</td>")
            else:
                parts.append(f"<td class='present' style='background:
```

```
{color}'>{html.escape(status)}</td>")
        parts.append("</tr>")
    parts.append("</tbody></table></div>")

    parts.append("<div class='legend'><h2>Detection source</h2>")
    for status in legend_statuses:
        parts.append(
            f"<div class='legend-row'><span class='swatch'
style='background:{STATUS_COLORS[status]}'></span>"
            f"<span>{html.escape(status)}</span></div>"
        )
    parts.append("<h2 style='margin-top:16px'>Gene class</h2>")
    for cls in class_labels:
        color = CLASS_COLORS.get(cls, CLASS_COLORS["unknown"])
        parts.append(
            f"<div class='legend-row'><span class='swatch'
style='background:{color}'></span>"
            f"<span>{html.escape(cls)}</span></div>"
        )
    parts.append("</div></div>")

    parts.append(
        "<div class='note'>Default AMRFinderPlus filtering uses
Element type = AMR only. "
        "Run with <code>--include-amrfinder-stress</code> if you also
want AMRFinderPlus stress genes "
        "such as mercury or qac genes. Gene names are lightly
normalized to merge common CARD/AMRFinder "
        "naming differences; see the mapping TSV for the raw names
from each tool.</div>"
    )
    parts.append("</body></html>")
    path.write_text("\n".join(parts))


def main() -> int:
    parser = argparse.ArgumentParser(
        description="Consolidate CARD and AMRFinderPlus AMR genes from
contig3/sequence3 into one colored table."
    )
    parser.add_argument("--xlsx", type=Path, default=DEFAULT_XLSX,
help="Input Excel workbook.")
    parser.add_argument("--outdir", type=Path, default=DEFAULT_OUTDIR,
help="Output directory.")
    parser.add_argument("--card-sheet", default="CARD", help="CARD
sheet name.")
    parser.add_argument("--amrfinder-sheet", default="AMRfinderplus",
help="AMRFinderPlus sheet name.")
    parser.add_argument("--include-non-nctr", action="store_true",
help="Include non-NCTR samples if present.")
```

```
    parser.add_argument("--include-amrfinder-stress",
action="store_true", help="Include AMRFinderPlus STRESS hits.")
    parser.add_argument("--exact-gene-names", action="store_true",
help="Do not normalize gene names between tools.")
    parser.add_argument("--min-card-identity", type=float,
default=0.0, help="Minimum CARD percent identity.")
    parser.add_argument("--min-card-coverage", type=float,
default=0.0, help="Minimum CARD percent coverage.")
    parser.add_argument("--min-amrfinder-identity", type=float,
default=0.0, help="Minimum AMRFinderPlus percent identity.")
    parser.add_argument("--min-amrfinder-coverage", type=float,
default=0.0, help="Minimum AMRFinderPlus percent coverage.")
    parser.add_argument(
        "--title",
        default="CARD and AMRFinderPlus AMR genes detected in contig3/
sequence3",
        help="Title for the HTML table.",
    )
    args = parser.parse_args()
    args.nctr_only = not args.include_non_nctr

    if not args.xlsx.exists():
        raise SystemExit(f"Cannot find workbook: {args.xlsx}")

    args.outdir.mkdir(parents=True, exist_ok=True)
    card_rows = read_xlsx_sheet(args.xlsx, args.card_sheet)
    amrfinder_rows = read_xlsx_sheet(args.xlsx, args.amrfinder_sheet)

    card_hits = extract_card_hits(card_rows, args)
    amrfinder_hits = extract_amrfinder_hits(amrfinder_rows, args)
    hits = sorted(card_hits + amrfinder_hits, key=lambda h:
(natural_key(h["sample"]), h["gene"], h["tool"], h["start"]))

    samples = collect_samples(card_rows, amrfinder_rows,
nctr_only=args.nctr_only)
    gene_class = choose_gene_class(hits)
    genes = sorted({hit["gene"] for hit in hits}, key=lambda gene:
gene_sort_key(gene, gene_class))
    status_by_sample_gene = build_status_matrix(hits)

    hits_tsv = args.outdir /
"contig3_card_amrfinder_combined_hits.tsv"
    matrix_tsv = args.outdir /
"contig3_card_amrfinder_combined_matrix.tsv"
    mapping_tsv = args.outdir /
"contig3_card_amrfinder_gene_name_mapping.tsv"
    html_path = args.outdir /
"contig3_card_amrfinder_combined_colored_table.html"

    write_hits_tsv(hits, hits_tsv)
```

```
    write_matrix_tsv(samples, genes, status_by_sample_gene,
matrix_tsv)
    write_mapping_tsv(hits, genes, mapping_tsv)
    write_html(samples, genes, status_by_sample_gene, gene_class,
hits, html_path, args.title)

    print(f"CARD sheet rows scanned: {len(card_rows)}")
    print(f"AMRFinderPlus sheet rows scanned: {len(amrfinder_rows)}")
    print(f"CARD contig3 hits kept: {len(card_hits)}")
    print(f"AMRFinderPlus contig3 hits kept: {len(amrfinder_hits)}")
    print(f"Samples included: {len(samples)}")
    print(f"Consolidated gene columns: {len(genes)}")
    print("Saved:")
    print(f"  {html_path}")
    print(f"  {matrix_tsv}")
    print(f"  {hits_tsv}")
    print(f"  {mapping_tsv}")
    return 0


if __name__ == "__main__":
    sys.exit(main())
```


Script 22: search_resistance_genes_in_contig3_amr_negative_isolates.py
Source file:
search_resistance_genes_in_contig3_amr_negative_isolates.py
Line count: 619


```python
#!/usr/bin/env python3
"""
Find AMR and heavy-metal resistance genes in NCTR isolates that had no
AMR
genes detected in contig3.

Default behavior:
  1. Read contig3_amr_presence_absence.tsv.
  2. Identify isolates where all contig3 AMR calls are "absent".
  3. Search the other GenBank contigs from those isolates, excluding
contig3.
  4. Write a hits table, presence/absence matrix, and colored HTML
table.

This is annotation-based. It searches GenBank CDS /gene, /product, /
note, and
/function qualifiers. Confirm important calls with AMRFinderPlus/
```

```
ResFinder/CARD
if you need database-standard AMR annotation.
"""

from __future__ import annotations

import argparse
import csv
import glob
import html
import re
from dataclasses import dataclass
from pathlib import Path


PROJECT_DIR = Path("<project_directory>")


AMR_GENE_RE = re.compile(
    r"^(bla|tet|sul|dfr|dhfr|aad|aac|aph|ant|str|cat|cml|flo|qnr|oqx|
mcr|mph|erm|lnu|arr|fos|rmt|armA|npmA|qac|ble|bcr|ere)",
    re.I,
)

AMR_PRODUCT_WORDS = [
    "beta-lactamase",
    "new delhi metallo",
    "bleomycin resistance",
    "tetracycline resistance",
    "tetracycline efflux",
    "tetracycline repressor",
    "sulfonamide resistance",
    "dihydropteroate synthase",
    "trimethoprim",
    "dihydrofolate reductase",
    "aminoglycoside",
    "adenylyltransferase",
    "nucleotidyltransferase",
    "phosphotransferase",
    "16s rrna methyl",
    "streptomycin",
    "spectinomycin",
    "chloramphenicol",
    "florfenicol",
    "phenicol",
    "quinolone resistance",
    "macrolide",
    "erythromycin esterase",
    "rifampicin adp-ribosyl",
    "rifamycin adp-ribosyl",
```

```
    "fosfomycin resistance",
    "colistin resistance",
    "bicyclomycin resistance",
    "quaternary ammonium compound",
    "multidrug efflux",
]

HMR_GENE_RE = re.compile(
    r"^(pco|sil|cus|cop|cue|cut|ter|ars|mer|czc|czr|znt|zit|cad|chr|
rcn|nre)",
    re.I,
)

HMR_PRODUCT_WORDS = [
    "copper",
    "silver",
    "tellurium",
    "tellurite",
    "arsenic",
    "arsenate",
    "arsenite",
    "mercury",
    "mercuric",
    "cadmium",
    "cobalt",
    "zinc",
    "nickel",
    "chromate",
    "heavy metal",
    "metal resistance",
    "metal efflux",
]

CLASS_ORDER = [
    "AMR: beta-lactam",
    "AMR: aminoglycoside",
    "AMR: trimethoprim",
    "AMR: sulfonamide",
    "AMR: tetracycline",
    "AMR: phenicol",
    "AMR: macrolide",
    "AMR: rifamycin",
    "AMR: bleomycin",
    "AMR: bicyclomycin",
    "AMR: qac/efflux",
    "HMR: copper/silver",
    "HMR: tellurium",
    "HMR: arsenic",
    "HMR: mercury",
    "HMR: cadmium/zinc/cobalt",
```

```
    "HMR: chromate",
    "HMR: nickel/cobalt",
    "HMR: other metal",
    "other",
]

COLORS = {
    "AMR: beta-lactam": "#b8324c",
    "AMR: aminoglycoside": "#2d7fb8",
    "AMR: trimethoprim": "#9a6f2d",
    "AMR: sulfonamide": "#568b2f",
    "AMR: tetracycline": "#d4861d",
    "AMR: phenicol": "#5f3b9a",
    "AMR: macrolide": "#c4517d",
    "AMR: rifamycin": "#8a6a35",
    "AMR: bleomycin": "#7d5fb2",
    "AMR: bicyclomycin": "#159a7d",
    "AMR: qac/efflux": "#6b6b6b",
    "HMR: copper/silver": "#009e73",
    "HMR: tellurium": "#00a7b5",
    "HMR: arsenic": "#8b6f00",
    "HMR: mercury": "#7570b3",
    "HMR: cadmium/zinc/cobalt": "#a6761d",
    "HMR: chromate": "#666666",
    "HMR: nickel/cobalt": "#1b9e77",
    "HMR: other metal": "#66a61e",
    "other": "#4f7f6f",
    "absent": "#eeeeee",
}


@dataclass(frozen=True)
class Hit:
    isolate: str
    contig: str
    row_label: str
    category: str
    gene: str
    gene_class: str
    location: str
    gene_annotation: str
    locus_tag: str
    product: str
    note: str
    matched_text: str
    source_file: Path


def parse_args() -> argparse.Namespace:
    parser = argparse.ArgumentParser(
```

```
        description="Search AMR/HMR genes in NCTR isolates with no AMR
detected in contig3."
    )
    parser.add_argument("--project-dir", type=Path,
default=PROJECT_DIR)
    parser.add_argument(
        "--contig3-matrix",
        type=Path,
        default=PROJECT_DIR / "contig3_amr_results" /
"contig3_amr_presence_absence.tsv",
        help="contig3 AMR presence/absence matrix used to identify
AMR-negative isolates.",
    )
    parser.add_argument(
        "--isolates",
        default="",
        help="Optional comma-separated isolate list, e.g.
NCTR718,NCTR719,NCTR938. If set, overrides --contig3-matrix.",
    )
    parser.add_argument(
        "--include-contig3",
        action="store_true",
        help="Also scan contig3. Default is to exclude contig3 and
search other contigs only.",
    )
    parser.add_argument(
        "--outdir",
        type=Path,
        default=PROJECT_DIR /
"contig3_amr_negative_isolates_resistance_scan",
    )
    parser.add_argument("--prefix",
default="contig3_amr_negative_other_resistance")
    return parser.parse_args()


def clean_text(value: str) -> str:
    return re.sub(r"\s+", " ", value.replace("\n", " ")).strip()


def isolate_from_sample(sample: str) -> str:
    return re.sub(r"_contig[_-]?\d+$", "", sample, flags=re.I)


def contig_from_path(path: Path) -> str:
    match = re.search(r"(contig[_-]?\d+)", path.stem, flags=re.I)
    return match.group(1).replace("_", "")
    return path.stem
```

```
def read_negative_isolates(matrix_path: Path) -> list[str]:
    if not matrix_path.exists():
        raise FileNotFoundError(matrix_path)
    negatives: list[str] = []
    with matrix_path.open(newline="") as handle:
        reader = csv.DictReader(handle, delimiter="\t")
        for row in reader:
            sample = row.get("isolate") or row.get("sample")
            if not sample:
                continue
            calls = [value.strip().lower() for key, value in
row.items() if key not in {"isolate", "sample"}]
            if calls and all(value in {"absent", "0", "", "false",
"not detected"} for value in calls):
                negatives.append(isolate_from_sample(sample))
    return sorted(set(negatives))


def preferred_gbk_paths(project_dir: Path, isolate: str,
include_contig3: bool) -> list[Path]:
    candidates = []
    patterns = [
        project_dir / f"{isolate}_contig*.gbk",
        project_dir / "all_contigs" / f"{isolate}_contig*.gbk",
        project_dir / "gbk" / "all_contigs" / f"{isolate}
_contig*.gbk",
        project_dir / "gbk" / f"{isolate}_contig*_prokka" /
f"{isolate}_contig*.gbk",
    ]
    for pattern in patterns:
        candidates.extend(Path(p) for p in glob.glob(str(pattern)))

    by_stem: dict[str, Path] = {}
    for path in candidates:
        if not include_contig3 and re.search(r"contig[_-]?3",
path.stem, flags=re.I):
            continue
        stem = path.stem
        if stem not in by_stem:
            by_stem[stem] = path
            continue
        old = by_stem[stem]
        if path.parent == project_dir:
            by_stem[stem] = path
        elif old.parent != project_dir and "all_contigs" in str(path):
            by_stem[stem] = path
    return [by_stem[key] for key in sorted(by_stem)]


def parse_cds_blocks(path: Path) -> list[tuple[str, str]]:
```

```
    blocks: list[tuple[str, str]] = []
    current: list[str] = []
    location = ""
    in_cds = False
    for line in path.read_text(errors="replace").splitlines():
        if line.startswith("     CDS"):
            if current:
                blocks.append((location, "\n".join(current)))
            current = [line]
            location = line[21:].strip()
            in_cds = True
            continue
        if in_cds and re.match(r"^     \S", line) and not
line.startswith("                     "):
            blocks.append((location, "\n".join(current)))
            current = []
            location = ""
            in_cds = False
            continue
        if in_cds:
            current.append(line)
    if current:
        blocks.append((location, "\n".join(current)))
    return blocks


def qualifier_values(block: str, key: str) -> list[str]:
    return [clean_text(value) for value in re.findall(rf'/
{re.escape(key)}="([^"]*)"', block, flags=re.S)]


def normalize_label(label: str) -> str:
    label = clean_text(label)
    label = re.sub(r"\bbla[-_ ]?(TEM|CMY|NDM|SHV|OXA)[-_ ]?([A-Za-
z0-9]*)\b", lambda m: "bla" + m.group(1).upper() + (("-" + m.group(2))
if m.group(2) else ""), label, flags=re.I)
    label = re.sub(r"\bCMY[-_ ]?(\d+)\b", r"blaCMY-\1", label,
flags=re.I)
    label = re.sub(r"\bNDM[-_ ]?(\d+)\b", r"blaNDM-\1", label,
flags=re.I)
    label = re.sub(r"\bCmlA(\d+)\b", r"cmlA\1", label, flags=re.I)
    label = re.sub(r"_(\d+)$", "", label)
    label = re.sub(r"\s+", "", label)
    if re.fullmatch(r"dhfrI", label, flags=re.I):
        return "dfrA1/dhfrI"
    return label


def infer_amr_label(genes: list[str], product_note: str) -> str:
    beta_match = re.search(r"\bbeta[- ]lactamase\s+(TEM|CMY|NDM|SHV|
```

```
OXA)[-_ ]?([A-Za-z0-9]*)\b", product_note, flags=re.I)
    if beta_match:
        family = beta_match.group(1).upper()
        suffix = beta_match.group(2)
        return "bla" + family + (("-" + suffix) if suffix else "")

    patterns = [
        r"\bbla[-_ ]?(?:TEM|CMY|NDM|SHV|OXA)[-_ ]?[A-Za-z0-9]*\b",
        r"\b(?:CMY|NDM|TEM|SHV|OXA)[-_ ]?\d+\b",
        r"\btet\([A-Za-z0-9]+\)\b",
        r"\btet[A-Z0-9]{1,3}\b",
        r"\bsul[123](?:_\d+)?\b",
        r"\bdhfrI\b",
        r"\bdfrA\d+\b",
        r"\baadA[A-Za-z0-9_-]*\b",
        r"\baadB\b",
        r"\baac\([^)]+\)-[A-Za-z0-9]+",
        r"\baph\([^)]+\)-[A-Za-z0-9]+",
        r"\bant\([^)]+\)-[A-Za-z0-9]+",
        r"\bneo\b",
        r"\brmtB\b",
        r"\bfloR\b",
        r"\bcat[A-Za-z0-9_-]*\b",
        r"\bcmlA\d+\b",
        r"\bqnr[A-Za-z0-9]+\b",
        r"\bmcr-?\d+\b",
        r"\bereC\b",
        r"\barr-?3\b",
        r"\bbleMBL\b",
        r"\bbcr\b",
        r"\bqac[A-Za-z0-9_-]*\b",
        r"\bmdtL\b",
        r"\btap\b",
    ]
    for pattern in patterns:
        match = re.search(pattern, product_note, flags=re.I)
        if match:
            return normalize_label(match.group(0))

    for gene in genes:
        if gene and AMR_GENE_RE.search(gene):
            return normalize_label(gene)

    low = product_note.lower()
    if "aminoglycoside" in low and "adenylyltransferase" in low:
        return "aadA-like"
    if "aminoglycoside" in low and "phosphotransferase" in low:
        return "aph-like"
    if "dihydrofolate reductase" in low:
        return "dfrA1/dhfrI"
```

```
    return "unclassified_AMR"


def infer_hmr_label(genes: list[str], product_note: str) -> str:
    for gene in genes:
        if gene and HMR_GENE_RE.search(gene):
            return normalize_label(gene)
    patterns = [
        r"\bpco[A-Za-z0-9]+\b",
        r"\bsil[A-Za-z0-9]+\b",
        r"\bcus[A-Za-z0-9]+\b",
        r"\bcop[A-Za-z0-9]+\b",
        r"\bter[A-Za-z0-9]+\b",
        r"\bars[A-Za-z0-9]+\b",
        r"\bmer[A-Za-z0-9]+\b",
        r"\bczc[A-Za-z0-9]+\b",
        r"\bznt[A-Za-z0-9]+\b",
        r"\bchr[A-Za-z0-9]+\b",
        r"\brcn[A-Za-z0-9]+\b",
        r"\bcue[A-Za-z0-9]+\b",
        r"\bcut[A-Za-z0-9]+\b",
    ]
    for pattern in patterns:
        match = re.search(pattern, product_note, flags=re.I)
        if match:
            return normalize_label(match.group(0))
    low = product_note.lower()
    for word, label in [
        ("copper", "copper_resistance"),
        ("silver", "silver_resistance"),
        ("tellur", "tellurium_resistance"),
        ("arsen", "arsenic_resistance"),
        ("mercur", "mercury_resistance"),
        ("cadmium", "cadmium_resistance"),
        ("zinc", "zinc_resistance"),
        ("chromate", "chromate_resistance"),
        ("nickel", "nickel_resistance"),
    ]:
        if word in low:
            return label
    return "metal_resistance"


def amr_class(label: str, text: str) -> str:
    low_label = label.lower()
    low_text = text.lower()
    if low_label.startswith("bla") or "beta-lactamase" in low_text:
        return "AMR: beta-lactam"
    if low_label.startswith(("aad", "aac", "aph", "ant", "str", "rmt",
"neo")) or "aminoglycoside" in low_text:
```

```
        return "AMR: aminoglycoside"
    if low_label.startswith(("dfr", "dhfr")) or "dihydrofolate
reductase" in low_text or "trimethoprim" in low_text:
        return "AMR: trimethoprim"
    if low_label.startswith("sul") or "dihydropteroate" in low_text or
"sulfonamide" in low_text:
        return "AMR: sulfonamide"
    if low_label.startswith("tet") or "tetracycline" in low_text:
        return "AMR: tetracycline"
    if low_label.startswith(("cml", "cat", "flo")) or
"chloramphenicol" in low_text or "phenicol" in low_text:
        return "AMR: phenicol"
    if low_label.startswith(("erm", "mph", "lnu", "ere")) or
"erythromycin" in low_text or "macrolide" in low_text:
        return "AMR: macrolide"
    if low_label.startswith("arr") or "rifamycin" in low_text or
"rifampicin" in low_text:
        return "AMR: rifamycin"
    if low_label.startswith("ble") or "bleomycin" in low_text:
        return "AMR: bleomycin"
    if low_label.startswith("bcr") or "bicyclomycin" in low_text:
        return "AMR: bicyclomycin"
    if low_label.startswith(("qac", "mdt", "tap")) or "efflux" in
low_text or "quaternary ammonium" in low_text:
        return "AMR: qac/efflux"
    return "other"


def hmr_class(label: str, text: str) -> str:
    low = (label + " " + text).lower()
    if any(word in low for word in ["pco", "sil", "cus", "cop", "cue",
"cut", "copper", "silver"]):
        return "HMR: copper/silver"
    if "ter" in low or "tellur" in low:
        return "HMR: tellurium"
    if "ars" in low or "arsenic" in low or "arsenate" in low or
"arsenite" in low:
        return "HMR: arsenic"
    if "mer" in low or "mercur" in low:
        return "HMR: mercury"
    if any(word in low for word in ["czc", "czr", "znt", "zit",
"cadmium", "zinc", "cobalt"]):
        return "HMR: cadmium/zinc/cobalt"
    if "chr" in low or "chromate" in low:
        return "HMR: chromate"
    if "rcn" in low or "nre" in low or "nickel" in low:
        return "HMR: nickel/cobalt"
    return "HMR: other metal"
```

```
def find_hits_in_file(isolate: str, path: Path) -> list[Hit]:
    hits: list[Hit] = []
    seen: set[tuple[str, str, str]] = set()
    contig = contig_from_path(path)
    row_label = f"{isolate}_{contig}"
    for location, block in parse_cds_blocks(path):
        genes = qualifier_values(block, "gene")
        locus_tags = qualifier_values(block, "locus_tag")
        products = qualifier_values(block, "product")
        notes = qualifier_values(block, "note") +
qualifier_values(block, "function")
        searchable = clean_text(" | ".join(genes + locus_tags +
products + notes))
        low = searchable.lower()
        is_amr = any(AMR_GENE_RE.search(gene) for gene in genes) or
any(word in low for word in AMR_PRODUCT_WORDS)
        is_hmr = any(HMR_GENE_RE.search(gene) for gene in genes) or
any(word in low for word in HMR_PRODUCT_WORDS)
        if not is_amr and not is_hmr:
            continue
        category = "AMR" if is_amr else "HMR"
        label = infer_amr_label(genes, searchable) if is_amr else
infer_hmr_label(genes, searchable)
        cls = amr_class(label, searchable) if is_amr else
hmr_class(label, searchable)
        key = (category, label, location)
        if key in seen:
            continue
        seen.add(key)
        hits.append(
            Hit(
                isolate=isolate,
                contig=contig,
                row_label=row_label,
                category=category,
                gene=label,
                gene_class=cls,
                location=location,
                gene_annotation=";".join(genes) if genes else "-",
                locus_tag=";".join(locus_tags) if locus_tags else "-",
                product=";".join(products) if products else "-",
                note=";".join(notes) if notes else "-",
                matched_text=searchable,
                source_file=path,
            )
        )
    return hits


def sort_gene(gene: str, class_by_gene: dict[str, str]) -> tuple[int,
```

```
str]:
    cls = class_by_gene.get(gene, "other")
    idx = CLASS_ORDER.index(cls) if cls in CLASS_ORDER else
len(CLASS_ORDER)
    return (idx, gene.lower())


def write_hits(path: Path, hits: list[Hit]) -> None:
    with path.open("w", newline="") as handle:
        writer = csv.writer(handle, delimiter="\t")
        writer.writerow([
            "isolate",
            "contig",
            "row_label",
            "category",
            "gene",
            "class",
            "location",
            "gene_annotation",
            "locus_tag",
            "product",
            "note",
            "matched_text",
            "source_file",
        ])
        for hit in hits:
            writer.writerow([
                hit.isolate,
                hit.contig,
                hit.row_label,
                hit.category,
                hit.gene,
                hit.gene_class,
                hit.location,
                hit.gene_annotation,
                hit.locus_tag,
                hit.product,
                hit.note,
                hit.matched_text,
                str(hit.source_file),
            ])


def write_matrix(path: Path, rows: list[str], genes: list[str],
present: dict[str, set[str]]) -> None:
    with path.open("w", newline="") as handle:
        writer = csv.writer(handle, delimiter="\t")
        writer.writerow(["isolate_contig"] + genes)
        for row in rows:
            writer.writerow([row] + ["present" if gene in present[row]
```

```
else "absent" for gene in genes])


def write_html(path: Path, title: str, rows_order: list[str], genes:
list[str], class_by_gene: dict[str, str], present: dict[str,
set[str]]) -> None:
    rows = [
        "<!doctype html>",
        "<meta charset='utf-8'>",
        f"<title>{html.escape(title)}</title>",
        "<style>body{font-family:Arial,Helvetica,sans-
serif;margin:18px;color:#222}h2{font-size:18px;margin:0 0 8px}p{font-
size:12px;margin:0 0 12px}.figure-wrap{display:flex;align-items:flex-
start;gap:16px;width:max-content}table{border-collapse:collapse}
th,td{border:1px solid #d0d0d0;padding:4px 6px;text-align:center;font-
size:12px}th.gene{writing-mode:vertical-
rl;transform:rotate(180deg);height:120px;min-width:24px}
td.sample{text-align:right;font-weight:600;background:#fafafa;white-
space:nowrap}.absent{background:#eee;color:#777}.legend{display:flex;f
lex-direction:column;gap:7px;margin:0;padding:10px 12px;border:1px
solid #d0d0d0;background:#fafafa;min-width:170px;font-
size:12px}.legend-title{font-weight:700;margin-
bottom:3px}.swatch{display:inline-block;width:14px;height:14px;margin-
right:7px;vertical-align:-2px;border:1px solid #aaa}</style>",
        f"<h2>{html.escape(title)}</h2>",
        "<p>Rows are other contigs from isolates with no AMR genes
detected in contig3. Filled cells indicate AMR or heavy-metal
resistance annotations.</p>",
        "<div class='figure-wrap'><table><thead><tr><th>isolate/
contig</th>",
    ]
    for gene in genes:
        rows.append(f"<th class='gene'>{html.escape(gene)}</th>")
    rows.append("</tr></thead><tbody>")
    for row in rows_order:
        rows.append(f"<tr><td class='sample'>{html.escape(row)}</td>")
        for gene in genes:
            if gene in present[row]:
                color = COLORS.get(class_by_gene.get(gene, "other"),
COLORS["other"])
                rows.append(f"<td style='background:
{color};color:white;font-weight:700'>+</td>")
            else:
                rows.append("<td class='absent'>-</td>")
        rows.append("</tr>")
    rows.append("</tbody></table><div class='legend'><div
class='legend-title'>Resistance class</div>")
    for cls in CLASS_ORDER:
        if cls in set(class_by_gene.values()):
            rows.append(f"<span><span class='swatch'
```

```
style='background:{COLORS[cls]}'></span>{html.escape(cls)}</span>")
    rows.append("<span><span class='swatch' style='background:#eee'></
span>absent/not detected</span></div></div>")
    path.write_text("\n".join(rows))


def main() -> int:
    args = parse_args()
    if args.isolates:
        isolates = sorted({item.strip() for item in
args.isolates.split(",") if item.strip()})
    else:
        isolates = read_negative_isolates(args.contig3_matrix)
    if not isolates:
        raise SystemExit("No contig3 AMR-negative isolates found.")

    all_hits: list[Hit] = []
    scanned_rows: list[str] = []
    scanned_files: list[tuple[str, Path]] = []
    for isolate in isolates:
        paths = preferred_gbk_paths(args.project_dir, isolate,
args.include_contig3)
        for path in paths:
            row = f"{isolate}_{contig_from_path(path)}"
            scanned_rows.append(row)
            scanned_files.append((row, path))
            all_hits.extend(find_hits_in_file(isolate, path))

    scanned_rows = sorted(set(scanned_rows))
    class_by_gene: dict[str, str] = {}
    for hit in all_hits:
        class_by_gene.setdefault(hit.gene, hit.gene_class)
    genes = sorted(class_by_gene, key=lambda gene: sort_gene(gene,
class_by_gene))
    present = {row: set() for row in scanned_rows}
    for hit in all_hits:
        present.setdefault(hit.row_label, set()).add(hit.gene)

    args.outdir.mkdir(parents=True, exist_ok=True)
    isolates_path = args.outdir / f"{args.prefix}_target_isolates.txt"
    files_path = args.outdir / f"{args.prefix}_files_scanned.tsv"
    hits_path = args.outdir / f"{args.prefix}_hits.tsv"
    matrix_path = args.outdir / f"{args.prefix}_presence_absence.tsv"
    html_path = args.outdir / f"{args.prefix}_colored_table.html"

    isolates_path.write_text("\n".join(isolates) + "\n")
    with files_path.open("w", newline="") as handle:
        writer = csv.writer(handle, delimiter="\t")
        writer.writerow(["row_label", "source_file"])
        writer.writerows((row, str(path)) for row, path in
```

```
scanned_files)
    write_hits(hits_path, all_hits)
    write_matrix(matrix_path, scanned_rows, genes, present)
    write_html(
        html_path,
        "AMR and heavy-metal resistance genes outside contig3 AMR-
negative isolates",
        scanned_rows,
        genes,
        class_by_gene,
        present,
    )

    print("Target isolates with no contig3 AMR calls:")
    print("  " + ", ".join(isolates))
    print(f"Files scanned: {len(scanned_files)}")
    print(f"Resistance genes/classes detected: {len(genes)}")
    print("Outputs:")
    print(f"  {isolates_path}")
    print(f"  {files_path}")
    print(f"  {hits_path}")
    print(f"  {matrix_path}")
    print(f"  {html_path}")
    return 0


if __name__ == "__main__":
    raise SystemExit(main())
```


Script 23: make_mer_contig2_table.py
Source file: make_mer_contig2_table.py
Line count: 62


```python
#!/usr/bin/env python3
from pathlib import Path
import re, csv, html

PROJECT = Path("<project_directory>")
GBK_DIR = PROJECT / "all_contigs"
OUTDIR = PROJECT / "mer_contig2_results"
OUTDIR.mkdir(exist_ok=True)

MER_GENES = ["merA", "merC", "merP", "merT", "merR"]

rows = []
```

```
for gbk in sorted(GBK_DIR.glob("NCTR*_contig2.gbk")):
    isolate = gbk.stem.replace("_contig2", "")
    text = gbk.read_text(errors="replace")

    row = {"isolate": isolate}
    for gene in MER_GENES:
        row[gene] = "present" if re.search(rf'\b{gene}\b', text, re.I)
else "absent"
    rows.append(row)

tsv = OUTDIR / "mer_contig2_presence_absence.tsv"
with tsv.open("w", newline="") as f:
    writer = csv.DictWriter(f, fieldnames=["isolate"] + MER_GENES,
delimiter="\t")
    writer.writeheader()
    writer.writerows(rows)

html_path = OUTDIR / "mer_contig2_colored_table.html"
parts = [
    "<!doctype html><meta charset='utf-8'>",
    "<title>Mercury resistance genes in contig2</title>",
    "<style>",
    "body{font-family:Arial;margin:24px;color:#222}",
    "table{border-collapse:collapse}",
    "th,td{border:1px solid #ccc;padding:7px 12px;text-
align:center;font-size:13px}",
    "th{background:#f3f3f3}",
    "td.sample{text-align:right;font-weight:700;background:#fafafa}",
    ".present{background:#7570b3;color:white;font-weight:700}",
    ".absent{background:#eeeeee;color:#777}",
    "</style>",
    "<h2>Mercury resistance genes in NCTR contig2 plasmids</h2>",
    "<table><thead><tr><th>isolate</th>",
]

for gene in MER_GENES:
    parts.append(f"<th>{html.escape(gene)}</th>")
parts.append("</tr></thead><tbody>")

for row in rows:
    parts.append(f"<tr><td
class='sample'>{html.escape(row['isolate'])}</td>")
    for gene in MER_GENES:
        status = row[gene]
        symbol = "+" if status == "present" else "-"
        parts.append(f"<td class='{status}'>{symbol}</td>")
    parts.append("</tr>")

parts.append("</tbody></table>")
html_path.write_text("\n".join(parts))
```

```
print("Saved:")
print(tsv)
print(html_path)
```

Script 24: make_mer_contig2_table_with_references.py
Source file: make_mer_contig2_table_with_references.py
Line count: 261

```python
#!/usr/bin/env python3
from pathlib import Path
import csv
import html
import re
import shutil


PROJECT = Path("<project_directory>")
GBK_DIR = PROJECT / "all_contigs"
OUTDIR = PROJECT / "mer_contig2_results"

# NCTR contig2 plasmids plus reference plasmids.
NCTR_GBKS = sorted(GBK_DIR.glob("NCTR*_contig2.gbk"))
REFERENCE_GBKS = [
    ("R478", GBK_DIR / "R478.gbk"),
    ("pSH111_227", PROJECT / "pSH111_227.gbk"),
]

# These are the common mer genes expected in mercury resistance operons.
# The script will add any additional mer* genes detected in the GenBank files.
PREFERRED_MER_ORDER = [
    "merA",
    "merC",
    "merP",
    "merT",
    "merR",
    "merD",
    "merE",
    "merB",
    "merF",
    "merG",
]
```

```
def natural_key(text):
    return tuple(int(part) if part.isdigit() else part.lower() for
part in re.split(r"(\d+)", text))


def clean_qualifier(value):
    return re.sub(r"\s+", " ", value or "").strip()


def get_qualifier(feature_block, name):
    match = re.search(rf'/{re.escape(name)}="([^"]*)"', feature_block,
re.S)
    if not match:
        return ""
    return clean_qualifier(match.group(1))


def parse_location(location):
    numbers = [int(n) for n in re.findall(r"\d+", location)]
    if not numbers:
        return "", "", ""
    strand = "-" if "complement" in location else "+"
    return str(min(numbers)), str(max(numbers)), strand


def iter_features(gbk_text):
    pattern = re.compile(
        r"^     (?P<type>gene|CDS)\s+(?P<location>.+?)\n"
        r"(?P<body>.*?)(?=^     \S|\nORIGIN|\Z)",
        re.M | re.S,
    )
    for match in pattern.finditer(gbk_text):
        yield match.group("type"), match.group("location").strip(),
match.group("body")


def extract_mer_hits(sample, gbk_path):
    text = gbk_path.read_text(errors="replace")
    mer_hits = []
    mercury_associated_non_mer = []
    seen_mer = set()
    seen_non_mer = set()

    for feature_type, location, body in iter_features(text):
        gene = get_qualifier(body, "gene")
        product = get_qualifier(body, "product")
        locus_tag = get_qualifier(body, "locus_tag")
        start, end, strand = parse_location(location)
```

```
        if re.fullmatch(r"mer[A-Za-z0-9_-]+", gene or "", re.I):
            gene = gene[:3].lower() + gene[3:]
            key = (sample, gene.lower(), start, end, strand, product)
            if key not in seen_mer:
                seen_mer.add(key)
                mer_hits.append(
                    {
                        "sample": sample,
                        "source_file": str(gbk_path),
                        "feature_type": feature_type,
                        "gene": gene,
                        "start": start,
                        "end": end,
                        "strand": strand,
                        "locus_tag": locus_tag,
                        "product": product,
                    }
                )

        # Keep this separate from the colored mer table. For example,
pSH111_227
        # has silP annotated as a metal-transporting ATPase that
mentions mercury,
        # but silP is not a mer operon gene.
        if "mercur" in product.lower() and not re.fullmatch(r"mer[A-
Za-z0-9_-]+", gene or "", re.I):
            key = (sample, gene, start, end, strand, product)
            if key not in seen_non_mer:
                seen_non_mer.add(key)
                mercury_associated_non_mer.append(
                    {
                        "sample": sample,
                        "source_file": str(gbk_path),
                        "feature_type": feature_type,
                        "gene": gene or locus_tag or
"unnamed_feature",
                        "start": start,
                        "end": end,
                        "strand": strand,
                        "locus_tag": locus_tag,
                        "product": product,
                    }
                )

    return mer_hits, mercury_associated_non_mer


def ordered_mer_genes(detected_genes):
    preferred_lower = {gene.lower(): idx for idx, gene in
enumerate(PREFERRED_MER_ORDER)}
```

```
    def key(gene):
        low = gene.lower()
        if low in preferred_lower:
            return (0, preferred_lower[low])
        return (1, natural_key(gene))

    return sorted(detected_genes, key=key)


def write_tsv(path, rows, fieldnames):
    with path.open("w", newline="") as handle:
        writer = csv.DictWriter(handle, fieldnames=fieldnames,
delimiter="\t")
        writer.writeheader()
        writer.writerows(rows)


def build_html(rows, mer_genes, output_html):
    parts = [
        "<!doctype html>",
        "<html>",
        "<head>",
        "<meta charset='utf-8'>",
        "<title>Mercury resistance genes in contig2 and reference
plasmids</title>",
        "<style>",
        "body{font-family:Arial,Helvetica,sans-
serif;margin:24px;color:#222;background:#fff}",
        "h2{font-size:20px;margin:0 0 6px 0}",
        ".note{font-size:13px;color:#555;margin:0 0 16px 0}",
        "table{border-collapse:collapse}",
        "th,td{border:1px solid #ccc;padding:7px 12px;text-
align:center;font-size:13px}",
        "th{background:#f3f3f3}",
        "td.sample{text-align:right;font-
weight:700;background:#fafafa}",
        "td.reference{background:#fff7e6}",
        ".present{background:#7570b3;color:white;font-weight:700}",
        ".absent{background:#eeeeee;color:#777}",
        "</style>",
        "</head>",
        "<body>",
        "<h2>Mercury resistance genes in NCTR contig2 plasmids and
reference plasmids</h2>",
        "<p class='note'>Rows R478 and pSH111_227 are reference
plasmids. "
        "A colored cell indicates that a mer* gene qualifier was
detected in the GenBank file.</p>",
        "<table><thead><tr><th>isolate/plasmid</th>",
```

```
    ]

    for gene in mer_genes:
        parts.append(f"<th>{html.escape(gene)}</th>")
    parts.append("</tr></thead><tbody>")

    for row in rows:
        sample_class = "sample reference" if row["row_type"] ==
"reference" else "sample"
        parts.append(f"<tr><td
class='{sample_class}'>{html.escape(row['sample'])}</td>")
        for gene in mer_genes:
            status = row[gene]
            symbol = "+" if status == "present" else "−"
            parts.append(f"<td class='{status}'>{symbol}</td>")
        parts.append("</tr>")

    parts.append("</tbody></table>")
    parts.append("</body></html>")
    output_html.write_text("\n".join(parts))


def main():
    OUTDIR.mkdir(parents=True, exist_ok=True)

    sample_files = []
    for gbk in NCTR_GBKS:
        sample_files.append((gbk.stem.replace("_contig2", ""),
"NCTR_contig2", gbk))
    for sample, gbk in REFERENCE_GBKS:
        if gbk.exists():
            sample_files.append((sample, "reference", gbk))
        else:
            print(f"Warning: reference file not found: {gbk}")

    all_mer_hits = []
    all_non_mer_mercury_hits = []
    present_by_sample = {}

    for sample, row_type, gbk in sample_files:
        mer_hits, non_mer_hits = extract_mer_hits(sample, gbk)
        all_mer_hits.extend(mer_hits)
        all_non_mer_mercury_hits.extend(non_mer_hits)
        present_by_sample[sample] = {hit["gene"] for hit in mer_hits}

    mer_genes = ordered_mer_genes({hit["gene"] for hit in
all_mer_hits})

    table_rows = []
    for sample, row_type, gbk in sample_files:
```

```
        row = {
            "sample": sample,
            "row_type": row_type,
            "source_file": str(gbk),
        }
        for gene in mer_genes:
            row[gene] = "present" if gene in
present_by_sample.get(sample, set()) else "absent"
        table_rows.append(row)

    presence_tsv = OUTDIR /
"mer_contig2_presence_absence_with_references.tsv"
    hits_tsv = OUTDIR / "mer_contig2_hits_with_references.tsv"
    non_mer_tsv = OUTDIR /
"mercury_associated_non_mer_hits_with_references.tsv"
    html_path = OUTDIR /
"mer_contig2_colored_table_with_references.html"

    write_tsv(presence_tsv, table_rows, ["sample", "row_type",
"source_file", *mer_genes])
    write_tsv(
        hits_tsv,
        all_mer_hits,
        ["sample", "source_file", "feature_type", "gene", "start",
"end", "strand", "locus_tag", "product"],
    )
    write_tsv(
        non_mer_tsv,
        all_non_mer_mercury_hits,
        ["sample", "source_file", "feature_type", "gene", "start",
"end", "strand", "locus_tag", "product"],
    )
    build_html(table_rows, mer_genes, html_path)

    # Also update the original simple table filenames so your existing
path shows
    # the new reference rows.
    legacy_html = OUTDIR / "mer_contig2_colored_table.html"
    legacy_tsv = OUTDIR / "mer_contig2_presence_absence.tsv"
    shutil.copyfile(html_path, legacy_html)
    shutil.copyfile(presence_tsv, legacy_tsv)

    print(f"Samples/plasmids included: {len(table_rows)}")
    print(f"mer genes detected across all files: {', '.join(mer_genes)
if mer_genes else 'none'}")
    print("Saved:")
    print(f"  {html_path}")
    print(f"  {presence_tsv}")
    print(f"  {hits_tsv}")
    print(f"  {non_mer_tsv}")
```

```
    print("Updated existing table path:")
    print(f"  {legacy_html}")


if __name__ == "__main__":
    main()
```
