# Supplementary material for: Conservation and plasticity of IncHI2 plasmid-borne metal resistance loci in Salmonella enterica Serovar Heidelberg
Source: Front Microbiol. 2026 Oct 28;17:1968489. doi: 10.3389/fmicb.2026.1968489 (PMC13752096; doi:10.3389/fmicb.2026.1968489)
Supplement: Supplementary file 2 [file Table_1.doc]

**Supporting Table S1.** IncHI2A Plasmid types detected in NCTR isolates based on MOB-suite replicon/mobility classification.

| **Isolate** | **Plasmid type** | **Size** | **Mobility** | **Cluster** |
| --- | --- | --- | --- | --- |
| R478 | IncHI2 | 274,762 bp | conjugative | reference |
| pSH111_227 | IncHI2/FIB-like | 227,608 bp | conjugative | reference |
| NCTR117 | IncHI2A | 261,448 bp | conjugative | AA738/AJ049 |
| NCTR122 | IncHI2A | 233,864 bp | conjugative | AA738/AJ049 |
| NCTR136 | IncHI2A | 231,816 bp | conjugative | AA738/AJ049 |
| NCTR137 | IncHI2A | 232,580 bp | conjugative | AA738/AJ049 |
| NCTR718 | IncHI2A | 268,247 bp | conjugative | AA739/AJ058 |
| NCTR719 | IncHI2A | 268,250 bp | conjugative | AA739/AJ058 |
| NCTR830 | IncHI2A | 235,025 bp | conjugative | AA738/AJ049 |
| NCTR938 | IncHI2A | 238,185 bp | conjugative | AA738/AJ049 |
